# Probabilistic OPF and LFC of conventional with RES, energy storage and FACTS using DTBO

**DOI:** 10.1038/s41598-026-43847-4

**Published:** 2026-04-02

**Authors:** Adhit Roy, Susanta Dutta, Soumen Biswas, Anagha Bhattacharya, Siddhartha Ghosh, Sudipta Banerjee

**Affiliations:** 1 Department of Electrical Engineering, NIT, Mizoram, India; 2 Department of Electrical Engineering, Dr. B. C. Roy Engineering College, Durgapur, India; 3 Department of Electrical Engineering, Sanaka Educational Trust’s Group of Institutions, Durgapur, India; 4 Department of Computer Science Engineering, Symbiosis Institute of Technology, Symbiosis International (Deemed University), Pune, India

**Keywords:** Optimal Power Flow (OPF), Load Frequency Control (LFC), Renewable energy (wind and solar), Energy storage (AEFC and, FACTS (TCSC, TCPS and SVC) Driving Training Based Optimization (DTBO), Fractional order proportional integral derivative (FOPID), Energy science and technology, Engineering

## Abstract

With thermal power sources taken into account, the IEEE 57-bus and IEEE 118 bus system’s probabilistic optimal power flow (OPF) solution is being reached. In this article, incorporate renewable energy sources (RES), energy storage system (ESS) and flexible ac transmission system (FACTS) i.e thyristor controlled series compensator (TCSC), thyristor controlled phase shifter (TCPS) and Static VAR compensator (SVC) with frequency security-constrained into the OPF. The reduction of generation costs, emissions and frequency deviations are the main goals. Five situations have been studied in this article: OPF without frequency security restriction, probabilistic OPF with frequency security constraint, OPF integrating FACTS devices (TCSC, TCPS and SVC) with frequency security requirement, OPF incorporating RES (wind and PV), ESS (aqua electrolyzer fuel cell (AEFC) and ultra capacitor), FACTS with frequency security constraint comprises FOPID controller and OPF integrated RES, ESS on IEEE 118-bus system with frequency security constraint. In order to show the balance between generation and consumption, the system’s frequency needs to be kept within a safe range. Therefore, the power flow optimisation system should maintain frequency stability in addition to having the lowest generation cost under operational conditions. Frequency security is a new limitation on the power dispatch problem that is necessary to enable this approach. The test results show that using RES (wind and PV), ESS (AEFC and ultra capacitor) and FACTS with frequency security constraints improves the OPF problem’s resolution. The overall fuel cost and emissions are decreased by 16.59% and 34.95%, respectively, after integrating the FACTs device with security constraints. Additionally, by integrating RES, ESS, and FACTS with frequency security limitation using a FOPID controller, the overall fuel cost and emissions are lowered by 36.41% and 41%, respectively, at 50% load. Furthermore the overall fuel cost and emissions are decreased by 26.11% and 36% at 80% load. Driving training-based optimization (DTBO) has been utilized to identify the optimal solution. The experimental results show that the DTBO outperforms the biography based optimization (BBO) and grey wolf optimization (GWO). Statistical techniques like one-way ANOVA (analysis of variance) demonstrate that the recommended approach has yielded superior results.

## Introduction

For power system management and control, one of the most crucial techniques is the optimal power flow (OPF). OPF aims to maintain voltage stability, limit emissions, minimise power loss, and minimise producing costs while optimising the settings of various system constraints. The system performs optimally during the optimization process when the generator power, the power flow in the lines and the bus voltage are all within the required operating condition of the system. The power flow balance, the generator bus voltage and the power generator’s capabilities must all be closely monitored. The OPF problem is determining the best operating state for a power source to satisfy load-side demands while taking security and practical considerations into account. Frequency deviation will rise at lower loads as inertia constants become less of a major problem. For the system to keep the frequency within suitable limits, there must be sufficient inertia. However, the high degree of producing variation in modern power networks has resulted in a lack of inertia and regulation capability, which presents new challenges for frequency security^1,2^. Thus, frequency security must be considered in the OPF solution. Even while the least cost dispatch system frequently meets standard operating conditions, its limited ability to adjust frequency may lead to frequency security violations. The frequency stability constraint will be taken into account by the OPF to make sure that the system frequency variation maintains within a safe range under normal condition.

To solve the OPF problems, researchers have put out a number heuristic and traditional optimization algorithms during the last few decades. The slime mold algorithm (SMA) has been proposed by Khunkitti et al.^3^ to solve OPF problems by taking emission and cost functions. Islam et al.^4^ suggested an intelligence swarm-motivated salp swarm algorithm (SSA) that seeks to supply the load demand while minimizing fuel costs in OPF problem. Layth et al.^5^ proposed a novel upgrade of the differential evolution algorithm to lower generating costs, emissions and enhance voltage stability. For the OPF problem, Meng et al.^6^ introduced a novel crisscross search based grey wolf optimizer (CS-GWO) to minimize the fuel cost and voltage deviation. The improved salp swarm algorithm (ISSA) was proposed by Salma Abd el-sattar^7^ to address the OPF problem. The heap optimization technique (HOA) was adopted by Mohamed Shaheen^8^ to resolve the OPF issue. Ravi Kumar Avvari^9^ proposed a unique hybrid multi-objective evolutionary algorithm (MOEA) based on the invasive weed optimization (IWO) technique and decomposition for the OPF issue.In order to lower pollution, power losses, and fuel generating costs, Mallala Balasubbareddy^10^ devised a novel ameliorated ant lion optimization (AALO) algorithm. Khunkitti et al.^11^ introduced a hybrid dragonfly algorithm (DA) to solve multiobjective OPF problems in a power system to reduce emissions, losses and fuel expenses. Khamies et al.^12^ proposed an improved runge kutta optimizer and a suggested PID controller to boost the strategy’s performance and maintain grid stability in the presence of RESs and ESS such as fuel cell and EV systems. In order to successfully manage the challenging Probabilistic Optimal Power Flow (POPF) optimization problem in contemporary power grids, Shaheen et al.^13^ suggest a novel implementation of the Catch Fish Optimization approach (CFOA). For the optimal solution of the probabilistic optimal power flow (OPF), Shaheen et al.^14^ present a recently created circle search algorithm (CSA). In order to solve single-objective OPF (SOOPF), multi-objective OPF (MOOPF), and many-objective OPF (MaOPF) problems, Khunkitti et al.^15^ develop a many-objective marine predators algorithm (MaMPA). Khunkitti et al.^16^ developed a novel technique called two-archive Harris Hawk optimization (TwoArchHHO) to handle many-objective optimal power flow (MaOOPF) problems in order to enhance power system operation and management and meet modern power system needs. In distribution systems linked to distributed generations (DGs) where several BESSs are examined, Wichitkrailat et al.^17^ introduced a recently developed optimization algorithm called the crayfish optimization algorithm (COA) for the ideal sizing and placements of numerous BESSs. To address the dynamic economic dispatch problem using demand response programs (DRP), H Lotfi^18^ proposes a novel hybrid optimization technique that uses the PSO and modified shuffled frog leaping algorithm (MSFLA) in addition to ES and RES. A novel and effective approach based on the combined whale optimization algorithm and grey wolf optimizer is presented by Ghanbari et al.^19^ to solve the multi-area dynamic economic dispatch problem. The multi-objective profit-based unit commitment (PBUC) issue is solved by Lotfi et al.^20^ using a novel mutation-based modified version of the shuffling frog leaping algorithm (SFLA), which takes ESS and RES into consideration.

Numerous research articles on OPF solutions utilizing FACTS devices have been published throughout the last thirty years. Gouda et al.^21^ determine the OPF, which contains a unified power flow controller (UPFC), in order to minimize generating cost and loss. Mohammed et al.^22^ suggested the northern goshawk optimization (NGO) for solving OPF with FACTS in order to lower generation costs, power losses, and enhance the voltage profile of the system. An ideal TCSC tuning technique based on the grey wolf algorithm was proposed by Rambabu et al.^23^ to reduce power losses, fuel costs, emissions, and voltage volatility in a modern electrical network. In order to reduce costs and ensure voltage, Panda et al.^24^ proposed a modified bacteria foraging approach to solve the OPF problem with shunt facts devices on IEEE 30-bus systems. In order to solve the OPF problem with FACTS devices and unpredictable wind power, Duman et al.^25^ showed how well a modified hybrid particle swarm optimization (PSO) and gravitational search algorithm (PSOGSA) paired with chaotic maps worked. In a modified IEEE 30 bus-system, RESs and several FACTS devices were introduced by Biswas et al.^26^.

This work proposes RES (wind and PV), ESS (aqua electrolyzer, fuel cell and ultra capacitor) & FACTS (TCSC, TCPS and SVC) devices into the IEEE 57-bus and IEEE 118-bus systems with thermal energy, to decrease the generating cost and emission. In integrated RES (wind and PV) into power system, stabilizing frequency fluctuations becomes challenging task. In this present study, introduced frequency security constraint with FOPID controller to improve the frequency deviation. A new powerful optimization technique, driving training-based optimization (DTBO) is proposed in this study to solve the OPF problem. The proposed algorithm’s search agents exhibit a variety of social and individual traits to avoid becoming trapped in local optima and speed up convergence

**Fig. 1:**
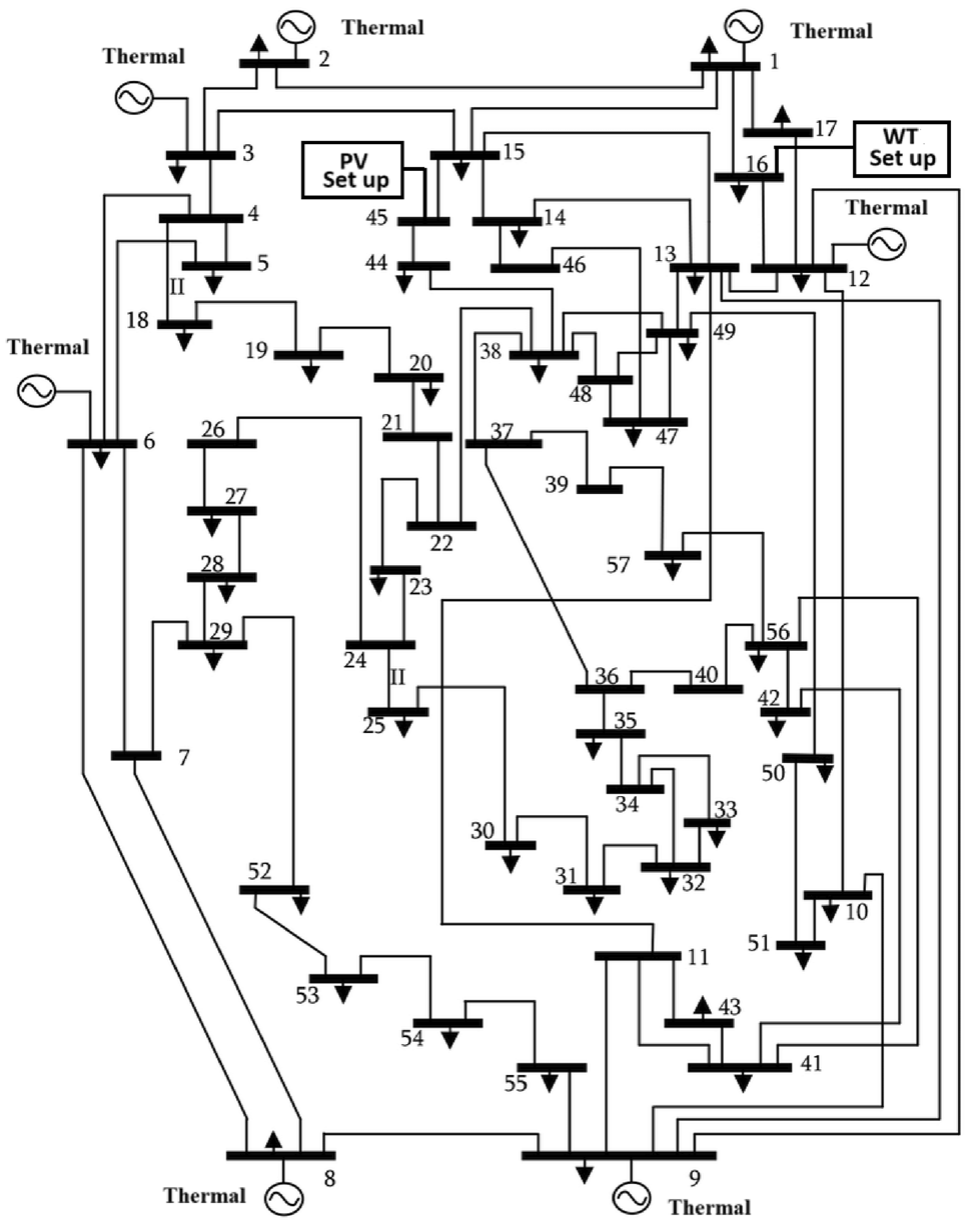
The schematic of the IEEE-57 Bus Network with thermal generator.

### Research gap of this literature review

The literature review highlights several research gaps in the context of OPF constraints caused by renewable-integrated power systems, particularly in relation to addressing the challenges posed by the integration of renewable energy sources (RES), such as solar, wind and the associated frequency stability issues. An overview of these gaps is as follows:

1. Combining OPF with frequency security: Frequency security limitations are not explicitly incorporated into the OPF architecture, despite the fact that current OPF methods concentrate on reducing fuel expenses, transmission losses and voltage disturbances. The majority of conventional OPF models, which are made for conventional energy systems, don’t completely account for how fluctuating RES affects frequency regulation. Significant frequency fluctuations are sometimes caused by the unpredictable nature of renewable energy sources, endangering the power system’s stability and dependability. Particularly in systems with substantial RES penetration, there is still a need for a new OPF formulation that incorporates frequency management and handles frequency variations.
2. Optimizing with metaheuristics for intricate OPF issues: Although a number of metaheuristic approaches, such as the grey wolf optimization (CS-GWO), crowd search algorithms (CSA), and the salp swarm algorithm (SSA), have been used to solve OPF problems, there are still no innovative optimization techniques that can handle the complexity of contemporary power systems that integrate RES. Specifically, the application of DTBO, a recently created algorithm, to OPF problems has not been fully explored. A significant research gap exists in the integration of RES while maintaining frequency stability in the use of DTBO in global optimization for OPF.
3. Nonlinearities in thermal power plants are not given enough attention: The nonlinearities in thermal power plants are not given enough consideration in traditional OPF frameworks, despite the fact that they contribute to the overall decline in system performance in interconnected power systems. It can make frequency instability worse when combined with the changeable nature of RES. To model and take these nonlinearities into account in OPF formulations for systems that incorporate RES, more research is required.
4. Difficulties in regulating frequency with RES and load inertia variability: A crucial difficulty in interconnected grids is how load inertia variations and RES integration affect frequency regulation. The intermittent nature of renewable energy sources and changeable load inertia have an impact on the frequency regulating process, which is frequently overlooked in current research. Research on dynamic frequency limitations that accommodate the unpredictability of renewable sources as well as conventional generating units is lacking. Forecasting of renewable energy, demand prediction and sophisticated management systems must be combined in new ways to ensure frequency stability in the face of these changes.
5. Inadequate evaluation of deregulated electricity networks: The majority of the literature currently available on OPF is based on centralized, regulated power systems. However, maintaining frequency stability becomes more difficult in a deregulated setting when numerous stakeholders and market operators share grid resources. Since the market structure frequently lacks coordination mechanisms for frequency regulation and control, integrating RES into such systems poses special operational issues. A novel approach to OPF that not only reduces costs but also guarantees system stability by offering sufficient frequency regulating mechanisms is required by deregulated market structures.
6. Unexplored potential for collaboration between optimization algorithms and sophisticated controllers: Although advanced controllers, such the fractional order proportional integral derivative (FOPID) controller, have demonstrated promise in frequency regulation, little is known about how well they work with OPF optimization techniques. The majority of research looks at optimization algorithms and controllers independently, without examining how they may work together to maximize performance in renewable-integrated power systems. In order to close this gap and improve the overall stability and operational efficiency of the system, creative research on the integration of optimization algorithms with sophisticated controllers is required.

### Contribution of the current research work

The present document illustrates the following research-motivating factors.

- The ideal solution for an IEEE 57-bus and IEEE 118-bus system with a thermal power plant has been investigated in this study.
- The FACTS (TCSC, TCPS, and SVC) devices have been integrated into the thermal plant to lower the cost of generation. Figure 1 depicts the IEEE 57-bus system’s scheduling model.
- Further reduce the generation cost and emission, RES (wind and PV), ESS (aqua electrolyzer, fuel cell and ultra capacitor) have been incorporated.
- The frequency deviation has grown as a result of including RES. FOPID controllers with frequency security constraints have been introduced to lower the frequency deviation.
- The literature review indicates that the current optimization strategies have several research gaps. To fill the existing research gaps, the proposed systems have been evaluated utilizing the recently established DTBO technique to find the ideal power system solution.
- Numerous one-objective functions, such as cost, emission, and frequency deviation minimisation, as well as multi-objective functions like cost and emission minimisation, have been examined.

## A systematic approach of the proposed system into AGC system

**Fig. 2:**
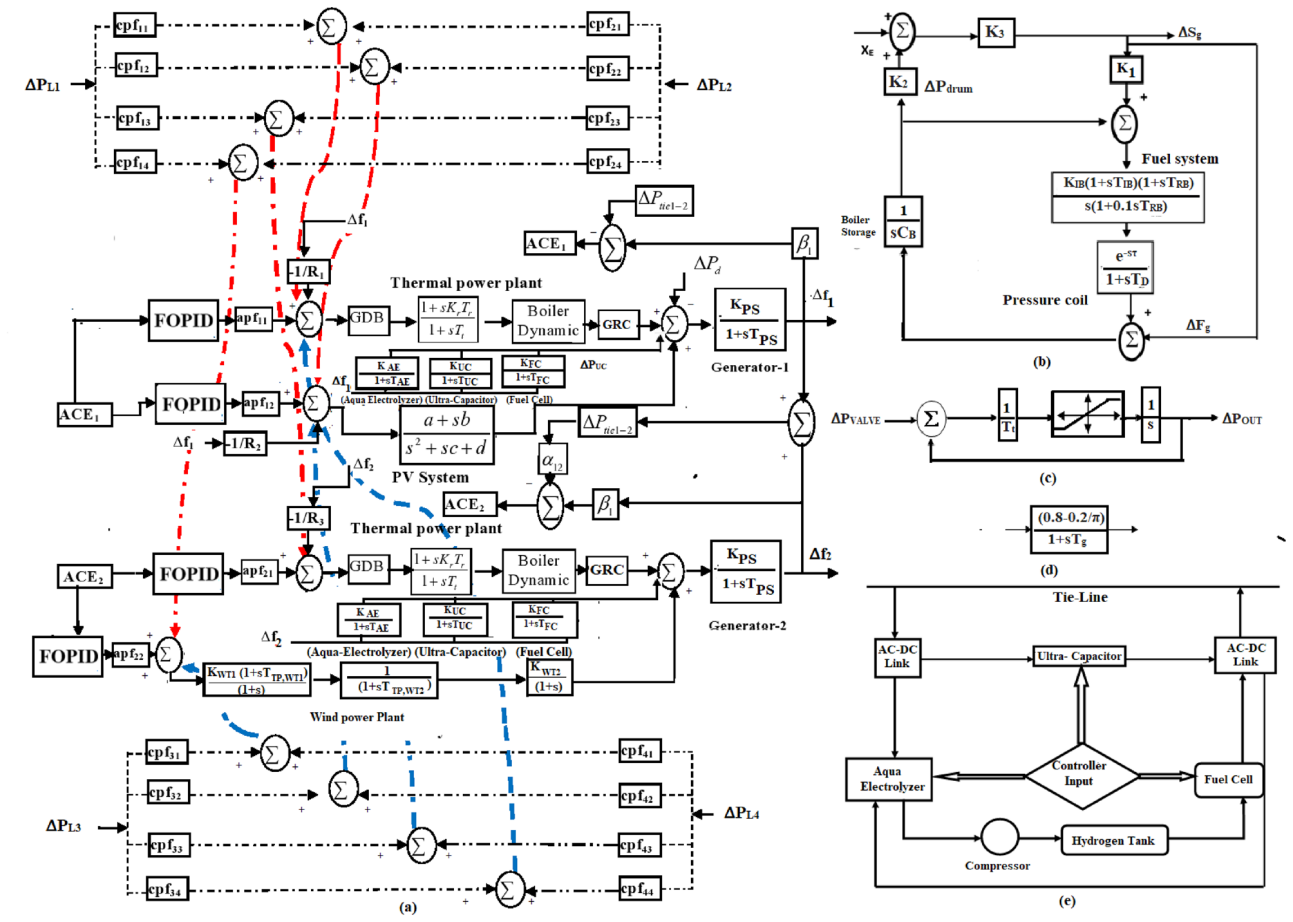
(**a**) Linearized model of Thermal-PV-wind system in deregulated scenario (test system-4). (**b**) Block diagram of boiler dynamics (test system-4).  (**c**) Block diagram of GRC (test system-4). (**d**) Block diagram of governor dead-band of test system 4. (**e**) Block diagram of control strategy of controller with AEFC and Ultra-capacitor.

In this work, the effectiveness of the proposed DTBO over the BBO and GWO approaches is assessed using a two-area renewable based multi source system (test system-4). The two-area renewable-based hybrid system utilizing wind and solar power is suggested under the deregulated scenario as seen in Fig. 2.

**Table 1:** Numerous cases are investigated in this paper.

Case	Single objective	Multi objective	Considered objectives	Constraints	Test system
1	$\surd$	X	Cost minimization for thermal plant with valve point effects		IEEE 57 Bus for only thermal without the frequency based security constraint.
2	$\surd$	X	Minimization of emission	Equity and inequity	
3	X	$\surd$	Simultaneous reduction of cost along the emissions		
4	$\surd$	X	Frequency deviation minimization		
5	$\surd$	X	Cost minimization for thermal plant with valve point effects		IEEE 57 Bus for only thermal with frequency based security
					constraint
6	$\surd$	X	Minimization of emission	Equity and inequity	
7	X	$\surd$	Simultaneous reduction of cost along the emissions		
8	$\surd$	X	Frequency deviation minimization		
9	$\surd$	X	Cost minimization for thermal plant with valve point effects integrating FACTS		IEEE 57 Bus for only thermal with FACTS (TCSC, TCPS and SVC) and frequency-based
10	$\surd$	X	Minimization of emission	Equity and inequity	Security constraints
11	X	$\surd$	Simultaneous reduction of cost along the emissions		
12	$\surd$	X	Frequency deviation minimization		
13	$\surd$	X	Cost minimization for thermal plant with valve point effects integrating RES,ESS and FACTS		IEEE 57 Bus with FACTS, RES (PV and Wind), Energy storage (AEFC and Ultra
14	$\surd$	X	Minimization of emission	Equity and inequity	Capacitor) and frequency
15	X	$\surd$	Simultaneous reduction of cost along the emissions		Based security constraints with FOPID controller
16	$\surd$	X	Frequency deviation minimization		
17	${\surd }$	X	Cost minimization for thermal plant with valve point effects integrating RES and ESS	Equity and inequity	IEEE 118 bus with RES (PV and Wind),ESS (AEFC and ultra capacitor) and frequency-based security constraints

**Table 2:** An outline of the IEEE 57-bus System.

Items	Quantity	Details
Buses	57	^27^
Branches	80	^27^
Thermal generators	7	Buses:1 (swing), 2, 3, 6, 8, 9 and 12
Wind generator	1	Bus 16
PV unit	1	Bus 45
AEFC	1	Bus 19
UC	1	Bus 20
Tap changing transformer	17	Branches: 19, 20, 31, 35, 36, 37, 41, 46, 54, 58, 59, 65, 66, 71, 73, 76 and 80
Control variables	33	Scheduled real power for 7 Nos. bus voltages of all generator buses (7Nos.) transformer tap setting and compensation devices
Load demand		1250.8 MW, 336.4 MVAr
Range of load bus voltage	50	[0.94–1.06]*p*.*u*.
Compensation devices	3	Buses: 18, 25 and 53

In this instance, one of the control areas has a solar unit combined with traditional thermal units. In the second area, the wind is combined with the thermal unit. Additionally, some nonlinearities like GRC and GDB in the thermal unit are included to test the effective operation of the suggested DTBO tuned FOPID controller in a realistic setting. The study is thus described as a two-area hybrid system based on renewable energy in the deregulated scenario. Historically, integrated renewable energy (wind, solar) sources in the large grid have led to a rise in frequency variation. Thus, a multi-source AGC system’s dynamic performance may be deteriorated by an interconnected system. The dynamic model of a multi-area AGC system in a deregulated scenario is described simply in this section. By adding the controller to the control loop, the traditional two-area hybrid model is transformed into a hybrid model.

A deregulated environment with independent system operators, distribution and transmission companies, and generating companies (GENCOs) is taken into consideration in the two-area system indicated above. The disco participation matrix (DPM), which illustrates the contract criteria between the GENCO and the DISCO, can represent every possible combination of power contracts between GENCOs and DISCOs^28^. The DPM can be defined by (1) (Tables 1, 2).

1 $$\begin{aligned} {\text {DPM}=\left[ \begin{array}{llllll} {{{f}_{11}}} & {{{f}_{12}}} & {{{f}_{13}}} & {{{f}_{14}}} & {{{f}_{15}}} & {{{f}_{16}}}\\ {{{f}_{21}}} & {{{f}_{22}}} & {{{f}_{23}}} & {{{f}_{24}}}& {{{f}_{25}}}& {{{f}_{26}}} \\ {{{f}_{31}}} & {{{f}_{32}}} & {{{f}_{33}}} & {{{f}_{34}}}& {{{f}_{35}}}& {{{f}_{36}}} \\ {{{f}_{41}}} & {{{f}_{42}}} & {{{f}_{43}}} & {{{f}_{44}}}& {{{f}_{45}}}& {{{f}_{46}}}\\ {{{f}_{51}}}& {{{f}_{52}}}& {{{f}_{53}}}& {{{f}_{54}}}& {{{f}_{55}}}& {{{f}_{56}}}\\ {{{f}_{61}}}& {{{f}_{62}}}& {{{f}_{63}}}& {{{f}_{64}}}& {{{f}_{65}}}& {{{f}_{66}}} \\ \end{array} \right] } \end{aligned}$$

All coefficients in the DPM matrix that are displayed in the column position should add up to unity, as given by (2).

2 $$\begin{aligned} & \sum \limits _{\text {i}=\text {1}}^{\text {NGENCO}}{\text {C}{{\text {P}}_{\text {ij}}}=\text {1;~for}}\ \ \text {j}=\text {1, 2, 3}\ldots \text {NDISCO} \end{aligned}$$

3 $$\begin{aligned} & \!\!\Delta \!\!{{\text {P}}_{\text {tie1}-\text {2,3schedule}}}=\sum \limits _{\text {i}=\text {1}}^{\text {{n}}}{\sum \limits _{\text {j}=\text {1}}^{\text {{m}}}{\text {c}{{\text {p}}_{\text {ij}}}\!\!\Delta \!\! {{\text {P}}_{\text {Lj}}}-\sum \limits _{\text {i}=\text {1}}^{\text {{n}}}{\sum \limits _{\text {j}=\text {1}}^{\text {{m}}}{\text {c}{{\text {p}}_{\text {ij}}}\!\!\Delta \!\!{{\text {P}}_{\text {Lj}}}}}}} \end{aligned}$$

where NGENCO = no. of generation company, NDISCO = no. of distribution company, $\Delta {{P}_{tie1-2,schedule}}$ = (GENCO in area 1 transfers power to DISCO in area 2) - (GENCO in area-2 transfers power to DISCO in area-1) and $\Delta {{P}_{tie1-3,schedule}}$ = (GENCO in area 1 transfers power to DISCO in area 3) - (GENCO in area-3 transfers power to DISCO in area-1). Here n=1 and m=2. Note that at steady state, the tie-power errors are given by (4).

4 $$\begin{aligned} \Delta {{\text {P}}_{\text {tie} \text {1}-\text {2, error}}}=\Delta {{\text {P}}_{\text {tie}\text {1}-\text {2,schedule}}}-\Delta {{\text {P}}_{\text {tie}\text {1}-\text {2,actual}}} \end{aligned}$$

When all the areas are in control, the above-described area control errors must be zero. The area control error (ACE) of the system is given by:

5 $$\begin{aligned} & {{\text {e}}_{\text {1}}}\text {(t)}=\text {AC}{{\text {E}}_{\text {1}}}={{\text {B}}_{\text {1}}}\,\!\!\,\,\Delta \!\!\,\,{{\text {f}}_{\text {1}}}+\!\!\,\,\Delta \!\!\,\,{{\text {P}}_{\text {tie1}-\text {2,error}}} \end{aligned}$$

6 $$\begin{aligned} & {{\text {e}}_{\text {2}}}\text {(t)}=\text {AC}{{\text {E}}_{\text {2}}}={{\text {B}}_{\text {2}}}\!\!\,\,\Delta \!\!\,\,{{\text {f}}_{\text {2}}}+\!\!\,\,\Delta \!\!\,\,{{\alpha \text {P}}_{\text {tie2}-\text {1,error}}} \end{aligned}$$

## Model

### Wind power model

The variation of wind speed (*vm*/*s*) is adequately described by the two-parameters (scale factor $\xi$’ and shape factor $\kappa$’) of weibull PDF as follows^29,30^:

7 $$\begin{aligned} f\left( v \right) =\left( \frac{\kappa }{\xi } \right) \times {{\left( \frac{v}{\xi } \right) }^{\kappa -1}}\times \left( {{e}^{-\left( \frac{v}{\xi } \right) }}^{\kappa } \right) \quad \quad 0<v<\infty \end{aligned}$$

The following factors are used to calculate a wind turbine’s output power: cut-in speed ${{v}_{in}}$, rated speed ${{v}_{r}}$, cut-out speed ${{v}_{out}}$ and rated output ${{P}_{wr}}$

8 $$\begin{aligned} {{{P}_{w}}(v)=\left\{ \begin{array}{ll} 0& for\,v<{{v}_{in}}\ \varsigma \ v>{{v}_{out}} \\ {{P}_{wr}}\left( \frac{v-{{v}_{in}}}{{{v}_{r}}-{{v}_{in}}} \right) \quad \quad & for\ {{v}_{in}}\le v\le {{v}_{r}} \\ {{P}_{wr}} & for\ {{v}_{r}}<v\le {{v}_{out}} \\ \end{array} \right. } \end{aligned}$$

For a given wind speed zone, the probability of wind power may now be expressed using the following formula:

9 $$\begin{aligned} & f{{\left( {{P}_{w}} \right) }_{\left| {{P}_{w}}=0 \right. }}=1-\exp \left[ -{{\left( \frac{{{v}_{in}}}{\xi } \right) }^{\kappa }} \right] +\exp \left[ -{{\left( \frac{{{v}_{out}}}{\xi } \right) }^{\kappa }} \right] \end{aligned}$$

10 $$\begin{aligned} & f{{\left( {{P}_{w}} \right) }_{\left| {{P}_{w}}={{P}_{wr}} \right. }}=\exp \left[ -{{\left( \frac{{{v}_{r}}}{\xi } \right) }^{\kappa }} \right] -\exp \left[ -{{\left( \frac{{{v}_{out}}}{\xi } \right) }^{\kappa }} \right] \end{aligned}$$

11 $$\begin{aligned} & f{{\left( {{P}_{w}} \right) }_{\left| 0<{{P}_{w}}<{{P}_{wr}} \right. }}=\left[ \frac{\kappa \times \left( {{v}_{r}}-{{v}_{in}} \right) }{{{\xi }^{\kappa }}\times {{P}_{wr}}} \right] \times {{\left[ {{v}_{in}}+\left( \frac{{{P}_{w}}}{{{P}_{wr}}} \right) \left( {{v}_{r}}-{{v}_{in}} \right) \right] }^{\kappa -1}} \nonumber \\ & \quad \times \exp \left[ -{{\left( \frac{{{v}_{in}}+\left( \frac{{{P}_{w}}}{{{P}_{wr}}} \right) \times \left( {{v}_{r}}-{{v}_{in}} \right) }{\xi } \right) }^{\kappa }} \right] \end{aligned}$$

**Fig. 3:**
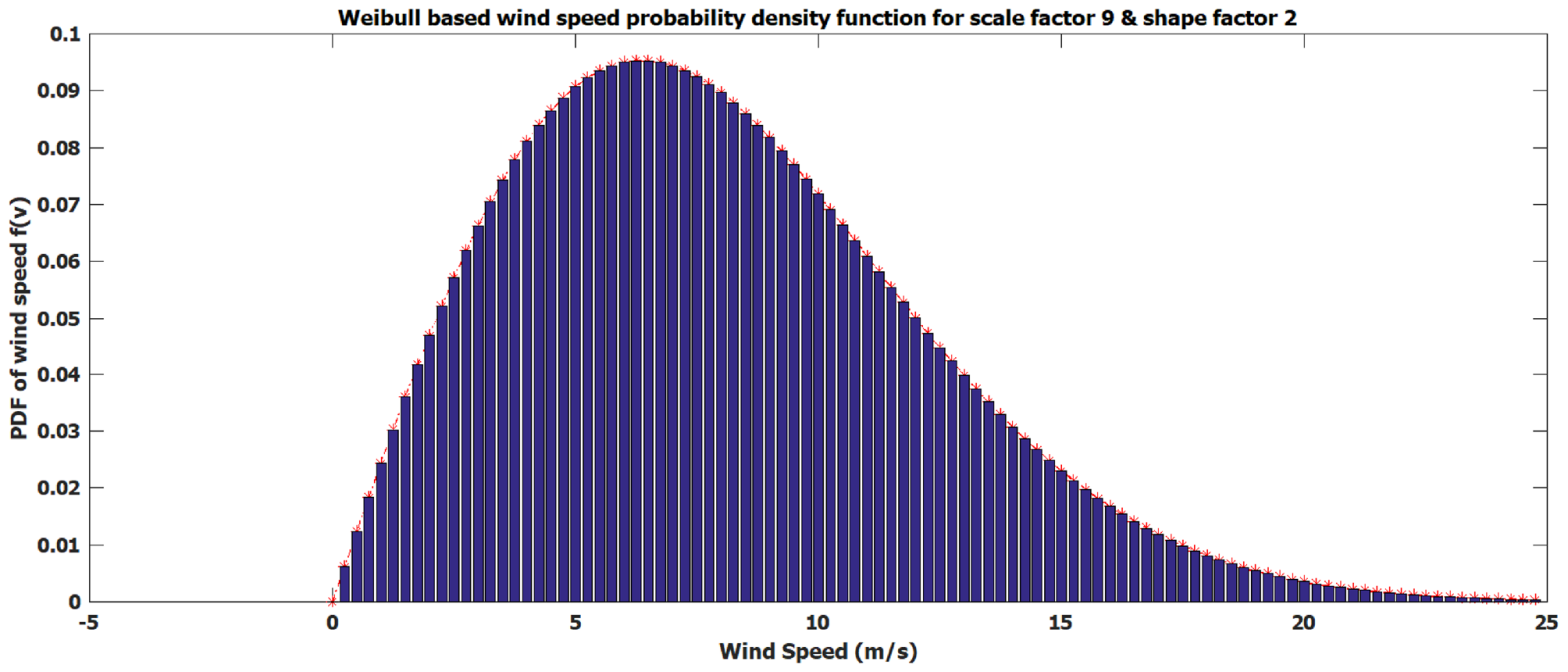
Weibul based wind velocity PDF.

#### Determination of wind cost

The unpredictability of the wind will affect when wind-producing units should be added to the system during times of high demand. Because wind speed near the shore is unpredictable, there is uncertainty in the generation of power. Figure 3, which displays Weibull’s probability density function, will be used to analyze the expected uncertainty costs related to wind energy. The definitions of this function are overestimation and underestimation (12).

12 $$\begin{aligned} {\left\{ \begin{array}{ll} & {{TotalCost}_{wind}}={\sum \limits _{m=1}^{{{N}_{wind}}}{{Cost}_{windm}}\left( {{P}_{windm}}\right) } \\ & = {\sum \limits _{m=1}^{{{N}_{wind}}}{\left( {{Cost}^{O}_{windm}}+{{Cost}^{U}_{windm}} \right) }} \\ \end{array}\right. } \end{aligned}$$

where ${{TotalCost}_{wind}}$ denotes the total wind cost and ${{N}_{wind}}$ denotes the total number of wind units.

Wind overestimation cost calculation: The cost of overestimation is expressed as the difference between the actual power and the anticipated power output. This implies that the power produced by wind will surpass the load requirement. The spinning reserve will supply the additional power required to fulfill the load requirement. It is possible to calculate overestimation costs using (13).

13 $$\begin{aligned} {\left\{ \begin{array}{ll} & {{{Cost}^{O}_{windm}}={{Pf}^{O}_{windm}}\times {{P}_{windm}}\left[ 1-e^{ -{{\left( \frac{{{V}_{in}}}{{{s}}} \right) }^{{{j}}}}} +e^{ -{{\left( \frac{{{V}_{out}}}{{{s}}} \right) }^{{{j}}}}}\right] + } \\ & {\left( \frac{{{P}_{wratedm}}{{V}_{in}}}{{{V}_{rated}}-{{V}_{in}}}+{{P}_{windm}} \right) \left[ e^{ -{{\left( \frac{{{V}_{in}}}{{{c}}} \right) }^{{{j}}}}}-e^{ -{{\left( \frac{{{V}_{in}}+{{P}_{windm}}\frac{{{V}_{rated}}-{{V}_{in}}}{{{P}_{wrated}}}}{{{s}}} \right) }^{{{j}}}} } \right] } \\ & {\,+\left( \frac{{{P}_{wrated}}{s}}{{{V}_{rated}}-{{V}_{in}}}\right) \left[ \zeta \left\{ 1+\frac{1}{{{j}}},{{\left( \frac{{{V}_{in}}+{{P}_{windm}}\frac{{{V}_{rated}}-{{V}_{in}}}{{{P}_{wrated}}}}{{{s}}} \right) }^{{{j}}}} \right\} -\zeta \left\{ 1+\frac{1}{{{j}}},{{\left( \frac{{{V}_{in}}}{{{s}}} \right) }^{{{j}}}} \right\} \right] } \\ \end{array}\right. } \end{aligned}$$

Wind underestimation cost calculation: Underestimation expenses occur when actual wind energy exceeds projections. In order to prevent the loss of power generated by wind turbines, any extra electrical energy will be stored in batteries. The formula for determining the underestimate cost is shown below (14):

14 $$\begin{aligned} {\left\{ \begin{array}{ll} & {{Cost}^{U}_{windm}}= {{Pf}^{U}_{windm}}\times \left( {{P}_{wrated}}-{{P}_{windm}} \right) \left[ e^{ -{{\left( \frac{{{V}_{rated}}}{{{s}}} \right) }^{{{j}}}}}-e^{ -{{\left( \frac{{{V}_{out}}}{{{s}}} \right) }^{{{j}}}}} \right] + \\ & {\left( \frac{{{P}_{wrated}}{{V}_{in}}}{{{V}_{rated}}-{{V}_{in}}}+{{P}_{windm}} \right) \left[ e^{ -{{\left( \frac{{{v}_{rated}}}{{{s}}} \right) }^{{{j}}}} }-e^{ -{{\left( \frac{{{V}_{in}}+{{P}_{windm}}\frac{{{v}_{rated}}-{{v}_{in}}}{{{P}_{wrated}}}}{{{s}}} \right) }^{{{j}}}}} \right] } \\ & { +\frac{{{P}_{wrated}}{{s}}}{{{V}_{rated}}-{{V}_{in}}}\left[ \zeta \left\{ 1+\frac{1}{{{j}}},{{\left( \frac{{{V}_{in}}+{{P}_{windm}}\frac{{{V}_{rated}}-{{V}_{in}}}{{{P}_{wrated}}}}{{{s}}} \right) }^{{{j}}}} \right\} -\zeta \left\{ 1+\frac{1}{{{j}}},{{\left( \frac{{{V}_{rated}}}{{{s}}} \right) }^{{{j}}}} \right\} \right] }\\ \end{array}\right. } \end{aligned}$$

In the above equations overestimation and underestimation cost of ${m^{th}}$ wind unit signified with ${{Cost}^{O}_{windm}}$ and ${{Cost}^{U}_{windm}}$; rated o/p power and rated velocity denoted by ${{P}_{wrated}}$ and ${{V}_{rated}}$; ${{V}_{in}}$ and ${{V}_{out}}$ are cut-in and cut-out velocity of wind; ${{Pf}^{U}_{windm}}$ is underestimation and ${{Pf}^{O}_{windm}}$ is overestimation cost co-efficient respectively.

### Solar power model

**Fig. 4:**
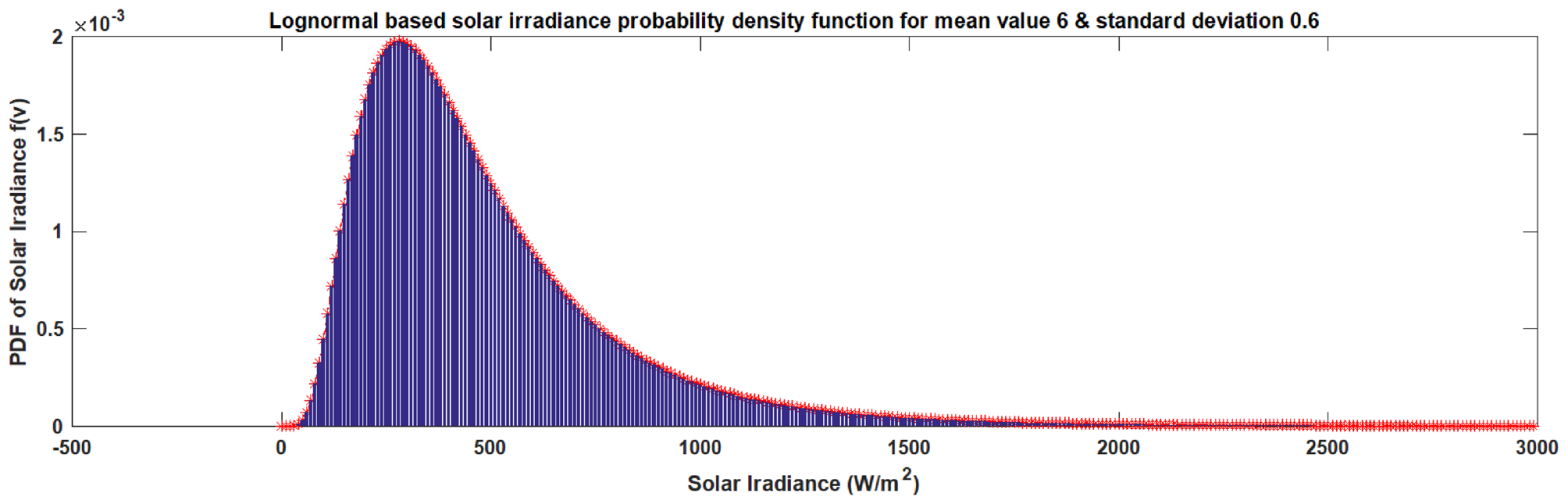
Lognormal based solar irradiance PDF.

The solar power unit converts solar energy into electrical energy. Solar irradiation and other weather factors affect power output. The lognormal probability distribution function based on solar irradiation is shown in Fig. 4. With a lognormal PDF *L*(*I*), In essence, the sun irradiance (I) probability distribution is closed^31,32^. As a result, it is commonly used to determine sun irradiation and is displayed as:

15 $$\begin{aligned} L\left( I \right) =\frac{1}{I\lambda \sqrt{2\pi }}\exp \left( \frac{-{{\left( \ln I-\varepsilon \right) }^{2}}}{2{{\lambda }^{2}}} \right) \quad ,\ \ I>0 \end{aligned}$$

Here, $\varepsilon$: mean of *I* distribution; $\lambda$: Standard deviation. The following illustrates the link between solar irradiation and the electrical output power of the PV unit:

16 $$\begin{aligned} P\left( I \right) =\left\{ \begin{array}{ll} {{P}_{nm}}\frac{{{I}^{2}}}{{{I}_{st}}{{I}_{c}}},\quad for\ 0<I<{{I}_{c}} \\ {{P}_{nm}}\frac{I}{{{I}_{st}}},\quad for\,I\ge {{I}_{c}} \\ \end{array} \right. \end{aligned}$$

Here, ${{P}_{nm}}$: nominal output power of PV unit; ${{I}_{st}}$: standard solar irradiance; ${{I}_{c}}$: critical irradiance point.

#### Solar cost calculation

The cost of energy generation for a solar unit is calculated by adding three different cost functions. These are listed below:citebiswas2017optimal:

17 $$\begin{aligned} {{Cost}_{solarl}}\left( {{P}_{solarl}}\right) ={{Cost}^{d}_{solarl}}+{{Cost}^{O}_{solarl}}+{{Cost}^{U}_{solarl}} \end{aligned}$$

Here overestimation cost and underestimation cost are denoted with ${{Cost}^{d}_{solarl}}$, ${{Cost}^{O}_{solarl}}$ and ${{Cost}^{U}_{solarl}}$ of the ${l^{th}}$ solar unit.

Solar direct cost: Direct costs are the expenditures made when producing solar energy. The following equation provides the solar energy’s direct cost.

18 $$\begin{aligned} {{Cost}^{d}_{solarl}}={d^{solar}_l}{{P}_{solarshl}},\,\,\,\,\,where\,\,\,\,\,\,l=1,2,3..,{{n}_{s}} \end{aligned}$$

Here, ${d^{s}_l}$ represents coefficients of direct cost and ${{P}_{solarshl}}$ and schedule power of the ${l^{th}}$ solar.

Solar overestimation cost:

In the event that solar power availability falls short of the planned amount, the overestimation cost is computed using the following formula.

19 $$\begin{aligned} {\left\{ \begin{array}{ll} & {{Cost}^{O}_{solarl}}={{PF}^{O}_{solarl}}\left( {{P}_{solarshl}}-{{P}_{solaravl}}\right) \\ & \,\,\,\,\,\,\,\,\,\,\,\,\,\,=\,{{PF}^{O}_{solarl}}\int \limits _{0}^{{{P}_{solarshl}}}{\left( {{P}_{solarshl}}-P_{solar}\right) {{f}_{P_{solar}}}(P_{solar})~dP_{solar}} \\ \end{array}\right. } \end{aligned}$$

where PDF of the power output of solar unit signifies with ${{f}_{p_{s}}}(P_{solar})$ ; ${{P}_{solarshl}}$, ${{P}_{solaravl}}$ and ${{PF}^{O}_{solarl}}$ are the scheduled power, average power and overestimation penalty cost coefficient of the ${l^{th}}$ solar unit. Solar underestimation cost: If the available solar power exceeds the scheduled power, the underestimated cost of the ${l^{th}}$ solar unit is calculated using the formula below.

20 $$\begin{aligned} {\left\{ \begin{array}{ll} & {{Cost}^{U}_{solarl}}={{PF}^{U}_{solarl}}\left( {{P}_{solaravl}}-{{P}_{solarshl}}\right) \\ & \,\,\,\,\,\,\,\,\,\,\,\,\,\,=\,{{PF}^{U}_{solarl}}\int \limits _{{{P}_{solarshl}}}^{{{P}_{solarrl}}}{\left( P_{s}-{{P}_{solarshl}}\right) {{f}_{p_{s}}}(P_{solar})~dP_{Solar}} \\ \end{array}\right. } \end{aligned}$$

where ${{P}_{srl}}$ and ${{PF}^{U}_{sl}}$ are the rated power and underestimation penalty cost coefficient of the ${l^{th}}$ solar unit.

### Energy storage system

#### Aqua electrolyzer with fuel cell

AEFC frequently acts as an auxiliary power source to meet long-term power supply demands, much like hydrogen energy^33^. To find different power, *AE* mostly employs electrolysis with strong hydrogen compression. A fuel cell’s *FC*, consumes the stored ${{H}_{2}}$ and instantly generates power to meet load needs. Transfer function of the Aquya-electrolyzer is defined as:

21 $$\begin{aligned} {{\text {G}}_{\text {AE}}} = \frac{{{K_{AE}}}}{{1 + s{T_{AE}}}} \end{aligned}$$

Transfer function of the Fuel-Cell may be represented as:

22 $$\begin{aligned} {{\text {G}}_{\text {FC}}}= \frac{{{K_{FC}}}}{{1 + s{T_{FC}}}} \end{aligned}$$

#### Ultra-capacitor

To balance load demand and power generation, a deregulated system can receive electricity from a super capacitor, also called an electro-mechanical double layer ultra-capacitor. Consequently, *UC* helps to minimize transient oscillation and satisfy the exact load demand. The energy density of *UC* is significantly higher than that of an electrolytic capacitor. *UC*’s stored energy is computed as

23 $$\begin{aligned} {{\text {V}}_{\text {UC}}} = 0.5C(V_{initial}^2 - V_{final}^2) \end{aligned}$$

where *C* represents capacitor in faraday, and ${{V}_{initial}}$ and ${{V}_{final}}$ are the initial and final voltage level of the ultra capacitor. The transfer function of the ultra capacitor is given by:

24 $$\begin{aligned} {{\text {G}}_{\text {UC}}} =\frac{{{K_{UC}}}}{{1 + s{T_{UC}}}} \end{aligned}$$

### FACTS devices

Shunt compensation devices (SVC) and series compensation devices (TCSC and TCPS) are described below^25,34–36^

#### Thyristor controlled series compensator (TCSC)

**Fig. 5:**
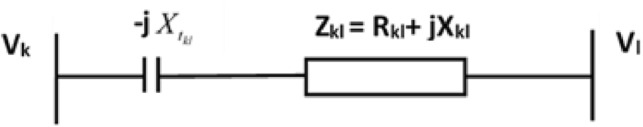
Model of TCSC between *k* and *l* bus.

The insertion of controllable reactance in a transmission line may represent the consequence of TCSC on a system. When a transmission line has compensated the flow of active power can be kept under a specified level for a wide range of operations. The static model of TCSC fitted between the buses *k* & *l* is displayed in Fig. 5. The branch with TCSC’s power flow equations^37^ can be obtained in the manner described below:

25 $$\begin{aligned} & {{P}_{k\,l}}=V_{k}^{2}{{G}_{k\,l}}-{{V}_{k}}{{V}_{l}}\left( {{G}_{k\,l}}\cos {{\partial }_{k\,l}}+{{B}_{k\,l}}\sin {{\partial }_{k\,l}} \right) \end{aligned}$$

26 $$\begin{aligned} & {{Q}_{k\,l}}=-V_{k}^{2}{{B}_{k\,l}}-{{V}_{k}}{{V}_{l}}\left( {{G}_{k\,l}}\sin {{\partial }_{k\,l}}-{{B}_{k\,l}}\cos {{\partial }_{k\,l}} \right) \end{aligned}$$

where ${{G}_{kl}}=\frac{{{R}_{kl}}}{{{R}_{kl}}^{2}+{{\left( {{X}_{kl}}-{{X}_{{{t}_{kl}}}} \right) }^{2}}}$,${{B}_{kl}}=\frac{\left( {{X}_{kl}}-{{X}_{{{t}_{kl}}}} \right) }{{{R}_{kl}}^{2}+{{\left( {{X}_{kl}}-{{X}_{{{t}_{kl}}}} \right) }^{2}}}$ within bus *k* & *l*, the flow of active & reactive power are ${{P}_{kl}}$, ${{Q}_{kl}}$ respectively; the voltage magnitude at bus *k* & *l* are ${{V}_{k}},{{V}_{l}}$, respectively; the phase difference between the two bus (*i*.*e*. *k* & *l*) voltages is ${{\partial }_{k\,l}}$; ${{R}_{kl}}$ is resistance and ${{X}_{kl}}$ is the reactance of transmission line which is placed between the buses *k* & *l*; the placed (within buses *k* & *l*) TCSC has reactance ${{X}_{{t}_{kl}}}$.

#### Thyristor controlled phase shifter (TCPS)

**Fig. 6:**
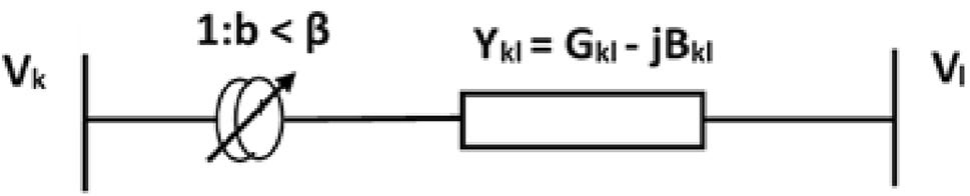
Model of TCPS between *k* and *l* buses.

Figure 6 represents the TCPS static model reside between the $k^{th}$ and the ${{l}^{th}}$ bus where $1:b\angle \beta =\left[ 1:{{b}_{r}}+j{{b}_{i}} \right]$ and ${{Y}_{kl}}=\left( {{G}_{kl}}-j{{B}_{kl}} \right)$ are tapping ratio & series admittance of the transformer.

The flows of real and reactive power^38^ from bus *k* to bus *l* are given by:

27 $$\begin{aligned} & {{P}_{kl}}={{b}^{2}}V_{k}^{2}{{G}_{kl}}-b{{V}_{k}}{{V}_{l}}\left[ {{G}_{kl}}\cos \left( {{\partial }_{kl}}+\beta \right) +{{B}_{kl}}\sin \left( {{\partial }_{kl}}+\beta \right) \right] \end{aligned}$$

28 $$\begin{aligned} & {{Q}_{kl}}=-{{b}^{2}}V_{k}^{2}{{B}_{kl}}-b{{V}_{k}}{{V}_{l}}\left[ {{G}_{kl}}\sin \left( {{\partial }_{kl}}+\beta \right) -{{B}_{kl}}\cos \left( {{\partial }_{kl}}+\beta \right) \right] \end{aligned}$$

Likewise, the active and reactive power flow from bus *l* to bus *k* is represented as follows:

29 $$\begin{aligned} & {{P}_{lk}}=V_{l}^{2}{{G}_{kl}}-b{{V}_{k}}{{V}_{l}}\left[ {{G}_{kl}}\cos \left( {{\partial }_{kl}}+\beta \right) -{{B}_{kl}}\sin \left( {{\partial }_{kl}}+\beta \right) \right] \end{aligned}$$

30 $$\begin{aligned} & {{Q}_{lk}}=-V_{l}^{2}{{B}_{kl}}+b{{V}_{k}}{{V}_{l}}\left[ {{G}_{kl}}\sin \left( {{\partial }_{kl}}+\beta \right) +{{B}_{kl}}\cos \left( {{\partial }_{kl}}+\beta \right) \right] \end{aligned}$$

**Fig. 7:**
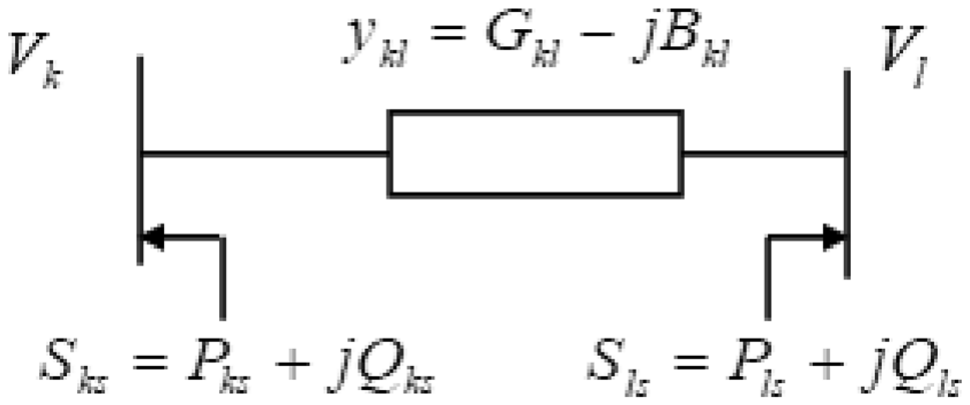
Injected power model of TCPS between *k* & *l* buses.

Figure 7 represents the TCPS injected power model. At buses *k* & *l*, the injected active and reactive powers of TCPS are respectively given by :

31 $$\begin{aligned} & {{P}_{ks}}=-{{b}^{2}}V_{k}^{2}{{G}_{kl}}-b{{V}_{k}}{{V}_{l}}\left[ {{G}_{kl}}\sin \left( {{\partial }_{kl}} \right) -{{B}_{kl}}\cos \left( {{\partial }_{kl}} \right) \right] \end{aligned}$$

32 $$\begin{aligned} & {{Q}_{ks}}={{b}^{2}}V_{k}^{2}{{B}_{kl}}+b{{V}_{k}}{{V}_{l}}\left[ {{G}_{kl}}\cos \left( {{\partial }_{kl}} \right) +{{B}_{kl}}\sin \left( {{\partial }_{kl}} \right) \right] \end{aligned}$$

33 $$\begin{aligned} & {{P}_{ls}}=-b{{V}_{k}}{{V}_{l}}\left[ {{G}_{kl}}\sin \left( {{\partial }_{kl}} \right) +{{B}_{kl}}\cos \left( {{\partial }_{kl}} \right) \right] \end{aligned}$$

34 $$\begin{aligned} & {{Q}_{ls}}=-b{{V}_{k}}{{V}_{l}}\left[ {{G}_{kl}}\cos \left( {{\partial }_{kl}} \right) -{{B}_{kl}}\sin \left( {{\partial }_{kl}} \right) \right] \end{aligned}$$

where ${{G}_{kl}}$ is the conductance & ${{B}_{kl}}$ is the susceptance of transmission line within the bus *k* & bus *l*.

#### Static VAR compensator (SVC)

**Fig. 8:**
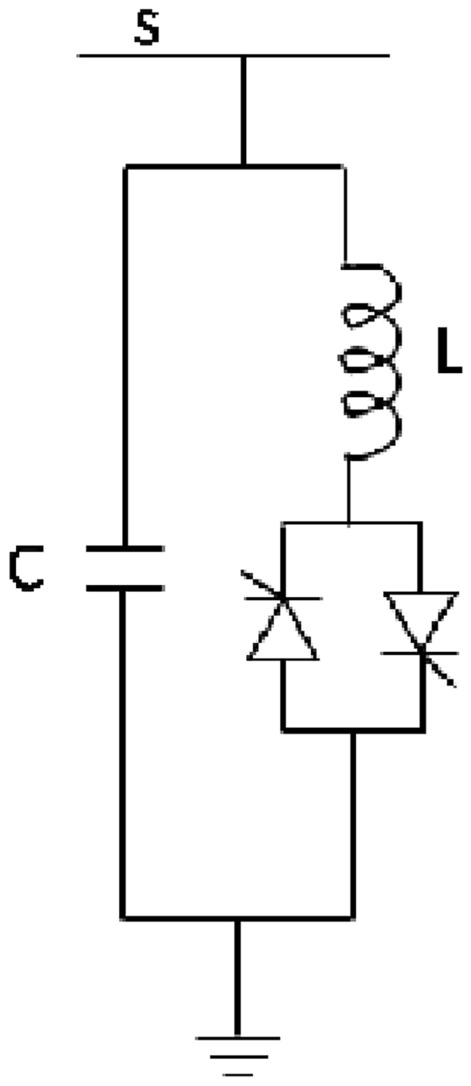
Basic SVC structure.

Figure 8 illustrates the fundamental structure of SVC. The corresponding susceptibility is demonstrated by^39^

35 $$\begin{aligned} {{B}_{eq}}={{B}_{L}}(\lambda )+{{B}_{C}} \end{aligned}$$

where,

36 $$\begin{aligned} {{{B}_{L}}(\lambda )=-\frac{1}{\omega L}\left( 1-\frac{2\lambda }{\pi }\right) \ \varsigma \ {{B}_{C}}=\omega \times C} \end{aligned}$$

The reactive power that is injected at buses is determined by:

37 $$\begin{aligned} {{Q}_{SVC}}=-V_{s}^{2}{{B}_{SVC}} \end{aligned}$$

The reactive power limitation at buses is

38 $$\begin{aligned} B_{SVC}^{\min }\le {{B}_{SVC}}\le B_{SVC}^{\max }. \end{aligned}$$

## Mathematical problem formulation

The OPF find the optimal control variable parameters for a given load by minimizing a predetermined objective function. OPF is a nonlinear constrained optimization problem that considers the operating constraints^40^ of the system. It can be expressed as follows:

Minimize

39 $$\begin{aligned} J(x,u) \end{aligned}$$

Subject to

40 $$\begin{aligned} g\left( x,u \right) =0 \end{aligned}$$

And

41 $$\begin{aligned} h\left( x,u \right) \le 0 \end{aligned}$$

where *J*(*x*, *u*) is the objective function, $g\left( x,u \right)$ is the set of equality constraints, $h\left( x,u \right)$ is the set of inequality constraints, u is known as control variables. x is known as state variables.

### Control variables

These are the variables that can be adjusted to meet the load flow calculations^41^. The control variables used to formulate the OPF problem are as follows: ${{P}_{G}}$: active power generation at the PV buses except at the slack bus. ${{V}_{G}}$: voltage magnitude at PV buses. T: tap settings of transformer. ${{Q}_{C}}$: shunt VAR compensation

42 $$\begin{aligned} {{u}^{T}}=\left[ {{P}_{{{G}_{2}}}}\cdots {{P}_{{{G}_{NG}}}},{{V}_{{{G}_{1}}}}\cdots {{V}_{{{G}_{NG}}}},{{Q}_{{{C}_{1}}}}\cdots {{Q}_{{{C}_{NC}}}},{{T}_{1}}\cdots {{T}_{NT}} \right] \end{aligned}$$

where NG, NT and NC stand for the no. of generators,regulating transformers and VAR compensator respectively.

### State variables

These variables define each unique state of the system.^41^. The OPF problem is formulated using the set of state variables that follows: ${{{P}_{G}}_1}$: active power at slack bus. ${{V}_{L}}$: magnitude of voltage at PQ buses. ${{Q}_{G}}$: reactive power generation of all generator units. ${{S}_{1}}$: transmission line loading. Hence, x (without FACTS devices) can be expressed as:

43 $$\begin{aligned} {{X}^{T}}=\left[ {{P}_{{{G}_{1}}}},{{V}_{{{L}_{1}}}}\cdots {{V}_{NL}},{{Q}_{{{G}_{1}}}}\cdots {{Q}_{{{G}_{NG}}}},{{S}_{{{l}_{1}}}}\cdots {{S}_{{{l}_{nl}}}} \right] \end{aligned}$$

u (with FACTS devices) can be expressed as:

44 $$\begin{aligned} {{u}^{T}}=\left[ {{P}_{{{G}_{1}}}},{{V}_{{{L}_{1}}}}\cdots {{V}_{NL}},{{Q}_{{{G}_{1}}}}\cdots {{Q}_{{{G}_{NG}}}},{{S}_{{{l}_{1}}}}\cdots {{S}_{{{l}_{nl}}}}, {{x}_{{{k}_{1}}}}\cdots {{x}_{{{k}_{nTCSC}}}}, {{\alpha }_{{{k}_{1}}}}\cdots {{\alpha }_{{{k}_{nTCPS}}}} \right] \end{aligned}$$

where NL stands for the no. of load buses and nl for the no. of transmission lines.

**Fig. 9:**
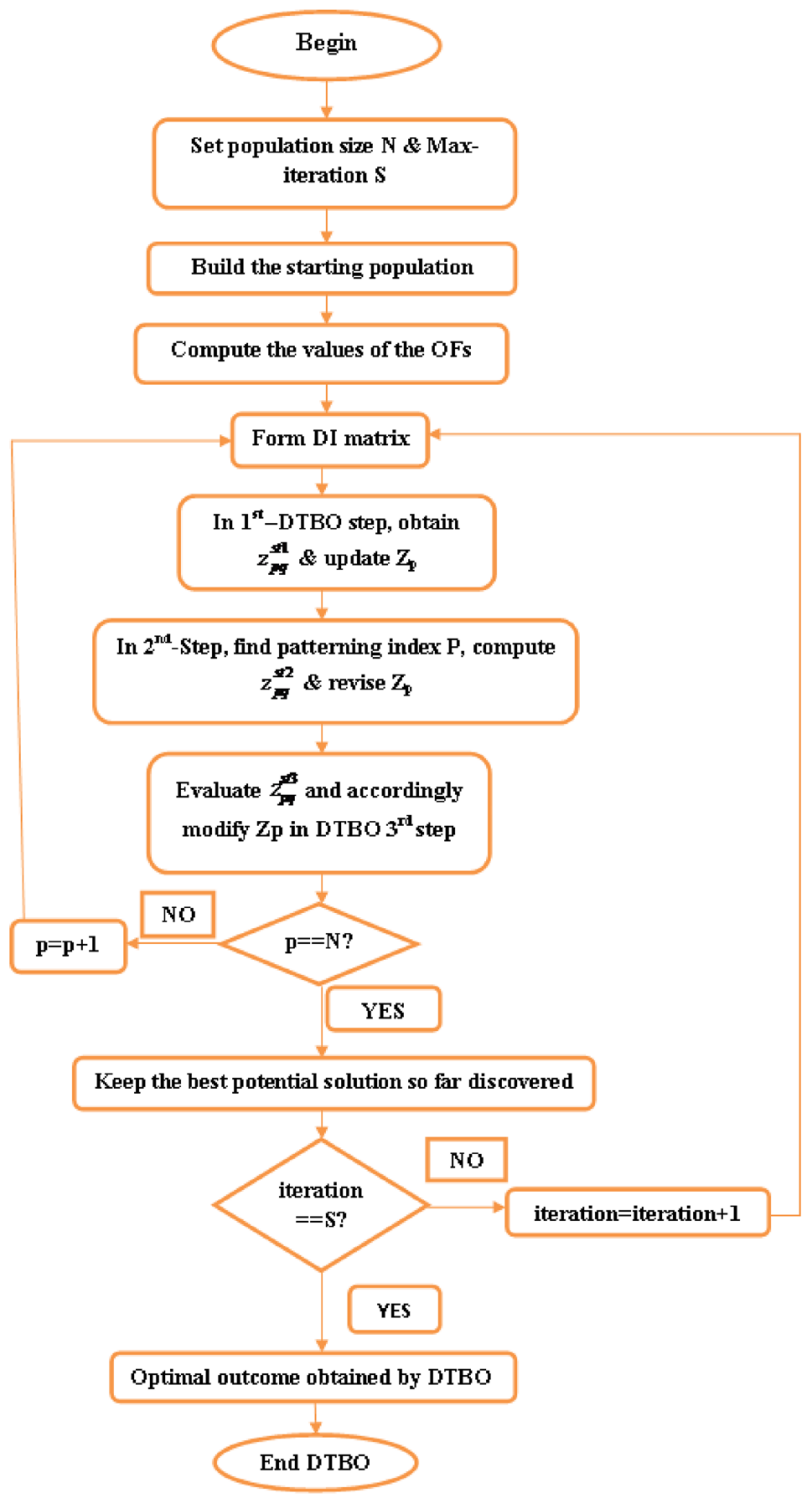
Flowchart of DTBO.

### Objective function

The OPF problem formulation takes into account generator terminal voltages, real power generation and multiple valve steam turbines for flexible operating facilities. The overall cost of generation^40^ for producing units with valve point effect is as follows:

#### Total generation cost

45 $$\begin{aligned} FC=\sum \limits _{i=1}^{NG}{{{a}_{i}}+{{b}_{i}}{{P}_{Gi}}+{{c}_{i}}{{P}_{Gi}}^{2}+\left| {{d}_{i}}\times \sin \left( {{e}_{i}}\times \left( P_{Gi}^{\min }-{{P}_{Gi}} \right) \right) \right| } \end{aligned}$$

where ${{a}_{i}},{{b}_{i}},{{c}_{i}},{{d}_{i}},{{e}_{i}}$: coefficients of cost of the ${{i}^{th}}$ generator; ${{P}_{Gi}}$: active power generation of ${{i}^{th}}$ generator; $P_{Gi}^{\min }$: minimum active power generation limit of ${{i}^{th}}$ generator. Here *NG*: Number of thermal energy units.

46 $$\begin{aligned} {\left\{ \begin{array}{ll} & {{Cost}_{wind}}={\sum \limits _{m=1}^{{{N}_{wind}}}{{Cost}_{windm}}\left( {{P}_{windm}}\right) } \\ & = {\sum \limits _{m=1}^{{{N}_{wind}}}{\left( {{Cost}^{O}_{windm}}+{{Cost}^{U}_{windm}} \right) }} \\ \end{array}\right. } \end{aligned}$$

where ${{Cost}_{wind}}$ denotes the wind cost and ${{N}_{wind}}$ represent the total no of wind units.

47 $$\begin{aligned} {{Cost}_{solarl}}={{Cost}^{d}_{solarl}}+{{Cost}^{O}_{solarl}}+{{Cost}^{U}_{solarl}} \end{aligned}$$

Here direct, overestimation and underestimation cost are denoted with ${{Cost}^{d}_{solarl}}$, ${{Cost}^{O}_{solarl}}$ and ${{Cost}^{U}_{solarl}}$ of the ${l^{th}}$ solar unit.

The total generation cost is

48 $$\begin{aligned} F=\min \left[ FC+ {{Cost}_{wind}}+ {{Cost}_{solarl}}+\sum \limits _{h=24}{\sum \limits _{i=1}{\left( C_{HDi}^{h}P_{HDi}^{h}-C_{HCi}^{h}P_{HCi}^{h} \right) }} \right] \end{aligned}$$

#### Emission

When fossil fuels are used to generate electricity, toxic gases are released into the atmosphere. The emissions coming from thermal generators can be shown as follows:

49 $$\begin{aligned} {\left\{ \begin{array}{ll} Min{{\mathcal {F}}_{2}}=\sum \limits _{k=1}^{{{N}_{TG}}}{{{10}^{-2}}\left( {{\chi }_{k}}+{{\eta }_{k}}{{P}_{TGk}}+{{\sigma }_{k}}P_{TGk}^{2} \right) }\\ +\left( {{\omega }_{k}}{{e}^{\left( {{\mu }_{k}}{{P}_{TGk}} \right) }} \right) \\ \end{array}\right. } \end{aligned}$$

Here ${{\chi }_{k}},{{\eta }_{k}},{{\sigma }_{k}},{{\omega }_{k}}\, \& \,{{\mu }_{k}}$ are coefficients of emission of the ${{k}^{th}}$ generator.${{P}_{TGk}}$ is the power generation by ${{k}^{th}}$ generator.

#### Frequency deviation

To successfully dampen down the tie-line power and frequency oscillation, the control parameter must be determined using an appropriate objective function. The performance of the system is also impacted by the selection of an appropriate objective function. In this case, the objective function can be described as follows:

50 $$\begin{aligned} \Delta {f}=\displaystyle \sum \limits _{i=1}^{NG}{ \Delta {{f_1}^{2}}+\Delta {{f_2}^{2}}+\Delta {{f_3}^{2}}+\Delta P_{tie_{12}}^{2}+\Delta P_{{tie}_{23}}^{2}+\Delta P_{{tie}_{31}}^{2} } \end{aligned}$$

#### Multi-objective

Finding the best solution that simultaneously minimizes various objectives. These optimization issues are known as multi-objective optimization problems.

Simultaneous reduction of costs and emissions: The function is expressed as:

51 $$\begin{aligned} O{{F}_{comb1}}={{FC}+{{\alpha }_{EC}}{F}}_{2} \end{aligned}$$

${{\alpha }_{EC}}$ indicates the emission-related weighting factor.

### Constraints

The OPF has the following restrictions.

#### Equality constraints

These limitations provide the following expression for the load flow equations:

52 $$\begin{aligned} & \left\{ \begin{array}{c} \sum \limits _{i=1}^{NB}{\left( {{P}_{Gi}}-{{P}_{Li}} \right) }=\sum \limits _{i=1}^{NB}{{}}\sum \limits _{j=1}^{NB}{\left| {{V}_{i}} \right| \left| {{V}_{j}} \right| \left| {{Y}_{ij}} \right| \operatorname {Cos}\left( {{\theta }_{ij}}-{{\delta }_{ij}} \right) } \\ \\ \sum \limits _{i=1}^{NB}{{}}{{Q}_{Gi}}-{{Q}_{Li}}=-\sum \limits _{i=1}^{NB}{{}}\sum \limits _{j=1}^{NB}{\left| {{V}_{i}} \right| \left| {{V}_{j}} \right| \left| {{Y}_{ij}} \right| \operatorname {Sin}\left( {{\theta }_{ij}}-{{\delta }_{ij}} \right) } \\ \end{array} \right. \end{aligned}$$

53 $$\begin{aligned} & \left\{ \begin{array}{l} \sum \limits _{i=1}^{NB}{\left( {{P}_{Gi}}-{{P}_{Li}} \right) }+\sum \limits _{i=1}^{NTCSC} {{P}_{is}}+\sum \limits _{i=1}^{NTCPS} {{P}_{is}} =\sum \limits _{i=1}^{NB}{{}}\sum \limits _{j=1}^{NB}{\left| {{V}_{i}} \right| \left| {{V}_{j}} \right| \left| {{Y}_{ij}} \right| \operatorname {Cos}\left( {{\theta }_{ij}}-{{\delta }_{ij}} \right) } \\ \\ \sum \limits _{i=1}^{NB}{\left( {{Q}_{Gi}}-{{Q}_{Li}}\right) }+\sum \limits _{i=1}^{NTCSC} {{Q}_{is}}+\sum \limits _{i=1}^{NTCPS} {{Q}_{is}}=-\sum \limits _{i=1}^{NB}{{}}\sum \limits _{j=1}^{NB}{\left| {{V}_{i}} \right| \left| {{V}_{j}} \right| \left| {{Y}_{ij}} \right| \operatorname {Sin}\left( {{\theta }_{ij}}-{{\delta }_{ij}} \right) } \\ \end{array} \right. \end{aligned}$$

${{P}_{Gi}}$,${{P}_{Li}}$ : active generating power and demand respectively of ${{i}^{th}}$ bus; ${{Q}_{Gi}},{{Q}_{Li}}$ : reactive generating power and demand respectively of ${{i}^{th}}$ bus; ${{Y}_{ij}}$: admittance of line connected between ${{i}^{th}}$ and ${{j}^{th}}$ bus; ${{\theta }_{ij}}$ : admittance angle of line connected between ${{i}^{th}}$ and ${{j}^{th}}$ bus; *NB* : no. of buses.

#### Inequality constraints

(i) Generator constraints:

54 $$\begin{aligned} \left\{ \begin{array}{l} V_{Gi}^{\min }\le {{V}_{Gi}}\le V_{Gi}^{\max } \\ P_{Gi}^{\min }\le {{P}_{Gi}}\le P_{Gi}^{\max } \\ Q_{Gi}^{\min }\le {{Q}_{Gi}}\le Q_{Gi}^{\max } \end{array} \right. {} \quad i\in NG \quad {} \end{aligned}$$

(ii) Load bus constraints:

55 $$\begin{aligned} V_{Li}^{\min }\le {{V}_{Li}}\le V_{Li}^{\max }\begin{matrix} {} & i\in NL \\ \end{matrix} \end{aligned}$$

(iii) Transmission line constraints:

56 $$\begin{aligned} & {{S}_{L}}_{i}\le S_{Li}^{\max }\begin{matrix} {} & i\in NTL \\ \end{matrix} \end{aligned}$$

(iv) Transformer tap constraints:

57 $$\begin{aligned} & T_{i}^{\min }\le {{T}_{i}}\le T_{i}^{\max }\begin{matrix} {} & i\in NT \\ \end{matrix} \end{aligned}$$

(v) Shunt compensator constraints:

58 $$\begin{aligned} Q_{Ci}^{\min }\le {{Q}_{Ci}}\le Q_{Ci}^{\max }\begin{matrix} {} & i\in NC \\ \end{matrix} \end{aligned}$$

(vi) Wind power constraints:

59 $$\begin{aligned} P_{windi}^{\min }\le {{P}_{windi}}\le P_{windi}^{\max } where, i=1,2,3,...,{{N}_{wind}} \end{aligned}$$

(vii) Solar power constraints:

60 $$\begin{aligned} P_{Solari}^{\min }\le {{P}_{Solari}}\le P_{Solari}^{\max } where, i=1,2,3,...,{{N}_{Solar}} \end{aligned}$$

(viii) Frequency security constraint: After determining the maximum frequency deviation equation, the frequency security limitation is developed based on the requirement to maintain the frequency deviation within specific safe bounds:

61 $$\begin{aligned} -\Delta {{f}_{s}}\le \Delta {{f}_{\max }}\le \Delta {{f}_{s}} \end{aligned}$$

where

62 $$\begin{aligned} \Delta {{f}_{\max }}=\frac{\Delta {{P}_{L}}}{{{R}_{R}}+D}\left( 1+{{e}^{\varsigma {{\omega }_{n}}{{t}_{\max }}}}\sqrt{\frac{{{T}_{R}}\left( {{R}_{R}}-{{F}_{R}} \right) }{2H}} \right) \end{aligned}$$

The maximum frequency deviation will change in response to changes in the inertia of the generators. where $V_{Gi}^{\min },V_{Gi}^{\max }$: smallest and greatest voltage limits respectively of ${{i}^{th}}$ generator bus; $P_{Gi}^{\min }$, $V_{Li}^{\min }$,$V_{Li}^{\max }$: smallest and greatest voltage limits respectively of ${{i}^{th}}$ load bus; ${{S}_{Li}},S_{Li}^{\max }$: apparent power flow and maximum apparent power flow limit respectively of ${{i}^{th}}$ branch;$T_{i}^{\min },T_{i}^{\max }$: smallest and greatest tap setting limits respectively of ${{i}^{th}}$ regulating transformer; *NG*: no. of generator buses; *NL*: no. of load buses; *NTL*: no. of transmission line; *NT*: no. of regulating transformers; *NC*: no. of shunt compensator. There are shown the minimum and maximum solar power limits for ${{i}^{th}}$ units and ${{i}^{th}}$ co-generation units are ${P_{poui}^{\min }}$, ${P_{poui}^{\max }}$; $P_{windi}^{\min }$ is the minimum power production of ${{i}^{th}}$ wind $P_{windi}^{\max }$ is shown maximum power production of ${{i}^{th}}$ wind.

(ix) Ultra capacitor (UC) state of charge constraint: The power consumed for charging (or discharging) during this session and the charge level at the end of the previous one are added to calculate the UC state of charge (SOC) at the end of each time interval. Because of UC’s effectiveness when charging and discharging, we can describe the state of charging limitations as

63 $$\begin{aligned} E_{i}^{t}=E_{i}^{t-1}+\left( P_{BCi}^{t}\times {{\eta }_{ci}}-\frac{P_{BDi}^{t}}{{{\eta }_{di}}} \right) \times \Delta t \end{aligned}$$

${E_{i}^{t}}$: Stored energy in the UC at time *t*, at bus *i*. ${P_{BCi}^{t}}$: I/P power for UC charging at bus *i* during time *t*. ${P_{BDi}^{t}}$: UC O/P power at bus *i* is being discharged during time *t*. ${{\eta }_{ci}}$, ${{\eta }_{di}}$: UC energy storage efficiency during charging and discharging at bus *i* respectively. $\Delta t$: time interval.

(x) UC capacity constraints:

64 $$\begin{aligned} & 0\le E_{i}^{t}\le \overline{{{E}_{i}}} \end{aligned}$$

65 $$\begin{aligned} & E_{i}^{T}=E_{i}^{fin} \end{aligned}$$

66 $$\begin{aligned} & E_{i}^{0}=E_{i}^{ini} \end{aligned}$$

${E_{i}^{t}}$: Stored energy in the UC at time *t*, at bus *i*. ${\overline{{{E}_{i}}}}$: Highest energy storage capacity at bus *i*. ${E_{i}^{fin}}$: Final energy conserved in the battery at bus *i*. ${E_{i}^{ini}}$: Initially conserved energy at bus *i* in UC.

(xi) UC charging and discharging power constraints:

67 $$\begin{aligned} & uc_{i}^{t}+ud_{i}^{t}\le 1 \end{aligned}$$

68 $$\begin{aligned} & 0\le P_{BCi}^{t}\le uc_{i}^{t}\cdot \overline{{{P}_{BCi}}} \end{aligned}$$

69 $$\begin{aligned} & 0\le P_{BDi}^{t}\le ud_{i}^{t}\cdot \overline{{{P}_{BDi}}} \end{aligned}$$

${uc_{i}^{t}}$, ${ud_{i}^{t}}$: binary variable representing the UC charging and discharging status, respectively, at bus *i* during period *t*. 1-discharging, 0-not discharging.

## Algorithm for optimization

### DTBO

DTBO was first introduced by Dehghani et al.^30,42^. DTBO is a technique that is based on populations. The DTBO deployment provides how a driving teacher might teach students at a driving school. There are three stages in the DTBO mathematical framework: (1) Driving instructor’s instructions (2) Student patterning depends upon instructor ability and (3) perform. The degree of thinking a person possesses finds how well they can learn and become excellent drivers. In a driving school, a skilled driver may receive instruction from a number of teachers. New drivers enhance their driving abilities by practicing alone and following their instructor’s directions. These interactions between students and teachers as well as self-practice to improve their driving abilities form the basis of the mathematical modeling of DTBO. The DTBO population matrix is shown below:

70 $$\begin{aligned} Z={{\left[ {\left\{ \begin{array}{ll} & {{Z}_{1}} \\ & \\ & \\ & {{Z}_{p}} \\ & \\ & \\ & {{Z}_{N}} \\ \end{array}\right. } \right] }_{N\times m}}={{\left[ {\left\{ \begin{array}{ll} & {{z}_{11}}\quad {{z}_{1q}}\quad {{z}_{1m}} \\ & \quad \quad \quad \quad \quad \quad \quad \\ & \quad \quad \quad \quad \quad \quad \quad \\ & {{z}_{p1}}\quad \quad \quad {{z}_{pq}}\quad \quad {{z}_{pm}}\ \\ & \quad \quad \quad \quad \quad \quad \quad \\ & \quad \quad \quad \quad \quad \quad \quad \\ & {{z}_{N1}}\ \ \quad \quad {{z}_{Nq}}\quad \quad {{z}_{Nm}} \\ \end{array}\right. } \right] }_{N\times m}} \end{aligned}$$

where Z: the DTBO population; ${{Z}_{p}}$: ${{p}^{th}}$ member of Z; ${{z}_{pq}}$: the ${{q}^{th}}$ variable of the ${{p}^{th}}$ solution of the problem; *N*: the population size. The following methods are used to arbitrarily initialize the starting positions of DTBO members:

71 $$\begin{aligned} {{z}_{pq}}=z_{pq}^{\min }+r*\left( z_{pq}^{\max }-z_{pq}^{\min } \right) \quad for\,p=1\,to\,N\ { \& } \ q=1\ to\ m \end{aligned}$$

where $z_{pq}^{\max }$ and $z_{pq}^{\min }$: higher and lower limits for the ${{q}^{th}}$ variable respectively; *r*: random number between 0 and 1. For every distinct candidate solution, the value of the objective function can be calculated and expressed as follows:

72 $$\begin{aligned} F={{\left[ {\left\{ \begin{array}{ll} & {{F}_{1}} \\ & . \\ & . \\ & {{F}_{p}} \\ & . \\ & . \\ & {{F}_{N}} \\ \end{array}\right. } \right] }_{N\times 1}}={{\left[ {\left\{ \begin{array}{ll} & F\left( {{Z}_{1}} \right) \\ & \\ & \\ & F\left( {{Z}_{p}} \right) \\ & \\ & \\ & F\left( {{Z}_{N}} \right) \\ \end{array}\right. } \right] }_{N\times 1}} \end{aligned}$$

The candidate solution that achieves the highest value of the objective function is selected as the best member. As the iteration increases, the top member obtains updates. The DTBO candidate solution updating method consists of three parts: 

Step 1: Instruction from the driving instructor (Exploration): Only a small percentage of the DTBO population is selected to be a driving instructor; the rest are classified as trainee drivers. By carefully choosing instructors to develop their skills, it is possible to conduct a worldwide search to find the best solution area for the given problem. In each iteration, *L* DTBO members are identified as teachers by comparing the values of the objective function. The driving matrix *DI* is represented by these members in the way that follows:

73 $$\begin{aligned} DI={{\left[ {\left\{ \begin{array}{ll} & D{{I}_{1}} \\ & \\ & \\ & D{{I}_{p}} \\ & \\ & D{{I}_{L}} \\ \end{array}\right. } \right] }_{L\times m}}={{\left[ {\left\{ \begin{array}{ll} & D{{I}_{11}}\quad \quad \quad D{{I}_{1q}}\quad \quad D{{I}_{1m}} \\ & \quad \quad \quad \quad \quad \quad \ \ \ \ \quad \\ & \quad \quad \quad \quad \quad \quad \ \ \ \quad \\ & D{{I}_{p1}}\quad \quad \quad D{{I}_{pq}}\quad \quad D{{I}_{pm}}\ \\ & \quad \quad \quad \quad \quad \quad \ \ \ \quad \\ & \quad \quad \quad \quad \quad \quad \ \ \ \quad \\ & D{{I}_{L1}}\ \ \quad \quad D{{I}_{Lq}}\quad \quad D{{I}_{Lm}} \\ \end{array}\right. } \right] }_{L\times m}} \end{aligned}$$

$D{{I}_{p}}$ is ${p}^{th}$ driving instructor. $D{{I}_{pq}}$ is ${{q}^{th}}$ variable of ${p}^{th}$ instructor.

74 $$\begin{aligned} L=\left\lfloor 0.1\times N\times \left( \frac{1-s}{S} \right) \right\rfloor \end{aligned}$$

The DTBO population member’s adjusted position is calculated as follows:

75 $$\begin{aligned} z_{pq}^{st1}={\left\{ \begin{array}{ll} & {{z}_{pq}}+r.\left( D{{I}_{kpq}}-I.{{z}_{pq}} \right) ,\ \ {{F}_{DI{{k}_{p}}}}<{{F}_{p}} \\ & {{z}_{pq}}+r.\left( {{z}_{pq}}-D{{I}_{kpq}} \right) ,\ otherwise \\ \end{array}\right. } \end{aligned}$$

*I* and *r* are random numbers and random values between 0 and 1 in the set $\{1,2\}$. The collection 1,2,...,L is used to randomly select *k* in $D{{I}_{kpq}}$ *i*.*e*. ${{k}^{th}}$ driving instructor with the objective function value ${{F}_{DI{{k}_{p}}}}$, *p* indicates ${{p}^{th}}$ trainee member of the population who is being trained by the ${{k}^{th}}$ teacher. The new position position is changed by (76).

76 $$\begin{aligned} {{Z}_{p}}= {\left\{ \begin{array}{ll} & Z_{p}^{st1},\quad F_{p}^{st1}<{{F}_{p}} \\ & {{Z}_{p}},\quad otherwise \\ \end{array}\right. } \end{aligned}$$

The revised ${{p}^{th}}$ candidate solution at the $1^{st}$ DTBO step is $Z_{p}^{st1}$ ; $z_{pq}^{st1}$ is its ${{q}^{th}}$ problem variable, the value of its objective function is $F_{p}^{st1}$.

Step 2: Patterning of the instructor skills of the student driver (Exploration): The trainee driver improves the DTBO solutions in the $2^{nd}$ phase by adopting the instructor’s methods and manners. Through this process, members of the DTBO are arriving at a new region of the search area. It augments DTBO’s capability for exploration. The modified position, articulated mathematically by (77), is formed by a linear arrangement of DTBO members and instructors. If the target function’s value is higher at the new location than at the old one, (78) replaces the previous position (Tables 3, 4).

**Table 3:** OPF solution for IEEE 57 bus test system for six load scenarios without frequency security limitation (Case 1).

	Control								
	Parameters	Min.	Max.	50%	60%	70%	80%	90%	100%
	PTG1	0	575.88	336.76	422.8	406.33	428.26	527.02	563.28
	PTG2	0	100	82.78	83.49	80.03	96.29	98.81	98.78
	PTG3	0	140	0	0	33.94	67.02	72.71	79.07
	PTG6	0	100	99.07	97.54	95.7	99.8	99.49	98.3
	PTG8	0	550	0	0	40.15	56.15	49.72	93.85
	PTG9	0	100	80.18	86.9	88.74	88.36	96.18	98.21
Power (MW)	PTG12	0	410	69.14	103.44	170.55	201.97	225.52	261.84
	$${V}_{1}$$	0.95	1.1	1.0407	1.0594	1.0325	1.0556	1.0588	1.0574
	$${V}_{2}$$	0.95	1.1	1.04	1.0565	1.0212	1.0554	1.0569	1.0559
	$${V}_{3}$$	0.95	1.1	1.0242	1.0247	1.0012	1.0406	1.0373	1.0258
	$${V}_{6}$$	0.95	1.1	1.0122	1.0088	0.9656	1.03	1.0015	1.0191
	$${V}_{8}$$	0.95	1.1	1.0085	1.0061	0.9737	1.0184	0.9827	1.0271
	$${V}_{9}$$	0.95	1.1	1.0096	1.0144	0.9672	1.0194	0.9975	1.0253
Voltage (p.u.)	$${V}_{12}$$	0.95	1.1	1.009	1.0136	0.9734	1.0254	1.0169	1.0227
	$${T}_{19}$$(4-18)	0.9	1.1	1.0222	0.9	0.9199	1.0127	0.9	0.9283
	$${T}_{20}$$(4-18)	0.9	1.1	0.979	0.9513	0.9679	1.0163	0.9	1.0386
	$${T}_{31}$$(21-20)	0.9	1.1	0.9993	1.0026	0.9023	1.0311	0.946	1.0971
	$${T}_{35}$$(24-25)	0.9	1.1	0.9048	0.9806	0.9509	0.9687	0.9	0.9259
	$${T}_{36}$$(24-25)	0.9	1.1	1.0136	1.0138	0.9995	0.945	0.9479	1.025
	$${T}_{37}$$(24-26)	0.9	1.1	0.9549	1.0539	1.0194	0.9898	0.9737	1.0436
	$${T}_{41}$$(7-29)	0.9	1.1	0.9707	0.9815	1.0389	0.9798	0.9422	0.9606
	$${T}_{46}$$(34-32)	0.9	1.1	0.9346	0.9787	0.9612	0.9416	0.9144	0.9625
	$${T}_{54}$$(11-41)	0.9	1.1	1.0235	0.948	0.9955	0.9403	0.9381	0.9
	$${T}_{58}$$(15-45)	0.9	1.1	0.9993	0.9262	1.0521	0.9761	0.9371	0.9
	$${T}_{59}$$(14-46)	0.9	1.1	0.9854	0.9254	1.0475	0.9768	0.9436	0.9
	$${T}_{65}$$(10-51)	0.9	1.1	0.9972	0.9185	1.029	0.9954	0.9477	0.9277
	$${T}_{66}$$(13-49)	0.9	1.1	0.9657	0.9	0.9535	0.9628	0.912	0.9
	$${T}_{71}$$(11-43)	0.9	1.1	0.9732	0.9004	0.9829	0.9759	0.9358	0.9
Tap setting (p.u.)	$${T}_{73}$$(40-56)	0.9	1.1	1.0307	1.0005	0.9717	0.9992	1.0498	0.9662
	$${Q}_{C18}$$	0	5	2.73	4.56	4.55	4.21	4.54	3.72
	$${Q}_{C25}$$	0	5	4.92	4.87	4.46	4.82	4.54	5
(MVAr)	$${Q}_{C53}$$	0	5	3.98	4.99	3.84	4.6	4.61	3.55
Total generation				667.9338	794.1683	915.4393	1037.8424	1169.4538	1293.3167
**Total generation cost ($/h)**				**2140.3597**	**2764.9601**	**3350.5598**	**4026.3726**	**4796.6767**	**5774.916**
Emission((t/h)				0.812	1.1976	1.1291	1.271	1.8596	2.1513
$${P}_{loss}$$(MW)				15.03	21.69	23.38	26.2	38.23	42.52
VD(p.u.)				0.8759	2.2784	3.7782	0.8823	1.7078	2.5193
Frequency deviation with load									
	% max load			OS		SS (sec)			
	100			0.01011		17.2			
	90			0.0101		11.08			
	80			0.009885		8.33			
	70			0.009837		8.7			
	60			0.009896		7.6			
Frequency deviation	50			0.01004		6.3

77 $$\begin{aligned} & z_{pq}^{st2}={{z}_{pq}}.\xi +\left( 1-\xi \right) .D{{I}_{kpq}} \end{aligned}$$

78 $$\begin{aligned} & {{Z}_{p}}={\left\{ \begin{array}{ll} & Z_{p}^{st2}\,\ F_{p}^{st2}<{{F}_{p}} \\ & {{Z}_{p}},\ \ otherwise \\ \end{array}\right. } \end{aligned}$$

In the DTBO second stage, the updated ${{p}^{th}}$ candidate solution is $Z_{p}^{st2}$, with $z_{pq}^{st2}$ denoting its ${{q}^{th}}$ variable. The correlated objective function value is $F_{p}^{st2}$. $\xi$, the patterning index, can be obtained by:

79 $$\begin{aligned} \xi =0.01+0.9\left( 1-\frac{s}{S} \right) \end{aligned}$$

Step 3: Personal practice (Exploitation): During this stage, the less-experienced drivers’ driving skills are enhanced by individual practice. It is comparable to consuming the local search feature provided by DTBO. Every student searching for a position that is better than their present position. New positions are created in the immediate vicinity to the current position by (80). Using (81), the new location has been substituted for the old one, and the objective function value is modified as follows:
  80 $$\begin{aligned} & z_{p,q}^{st3}={{z}_{pq}}+\left( 1-2r \right) .R.\left( 1-\frac{s}{S} \right) .{{z}_{pq}} \end{aligned}$$
  81 $$\begin{aligned} & {{Z}_{p}}={\left\{ \begin{array}{ll} & Z_{p}^{st3},\ F_{p}^{st3}<{{F}_{p}} \\ & {{Z}_{p}},\ otherwise \\ \end{array}\right. } \end{aligned}$$

**Table 4:** OPF solution for six load scenarios with frequency security constraints for the IEEE 57 bus test system (Case 5).

	Control								
	Parameters	Min.	Max.	50%	60%	70%	80%	90%	100%
	PTG1	100	575.88	256.13	322.29	406.65	451.83	556.04	555.17
	PTG2	30	100	65.56	46.15	90.45	84.44	97.05	95.67
	PTG3	30	140	41.23	54.46	82.16	71.84	74.97	67.47
	PTG6	30	100	70.42	97.32	94.68	99.76	99.89	93.07
	PTG8	30	550	49.83	57.34	33.02	45.8	48.82	74.19
	PTG9	30	100	47.64	42.36	77.09	95.34	85.57	96.98
Power (MW)	PTG12	100	410	111.44	155.05	137.79	192.48	210.29	311.66
	$${V}_{1}$$	0.95	1.1	1.0332	0.9781	1.0329	1.0195	1.0598	1.0451
	$${V}_{2}$$	0.95	1.1	1.0286	1.0114	1.0531	1.0055	1.0574	1.0234
	$${V}_{3}$$	0.95	1.1	1.0552	1.0517	1.0029	1	1.0422	1.0004
	$${V}_{6}$$	0.95	1.1	0.9982	1.0336	0.9719	0.983	1.0078	0.9674
	$${V}_{8}$$	0.95	1.1	0.973	0.9697	0.9602	0.9717	0.9931	0.95
	$${V}_{9}$$	0.95	1.1	0.9897	1.0094	0.9516	0.9757	1.0044	0.9602
Voltage (p.u.)	$${V}_{12}$$	0.95	1.1	1.0287	0.9532	1.0051	0.9806	1.0286	0.9919
	$${T}_{19}$$(4-18)	0.9	1.1	0.9207	0.95	1.0268	0.9	0.9687	0.9
	$${T}_{20}$$(4-18)	0.9	1.1	1.0131	0.9178	1.0418	1.005	0.9303	0.9
	$${T}_{31}$$(21-20)	0.9	1.1	1.031	0.9685	1.0297	1.0501	1.0576	0.9903
	$${T}_{35}$$(24-25)	0.9	1.1	1.0231	0.9073	1.0716	0.9639	0.9173	0.9
	$${T}_{36}$$(24-25)	0.9	1.1	1.0746	0.9761	1.0805	0.9653	1.0205	0.9267
	$${T}_{37}$$(24-26)	0.9	1.1	0.9704	0.9895	0.9523	1.0545	1.0788	1.0902
	$${T}_{41}$$(7-29)	0.9	1.1	0.994	1.0569	1.0972	0.9747	0.964	0.9685
	$${T}_{46}$$(34-32)	0.9	1.1	1.0324	0.9639	0.9003	0.9273	0.9382	0.9
	$${T}_{54}$$(11-41)	0.9	1.1	1.0061	1.0828	0.9752	0.9	0.9	0.9
	$${T}_{58}$$(15-45)	0.9	1.1	1.0455	1.054	0.9736	0.9	0.9	0.9
	$${T}_{59}$$(14-46)	0.9	1.1	1.042	0.9771	1.0565	0.9	0.9	0.9
	$${T}_{65}$$(10-51)	0.9	1.1	0.9634	1.0206	1.0605	0.926	0.9123	0.9165
	$${T}_{66}$$(13-49)	0.9	1.1	1.0342	0.9677	0.9742	0.9	0.9	0.9
	$${T}_{71}$$(11-43)	0.9	1.1	0.9159	1.04	0.9906	0.9193	0.9	0.9117
Tap setting (p.u)	$${T}_{73}$$(40-56)	0.9	1.1	1.0486	0.9981	1.0291	1.0301	1.0251	1.0239
	$${Q}_{C18}$$	0	5	2.92	0.978	4.92	4.78	4.26	5
	$${Q}_{C25}$$	0	5	0.18	1.83	4.96	4.08	4.98	4.29
(MVAr)	$${Q}_{C53}$$	0	5	1.64	2.32	2.31	4.92	4.92	4.29
Total generation				642.2485	774.9696	921.8487	1041.4705	1172.6292	1294.1966
Total generation cost ($/h)				2145.9374	2771.232	3372.946	4036.9355	4804.2003	5781.8242
Emission((t/h)				0.5078	0.756	1.111	1.3821	2.0264	2.1632
$${P}_{loss}$$(MW)				15.73	24.13	29.79	29.83	41.41	43.4
VD(p.u.)				3.3328	1.9037	4.4353	1.5355	2.8995	2.1602
Frequency deviation with load									
	% max load			OS		SS (sec)			
	100			0.009317		13.28			
	90			0.009714		10.04			
	80			0.009477		5.48			
	70			0.009651		5.21			
	60			0.009529		5.45			
Frequency deviation	50			0.0098		5.01

**Table 5:** OPF solution with TCSC, TCPS, SVC, and the frequency security constraint for the IEEE 57 bus test system with varied load scenarios (Case 9).

	Control								
	Parameters	Min.	Max.	50%	60%	70%	80%	90%	100%
	PTG1	100	575.88	258.47	301.15	421.3	432.63	517.83	518.54
	PTG2	30	100	43	95.5	84.19	83.02	98.16	99.31
	PTG3	30	140	34.98	53.24	35.07	58.42	75.14	91.55
	PTG6	30	100	85.81	90.62	94.31	98.45	100	99.74
	PTG8	30	550	46.59	33.71	41.47	33.39	32.85	44.32
	PTG9	30	100	70	65.41	80.84	100	96	99.27
Power (MW)	PTG12	100	410	101.72	130.78	148.21	214.81	244.67	338.03
	$${V}_{1}$$	0.95	1.1	1.0468	1.0186	1.0447	1.0516	1.06	1.0524
	$${V}_{2}$$	0.95	1.1	1.0133	1.0118	1.0338	1.0519	1.0591	1.0567
	$${V}_{3}$$	0.95	1.1	1.0442	1.0292	1.0388	1.0431	1.0435	1.044
	$${V}_{6}$$	0.95	1.1	0.9859	1.0136	1.0035	1.0006	1.0116	1.0246
	$${V}_{8}$$	0.95	1.1	0.9854	0.9588	0.95	0.967	0.9937	1.02
	$${V}_{9}$$	0.95	1.1	1.0374	0.9828	1.0016	0.9847	0.9985	1.014
Voltage (p.u.)	$${V}_{12}$$	0.95	1.1	1.0054	0.9926	0.9674	1.0032	1.0109	1.0197
	$${T}_{19}$$(4-18)	0.9	1.1	1.0358	0.9749	0.9871	0.9047	0.9406	1.0358
	$${T}_{20}$$(4-18)	0.9	1.1	1.0362	0.9987	1.0865	0.987	0.9533	1.0034
	$${T}_{31}$$(21-20)	0.9	1.1	1.0327	0.9077	1.0735	0.9065	0.9558	1.0625
	$${T}_{35}$$(24-25)	0.9	1.1	1.0685	1.0101	0.9194	1.0292	0.9345	0.9886
	$${T}_{36}$$(24-25)	0.9	1.1	1.0108	0.9365	1.0017	1.0226	0.9048	0.9586
	$${T}_{37}$$(24-26)	0.9	1.1	1.0449	0.9322	1.0885	1.0568	1.0446	1.077
	$${T}_{41}$$(7-29)	0.9	1.1	0.9354	0.902	1.0238	0.9769	1.0179	0.9661
	$${T}_{46}$$(34-32)	0.9	1.1	0.9596	1.0759	1.0805	1.0304	0.9	1.068
	$${T}_{54}$$(11-41)	0.9	1.1	1.0422	0.9176	0.9414	1.0168	0.9544	0.9048
	$${T}_{58}$$(15-45)	0.9	1.1	0.9488	0.9724	1.028	0.9928	0.9869	0.906
	$${T}_{59}$$(14-46)	0.9	1.1	0.993	0.9603	0.9776	1.0235	0.9952	0.9108
	$${T}_{65}$$(10-51)	0.9	1.1	1.0204	0.9991	1.0395	1.013	0.9485	0.9125
	$${T}_{66}$$(13-49)	0.9	1.1	1.0812	0.9815	1.0635	0.989	0.9899	0.9009
	$${T}_{71}$$(11-43)	0.9	1.1	1.0555	1.0528	1.0049	0.9423	0.9626	0.9478
Tap setting (p.u)	$${T}_{73}$$(40-56)(p.u)	0.9	1.1	0.9762	0.9998	1.0793	1.0448	0.9982	0.9741
	$${Q}_{C18}$$	0	5	0.677	0.82	0.665	0.444	0.605	0.665
	$${Q}_{C25}$$	0	5	1.4	3.67	0.09	2.04	0.555	4.61
(MVAr)	$${Q}_{C53}$$	0	5	3.09	0.93	0.59	3.42	0.581	4.45
FACTs rating									
$${{\tau }_{TCSC1}}\%$$		0	50	30.198	27.673	28.871	44.765	30.9875	34.8701
$${{\varphi }_{TCPS1}}$$ (deg)		− 5	5	− 2.245	2.134	− 2.786	4.009	3.998	− 3.654
$${{Q}_{SVC1}}$$ (MVAR)		− 10	10	9.654	9.987	9.456	10	9.0986	9.998
FACTs location									
TCSC1				27–28	24–25	34–35	27–28	37–39	34–35
TCPS1				24–26	24–25	28–29	24–26	25–30	24–25
SVC1				7	21	27	30	24	44
Total generation				640.5621	770.4196	905.3974	1029.5058	1164.6558	1290.7729
Total generation cost ($/h)				2085.0473	2661.9802	3290.7677	3986.2751	4775.7216	5752.0187
Emission				0.519	0.6745	1.1826	1.3514	1.8288	1.9896
$${P}_{loss}$$(MW)				15.1	19.74	29.78	28.82	38.84	39.91
VD(p.u)				1.2722	1.8238	2.984	2.3096	1.4227	2.6534
Frequency deviation with load									
	% max load			OS		SS (s)			
	100			0.00931		13.19			
	90			0.009708		10.01			
	80			0.009468		5.39			
	70			0.009649		5.18			
	60			0.009527		5.39			
Frequency deviation	50			0.00909		4.98

**Table 6:** OPF result without frequency security constraints for six load situations in the IEEE 57 bus test system (Case 2).

	Control								
	Parameters	Min.	Max.	50%	60%	70%	80%	90%	100%
	PTG1	0	575.88	103.73	129.68	129.55	165.9	166.27	201.15
	PTG2	0	100	0	0	0	98.12	94.5	95.26
	PTG3	0	140	137.34	133.83	137.09	135	128.34	139.79
	PTG6	0	100	0	0	96.87	96.21	99.8	98.64
	PTG8	0	550	130.58	182.96	189.48	214.46	229.08	279.04
	PTG9	0	100	99.35	92.35	99.41	93.39	89.22	99.16
Power (MW)	PTG12	0	410	158.71	217.03	230.46	210.01	330.59	351.38
	$${V}_{1}$$	0.95	1.1	1.0275	1.0121	0.9565	1.0121	0.9956	1.0362
	$${V}_{2}$$	0.95	1.1	1.0252	1.0132	0.9607	1.0161	0.9912	1.0366
	$${V}_{3}$$	0.95	1.1	1.0213	1.023	0.9811	1.0076	1.0002	1.0387
	$${V}_{6}$$	0.95	1.1	0.9979	1.0196	0.9819	1.0066	1.0129	1.0111
	$${V}_{8}$$	0.95	1.1	1.0091	1.0266	0.978	1.0107	1.0251	1.0249
	$${V}_{9}$$	0.95	1.1	1.011	1.0173	0.9682	0.9942	1.0243	1.0242
Voltage (p.u.)	$${V}_{12}$$	0.95	1.1	1.012	0.9993	0.951	0.9874	1.0067	1.0089
	$${T}_{19}$$(4-18)	0.9	1.1	1.0485	1.015	0.9418	0.9	0.9168	0.9669
	$${T}_{20}$$(4-18)	0.9	1.1	0.9404	1.007	0.9176	1.0055	0.9962	1.0007
	$${T}_{31}$$(21-20)	0.9	1.1	1.0048	0.9886	1.0044	1.0172	0.9846	1.0778
	$${T}_{35}$$(24-25)	0.9	1.1	0.9704	0.9	0.9472	0.9337	0.9944	0.9
	$${T}_{36}$$(24-25)	0.9	1.1	1.0154	0.9471	1.0352	1.0256	0.9452	0.9
	$${T}_{37}$$(24-26)	0.9	1.1	1.0051	0.9138	1.0688	1.0491	1.02	0.9733
	$${T}_{41}$$1(7-29)	0.9	1.1	0.9952	0.9749	0.9855	0.9879	0.9874	0.92
	$${T}_{46}$$(34-32)	0.9	1.1	0.9538	0.9093	0.9443	0.9488	0.9357	0.9
	$${T}_{54}$$(11-41)	0.9	1.1	0.9608	0.9998	0.9	0.9869	0.9	0.9
	$${T}_{58}$$(15-45)	0.9	1.1	1.005	1.0287	0.9023	0.9432	0.9368	0.9227
	$${T}_{59}$$(14-46)	0.9	1.1	0.9906	1.0165	0.9	0.9422	0.9382	0.9267
	$${T}_{65}$$(10-51)	0.9	1.1	1.0151	1.0351	0.9	0.9508	0.9611	0.9354
	$${T}_{66}$$(13-49)	0.9	1.1	0.9693	0.9924	0.9	0.9169	0.9183	0.9
	$${T}_{71}$$(11-43)	0.9	1.1	1.0606	0.9762	0.9356	0.9	0.959	0.9
Tap setting (p.u)	$${T}_{73}$$(40-56)	0.9	1.1	1.058	0.9603	1.0041	1.0609	1.0121	0.9673
	$${Q}_{C18}$$	0	5	0.012	0.015	0.0326	0.0431	0.0418	0.0114
	$${Q}_{C25}$$	0	5	0.0475	0.0499	0.0433	0.0495	0.0465	0.0435
(MVAr)	$${Q}_{C53}$$	0	5	0.0489	0.0486	0.0481	0.0356	0.0448	0.0354
Total generation				629.71	755.8574	882.8495	1013.092	1137.795	1264.4187
Total generation cost ($/h)				3471.0284	4923.1687	5491.2902	6381.8108	7645.4733	9768.2699
Emission (t/h)				0.3574	0.4787	0.512	0.5622	0.7572	0.9657
$${P}_{loss}$$(MW)				4.31	5.38	7.29	12.45	12.07	13.62
VD(p.u)				1.1132	1.7057	1.3669	0.943	0.8472	2.5182
Frequency deviation with load									
	% max load			OS		SS (s)			
	100			0.009457		18.2			
	90			0.009723		10.08			
	80			0.009898		8.33			
	70			0.009867		8.7			
	60			0.009594		7.6			
Frequency deviation	50			0.01007		6.3

**Table 7:** Thermal generators’ costs and emission coefficients for the IEEE 57-bus system.

Generator	Bus	a	b	c	$\alpha$	$\beta$	$\gamma$	$\omega$	$\mu$
TG1	1	0	2	0.00375	4.091	− 5.554	6.49	0.0002	0.286
TG2	2	0	1.75	0.0175	2.543	− 6.047	5.638	0.0005	0.333
TG3	3	0	3	0.025	6.131	− 5.555	5.151	0.00001	0.667
TG6	6	0	2	0.00375	3.491	− 5.754	6.39	0.0002	0.266
TG8	8	0	1	0.0625	4.258	− 5.094	4.586	0.000001	0.800
TG9	9	0	1.75	0.0195	2.754	− 5.847	5.238	0.0004	0.288
TG12	12	0	3.25	0.00834	5.326	− 3.555	3.38	0.002	0.200

**Table 8:** Thermal generator power, voltage limitations and dynamic data for an IEEE 57-bus test system.

Generator	Bus	${{P}_{min}}$	${{P}_{max}}$	${Q}_{min}$	${{Q}_{max}}$	${{V}_{min}}$	*V*	H
TG1	1	100	576	− 140	200	0.95	1.05	30
TG2	2	30	100	− 17	50	0.95	1.05	2.8
TG3	3	30	140	− 10	60	0.95	1.05	4.4
TG6	6	30	100	− 8	25	0.95	1.05	2.8
TG8	8	30	550	− 140	200	0.95	1.05	28
TG9	9	30	100	− 3	9	0.95	1.05	2.8
TG12	12	100	410	− 150	155	0.95	1.05	22

**Table 9:** OPF result for six load scenarios in the IEEE 57 bus test system with frequency security limitations (Case 6).

	Control								
	Parameters	Min.	Max.	50%	60%	70%	80%	90%	100%
	PTG1	100	575.88	100	100	109.96	116.48	183.73	191.53
	PTG2	30	100	70.45	91.43	99.22	96.05	99.72	99.6
	PTG3	30	140	91.16	106.04	130.93	131.79	139.82	139.51
	PTG6	30	100	86.74	97.96	99.47	91.72	96.77	97.48
	PTG8	30	550	90.44	132.75	167.61	212.68	228.62	268.12
	PTG9	30	100	93.45	91.13	92.12	93.43	98.83	99.9
Power (MW)	PTG12	100	410	104.8	140.39	186.18	268.64	290.86	367.09
	$${V}_{1}$$	0.95	1.1	0.9575	0.982	1.052	1.0432	1.0259	1.0433
	$${V}_{2}$$	0.95	1.1	1.0139	0.9888	1.0564	1.0529	1.0252	1.0457
	$${V}_{3}$$	0.95	1.1	1.0288	0.9929	1.0227	1.0395	1.0159	1.0401
	$${V}_{6}$$	0.95	1.1	1.0433	0.9632	1.0058	1.0361	1.0145	1.0274
	$${V}_{8}$$	0.95	1.1	1.0159	0.9581	1.0133	1.0407	1.0133	1.023
	$${V}_{9}$$	0.95	1.1	1.0027	0.9621	1.0011	1.0331	1.0091	1.0208
Voltage (p.u.)	$${V}_{12}$$	0.95	1.1	0.9585	0.9639	1.0025	1.0218	0.9982	1.0149
	$${T}_{19}$$(4-18)	0.9	1.1	1.0016	0.9	1.064	0.9	0.9	0.9
	$${T}_{20}$$(4-18)	0.9	1.1	1.0349	0.9673	0.944	1.0135	0.9352	0.9
	$${T}_{31}$$(21-20)	0.9	1.1	1.0333	0.9069	1.0566	1.0307	0.9519	0.9891
	$${T}_{35}$$(24-25)	0.9	1.1	0.9932	0.9124	0.9361	0.9276	0.9094	0.9081
	$${T}_{36}$$24-25)	0.9	1.1	0.9941	0.9511	0.9914	0.9	0.9	0.9304
	$${T}_{37}$$(24-26)	0.9	1.1	0.9959	0.9568	1.0759	1.0464	1.0298	1.0719
	$${T}_{41}$$(7-29)	0.9	1.1	1.0063	0.9888	1.0028	0.9821	0.9748	0.9651
	$${T}_{46}$$(34-32)	0.9	1.1	1.0622	0.9	0.9288	0.9	0.9	0.9
	$${T}_{54}$$(11-41)	0.9	1.1	0.9843	0.9918	0.9	0.9076	0.9	0.9
	$${T}_{58}$$(15-45)	0.9	1.1	0.929	1.0366	0.9719	0.9242	0.9568	0.9
	$${T}_{59}$$(14-46)	0.9	1.1	0.982	1.0603	0.9411	0.9192	0.9455	0.9
	$${T}_{65}$$(10-51)	0.9	1.1	0.9476	0.9821	0.9582	0.9489	0.9554	0.9065
	$${T}_{66}$$(13-49)	0.9	1.1	1.0013	0.9869	0.9238	0.9	0.9011	0.9
	$${T}_{71}$$(11-43)	0.9	1.1	0.9246	1.0219	0.983	0.9141	0.967	0.9
Tap setting (p.u)	$${T}_{73}$$(40-56)	0.9	1.1	1.015	1.0373	1.0291	1.0016	0.9993	1.0077
	$${Q}_{C18}$$	0	5	0.0308	0.0417	0.0245	0.0405	0.0386	0.032
	$${Q}_{C25}$$	0	5	0.033	0.0465	0.0388	0.0494	0.0473	0.0493
(MVAr)	$${Q}_{C53}$$	0	5	0.045	0.0433	0.0437	0.0493	0.0497	0.05
Total generation				636.8211	759.6943	885.4996	1010.3497	1138.3554	1263.2289
Total generation cost ($/h)				2558.4265	3607.0168	4876.1562	6405.8005	7551.3775	9501.4329
Emission (t/h)				0.2575	0.3158	0.4102	0.5441	0.7307	0.9588
$${P}_{loss}$$(MW)				11.42	9.21	9.94	9.71	12.64	0.1243
VD(p.u)				1.5597	3.7239	1.1059	3.1494	1.3823	3.4511
Frequency deviation with load									
	% max load			OS		SS (sec)			
	100			0.009397		16.47			
	90			0.009715		10.01			
	80			0.009709		8.08			
	70			0.009838		8.62			
	60			0.009490		7.02			
Frequency deviation	50			0.009045		5.80

**Table 10:** OPF result for variable load situations with TCSC, TCPS, and SVC for the IEEE 57 bus test system while taking the frequency security restriction into account (Case 10).

	Control								
	Parameters	Min.	Max.	50%	60%	70%	80%	90%	100%
	PTG1	100	575.88	100	100.02	113.16	128.09	155.77	182.86
	PTG2	30	100	80.8	74.96	97.77	100	100	100
	PTG3	30	140	85.5	96.13	116.58	139.39	136.35	139.5
	PTG6	30	100	75.99	89.99	98.15	100	108.28	100
	PTG8	30	550	103.33	143.67	162.51	180.37	239.87	278.98
	PTG9	30	100	94.58	95.31	99.83	100	100	100
Power (MW)	PTG12	100	410	103.16	156.68	196.88	254.59	296.39	359.21
	$${V}_{1}$$	0.95	1.1	0.9479	1.0119	1.011	1.0104	1.0485	1.026
	$${V}_{2}$$	0.95	1.1	0.9938	1.0179	1.0189	1.0211	1.0482	1.027
	$${V}_{3}$$	0.95	1.1	1.028	1.0145	0.9903	1.0104	1.0449	1.0238
	$${V}_{6}$$	0.95	1.1	1.0252	1.0142	0.9868	0.9932	1.0347	1.0125
	$${V}_{8}$$	0.95	1.1	0.9969	1.0094	1.0002	0.9878	1.0344	1.0139
	$${V}_{9}$$	0.95	1.1	0.9856	1.0028	1.0058	0.984	1.0291	1.0044
Voltage (p.u.)	$${V}_{12}$$	0.95	1.1	1.0269	0.9947	0.9973	0.9854	1.0236	1.0038
	$${T}_{19}$$(4-18)	0.9	1.1	1.0177	0.9028	0.9	0.9	0.9	0.9254
	$${T}_{20}$$(4-18)	0.9	1.1	1.0979	0.9165	0.9137	0.9601	0.9	0.9922
	$${T}_{31}$$(21-20)	0.9	1.1	1.0881	0.9652	1.0194	0.9378	0.9992	0.9678
	$${T}_{35}$$(24-25)	0.9	1.1	1.04	0.9057	0.9081	0.911	0.9	0.9
	$${T}_{36}$$6(24-25)	0.9	1.1	0.9199	0.9139	0.9769	0.909	0.9	0.9
	$${T}_{37}$$(24-26)	0.9	1.1	1.0594	1.0416	1.0887	0.9692	1.0762	0.9663
	$${T}_{41}$$(7-29)	0.9	1.1	0.9205	0.9851	1.0002	0.9672	0.9741	0.9484
	$${T}_{46}$$(34-32)	0.9	1.1	1.0476	0.9	0.9135	0.9118	0.9	0.9
	$${T}_{54}$$(11-41)	0.9	1.1	0.9026	0.987	0.9038	0.9	0.9	1.0642
	$${T}_{58}$$(15-45)	0.9	1.1	0.9226	0.9448	0.9291	1.0043	0.9013	0.9931
	$${T}_{59}$$(14-46)	0.9	1.1	1.0973	0.9431	0.9178	0.9987	0.9	0.9909
	$${T}_{65}$$(10-51)	0.9	1.1	0.9161	0.9483	0.9122	1.0013	0.9021	1.0007
	$${T}_{66}$$(13-49)	0.9	1.1	0.9507	0.9158	0.9019	0.9683	0.9	0.9596
	$${T}_{71}$$(11-43)	0.9	1.1	0.9538	0.9802	0.9	0.9481	0.9	0.9717
Tap setting (p.u)	$${T}_{73}$$(40-56)	0.9	1.1	0.9173	1.0993	1.0275	0.964	1.0068	1.0486
	$${Q}_{C18}$$	0	5	1.24	2.06	3.5	4.24	5	5
	$${Q}_{C25}$$	0	5	4.31	5	4.71	5	5	4.97
(MVAr)	$${Q}_{C53}$$	0	5	1.36	4.97	4	4.75	4.89	5
FACTs Rating									
$${{\tau }_{TCSC1}}\%$$		0	50	30.198	27.673	28.675	44.675	30.876	34.455
$${{\varphi }_{TCPS1}}$$ (deg)		− 5	5	− 2.245	2.134	− 2.459	3.987	3.2231	− 3.099
$${{Q}_{SVC1}}$$ (MVAR)		− 10	10	9.654	9.987	9.098	9.9875	9.112	9.675
FACTs location									
TCSC1				27–28	24–25	34–35	27–28	37–39	34–35
TCPS1				24–26	24–25	28–29	24–26	25–30	24–25
SVC1				7	21	27	7	44	24
Total generation				643.3639	756.7653	884.8675	1002.4	1136.7723	1260.577
Total generation cost ($/h)				2697.1073	3753.2185	4741.7114	5850.9633	7859.3297	9805.3018
Emission (t/h)				0.2571	0.3111	0.4094	0.5412	0.7247	0.9559
$${P}_{loss}(MW)$$				17.08	6.27	9.28	10.26	11.04	13.08
VD (p.u)				1.6381	1.4186	2.1618	1.4688	3.7776	1.2077
Frequency deviation with load									
	% max load			OS		SS (s)			
	100			0.009389		16.39			
	90			0.009713		9.98			
	80			0.009706		8.07			
	70			0.009837		8.58			
	60			0.009381		6.97			
Frequency deviation	50			0.009036		5.77

**Table 11:** ANOVA table for case 12.

At 50% load					
Source	SS	df	MS	F	P value
Columns	0.00582	2	0.00291	3.91	0.06
Error	0.00671	9	0.00075		
Total	0.01253	11			
At 80% load					
Source	SS	df	MS	F	P value
Columns	0.00471	2	0.00236	5.52	0.0273
Error	0.00384	9	0.00043		
Total	0.00856	11

**Table 12:** ANOVA table for case 13.

At 50% load					
Source	SS	df	MS	F	P value
Columns	107.795	2	53.8973	4.09	0.0546
Error	118.714	9	13.1905		
Total	226.509	11			
At 80% load					
Source	SS	df	MS	F	P value
Columns	73.269	2	36.6343	2.8	0.1135
Error	117.833	9	13.0925		
Total	191.101	11

**Table 13:** Undershoot (US), overshoot (OS) and settling time (ST) using PID & FOPID controller for 50% load.

	Parameters	Unilateral contract					Bilateral contract				
Function		DTBO tuned	DTBO tuned	DTBO tuned PID	DTBO tuned FOPID	DTBO tuned FOPID	DTBO tuned	DTBO tuned	DTBO tuned PID	DTBO tuned FOPID	DTBO tuned FOPID
		PID with	PID with	With thermal	With thermal	With thermal, RES	PID with	PID with	With thermal	With thermal	With thermal, RES
		Thermal	Thermal & FACTS	RES & FACTS	RES & FACTS	ESS & FACTS	Thermal	Thermal & FACTS	RES & FACTS	RES & FACTS	ESS & FACTS
	OS	0.0436	0.03833	0.06091	0.03573	0.02979	0.0436	0.009158	0.04411	0.009151	0.002574
$\Delta {{f}_{1}}$	US	− 0.02111	− 0.01464	− 0.04862	− 0.01386	− 0.01226	− 0.07187	− 0.0641	− 0.07136	− 0.06194	− 0.05319
	ST	26.47	21.42	38.78	14.62	13.82	47.31	31.74	39.24	26.26	22.81
	OS	0.06065	0.04398	0.03975	0.03619	0.029	0.06544	0.05615	0.08701	0.05077	0.04364
$\Delta {{f}_{2}}$	US	− 0.04872	− 0.02114	− 0.01434	0.01627	− 0.01418	− 0.03093	− 0.02307	− 0.07213	− 0.02433	− 0.02119
	ST	38.94	25.04	19.91	15.35	13.7	28.31	22.05	39.4	18.24	13.32
OBJ		0.1161	0.0813	0.1775	0.0475	0.0306	0.2570	0.1796	0.4366	0.1066	0.0682

**Table 14:** US, OS and ST using PID and FOPID controller for 80% load.

	Parameters	Unilateral contract					Bilateral contract				
Function		DTBO tuned	DTBO tuned	DTBO tuned PID	DTBO tuned FOPID	DTBO tuned FOPID	DTBO tuned	DTBO tuned	DTBO tuned PID	DTBO tuned FOPID	DTBO tuned FOPID
		PID with	PID with	With thermal	With thermal	With thermal,RES	PID with	PID with	With thermal	With thermal	With thermal, RES
		Thermal	Thermal & FACTS	RES & FACTS	RES & FACTS	ESS & FACTS	Thermal	Thermal & FACTS	RES & FACTS	RES & FACTS	ESS & FACTS
	OS	0.06611	0.05494	0.09152	0.05181	0.04484	0.06533	0.05679	0.08721	0.05225	0.04448
$\Delta {{f}_{1}}$	US	− 0.02917	− 0.01833	− 0.07025	− 0.0213	− 0.02058	− 0.02928	− 0.01954	− 0.07066	− 0.0235	− 0.02129
	ST	22.16	18.45	37.71	16.6	12.74	23.38	17.51	39.58	15.59	14.05
	OS	0.04175	0.03617	0.06075	0.03392	0.02903	0.009105	0.0001055	0.04479	0.000757	0.0004025
$\Delta {{f}_{2}}$	US	− 0.01938	− 0.01279	− 0.0466	− 0.01318	− 0.01008	− 0.06673	− 0.05549	− 0.07704	− 0.04594	− 0.03725
	ST	20.22	16.24	38.24	11.58	10.88	32.63	21.54	38.32	16.66	14.63
OBJ		0.1111	0.0771	0.1766	0.0454	0.0293	0.2511	0.1748	0.4660	0.1036	0.0670

**Table 15:** OPF result for the IEEE 57 bus test system for six load scenarios without the frequency security restriction (Case 3).

	Control								
	Parameters	Min.	Max.	50%	60%	70%	80%	90%	100%
	PTG1	0	575.88	253.46	331.38	345.6	338.15	375.56	428.46
	PTG2	0	100	95.35	98.32	0	98.37	97.87	97.91
	PTG3	0	140	75.17	81.45	87.95	92.29	121.73	131.67
	PTG6	0	100	0	0	97.42	97.6	98.05	98.54
	PTG8	0	550	61.36	54.89	53.32	81.63	76.06	88.21
	PTG9	0	100	0	0	95.44	98.32	99.26	97.6
Power (MW)	PTG12	0	410	155.29	207.11	209.67	213.91	281.63	338.44
	$${V}_{1}$$	0.95	1.1	1.0362	1.0408	1.0391	1.058	1.0543	1.0519
	$${V}_{2}$$	0.95	1.1	1.0347	1.0531	1.0332	1.0513	1.0513	1.0581
	$${V}_{3}$$	0.95	1.1	1.0161	1.0245	1.0334	1.0451	1.0405	1.0533
	$${V}_{6}$$	0.95	1.1	0.9872	0.9891	1.0199	1.0345	1.0181	1.0297
	$${V}_{8}$$	0.95	1.1	0.9847	0.9846	1.0058	1.0277	1.0005	1.0242
	$${V}_{9}$$	0.95	1.1	0.9957	0.9905	1.0005	1.0272	1.0068	1.0243
Voltage (p.u.)	$${V}_{12}$$	0.95	1.1	1.0122	1.0145	1.0086	1.0272	1.0165	1.0343
	$${T}_{19}$$(4-18)	0.9	1.1	0.9458	0.9463	0.9197	0.9	0.9148	0.9756
	$${T}_{20}$$(4-18)	0.9	1.1	0.934	0.9	1.0072	1.0293	0.9848	0.9
	$${T}_{31}$$(21-20)	0.9	1.1	0.995	1.0135	0.9977	1.025	1.0482	1.0274
	$${T}_{35}$$(24-25)	0.9	1.1	1.0085	0.9602	0.9856	0.9925	0.9	0.9
	$${T}_{36}$$(24-25)	0.9	1.1	0.9639	0.9956	1.0424	0.9	0.9178	0.9439
	$${T}_{37}$$(24-26)	0.9	1.1	1.0437	1.0978	1.0444	1.0558	1.0677	1.0528
	$${T}_{41}$$(7-29)	0.9	1.1	0.9738	0.9878	1.0201	0.996	0.9814	0.9632
	$${T}_{46}$$(34-32)	0.9	1.1	0.9787	0.9485	1.0058	0.9301	0.9	0.9
	$${T}_{54}$$(11-41)	0.9	1.1	0.9775	0.9315	0.9	0.9	0.9	0.9
	$${T}_{58}$$(15-45)	0.9	1.1	0.9492	0.9	0.9427	0.9192	0.9	0.9
	$${T}_{59}$$(14-46)	0.9	1.1	0.9354	0.9	0.9713	0.9369	0.9	0.9
	$${T}_{65}$$(10-51)	0.9	1.1	0.9649	0.9064	0.957	0.9516	0.9	0.9088
	$${T}_{66}$$(13-49)	0.9	1.1	0.9119	0.9	0.9383	0.9	0.9	0.9
	$${T}_{71}$$(11-43)	0.9	1.1	0.9317	0.9168	0.9711	0.9681	0.9	0.9
Tap setting (p.u)	$${T}_{73}$$(40-56)	0.9	1.1	1.0626	1.0492	1.011	1.0503	1.0557	1.018
	$${Q}_{C18}$$	0	5	4.4	0.0493	0.0343	0.0496	0.0491	0.0461
	$${Q}_{C25}$$	0	5	4.43	0.0489	0.0483	0.0448	0.0433	0.0491
(MVAr)	$${Q}_{C53}$$	0	5	4.66	0.0477	0.0465	0.0473	0.0499	0.0498
Total generation				640.6259	773.1417	889.4145	1020.2609	1150.1658	1280.8175
Total generation cost ($/h)				2468.7421	3129.6248	3488.7915	4142.6391	5027.3086	5983.0761
Emission (t/h)				0.5599	0.8607	0.9216	0.8986	1.1563	1.5124
Ploss (MW)				15.23	0.2266	0.1385	0.1962	0.2445	0.3002
VD (p.u.)				1.2762	2.7278	1.0482	2.4771	3.0808	3.757
Frequency deviation with load									
	% max load			OS		SS (s)			
	100			0.009447		17.14			
	90			0.009711		10.02			
	80			0.009734		8.35			
	70			0.009874		8.61			
	60			0.009885		7.74			
Frequency deviation	50			0.01094		6.86

**Table 16:** OPF result for IEEE 57 bus test system with frequency security restrictions for six load situations (Case 7).

	Control								
	Parameters	Min.	Max.	50%	60%	70%	80%	90%	100%
	PTG1	100	575.88	182.24	198.09	270.32	322.48	358.65	439.24
	PTG2	30	100	77.05	99.28	99.26	99.53	99.97	99.39
	PTG3	30	140	31.26	64.32	77.69	101.22	107.96	120.46
	PTG6	30	100	96.59	97.4	98.9	99.21	99.96	99.89
	PTG8	30	550	36.72	40.35	51.64	57.39	72.25	88.3
	PTG9	30	100	92.15	94.87	92.54	99.11	99.87	99.75
Power (MW)	PTG12	100	410	116.84	165.96	199.86	240.7	309.23	333.95
	$${V}_{1}$$	0.95	1.1	1.0297	1.0539	1.0544	1.06	1.0592	1.0596
	$${V}_{2}$$	0.95	1.1	1.037	1.0597	1.0549	1.0584	1.0594	1.0587
	$${V}_{3}$$	0.95	1.1	1.0269	1.049	1.035	1.0471	1.0468	1.0545
	$${V}_{6}$$	0.95	1.1	1.0236	1.0509	1.0187	1.0271	1.0237	1.0276
	$${V}_{8}$$	0.95	1.1	1.0045	1.0414	1.0224	1.0114	1.0145	1.031
	$${V}_{9}$$	0.95	1.1	1.0075	1.0407	1.0248	1.0128	1.0229	1.0321
Voltage (p.u.)	$${V}_{12}$$	0.95	1.1	0.9995	1.0342	1.0214	1.0239	1.0289	1.0427
	$${T}_{19}$$(4-18)	0.9	1.1	0.9172	0.9	0.9938	0.9	0.9	0.9
	$${T}_{20}$$(4-18)	0.9	1.1	1.0609	1.0178	0.9883	0.9859	0.9	0.9
	$${T}_{31}$$(21-20)	0.9	1.1	1.0045	0.9836	1.0572	1.0201	0.9952	0.9891
	$${T}_{35}$$(24-25)	0.9	1.1	0.919	0.9897	0.9125	0.9297	0.9347	0.9047
	$${T}_{36}$$(24-25)	0.9	1.1	0.9188	0.9	0.9655	0.9044	0.9136	0.9383
	$${T}_{37}$$(24-26)	0.9	1.1	0.9993	0.9973	0.9869	1.0525	1.0831	1.0775
	$${T}_{41}$$(7-29)	0.9	1.1	1.0046	0.9991	0.9629	0.9804	0.982	0.9781
	$${T}_{46}$$(34-32)	0.9	1.1	0.9	0.9043	0.908	0.9	0.9	0.9
	$${T}_{54}$$(11-41)	0.9	1.1	1.0173	0.9054	0.9143	0.9287	0.9	0.9
	$${T}_{58}$$(15-45)	0.9	1.1	0.9988	0.9883	0.9564	0.9275	0.9	0.9
	$${T}_{59}$$(14-46)	0.9	1.1	0.9988	0.9902	0.9281	0.9173	0.9	0.9
	$${T}_{65}$$(10-51)	0.9	1.1	0.9824	1.0172	0.9466	0.9336	0.9056	0.911
	$${T}_{66}$$(13-49)	0.9	1.1	0.967	0.9565	0.9186	0.9	0.9	0.9
	$${T}_{71}$$(11-43)	0.9	1.1	1.0019	0.9977	0.9151	0.9	0.9	0.9
Tap setting (p.u)	$${T}_{73}$$(40-56)	0.9	1.1	1.0636	0.9488	0.9641	1.0052	1.0103	1.0142
	$${Q}_{C18}$$	0	5	0.31	1.15	1.62	3.53	4.33	4.99
	$${Q}_{C25}$$	0	5	4.21	4.98	4.34	4.97	4.98	5
(MVAr)	$${Q}_{C53}$$	0	5	4.96	4.62	4.21	4.95	4.99	4.97
Total generation				632.85	760.2702	890.2143	1019.6458	1147.8851	1280.9869
Total generation cost ($/h)				2066.7378	2725.7624	3371.9775	4128.0755	5012.7115	5921.5041
Emission (t/h)				0.366	0.4337	0.6448	0.8748	1.124	1.5029
$${P}_{loss}$$ (MW)				7.45	9.79	14.65	19.01	22.17	30.19
VD (p.u)				1.1859	1.4925	2.1603	2.9963	3.8561	4.1678
Frequency deviation with load									
	% max load			OS		SS (s)			
	100			0.009393		16.07			
	90			0.009697		9.89			
	80			0.009608		8.11			
	70			0.009832		8.53			
	60			0.009722		7.15			
Frequency deviation	50			0.009681		6.02

**Table 17:** OPF result for variable load situations with TCSC, TCPS, and SVC for the IEEE 57 bus test system while taking the frequency security constraint into account (Case 11).

	Control								
	Parameters	Min.	Max.	50%	60%	70%	80%	90%	100%
	PTG1	100	575.88	178.55	243.32	259.97	344.9	348.12	434.29
	PTG2	30	100	73.93	98.24	99.45	100	98.65	100
	PTG3	30	140	36.05	70.59	58.07	98.34	109.2	121.84
	PTG6	30	100	103.98	97.94	99.83	98.35	98.46	100
	PTG8	30	550	38.46	44.81	38.72	54.89	97.86	94.6
	PTG9	30	100	94.97	85.15	100	100	100	100
Power (MW)	PTG12	100	410	107.4	125.91	231.85	226.28	294.49	330.73
	$${V}_{1}$$	0.95	1.1	1.0472	1.0013	1.0069	1.049	1.06	1.0587
	$${V}_{2}$$	0.95	1.1	1.0465	1.0145	1.0079	1.0379	1.054	1.06
	$${V}_{3}$$	0.95	1.1	1.0119	0.9955	0.999	1.0331	1.0464	1.0419
	$${V}_{6}$$	0.95	1.1	1.002	0.9989	0.9975	1.0136	1.0316	0.9996
	$${V}_{8}$$	0.95	1.1	0.9857	0.9751	0.9899	1.0049	1.0317	0.9943
	$${V}_{9}$$	0.95	1.1	1.0062	0.9658	0.987	1.011	1.0364	0.9992
Voltage (p.u.)	$${V}_{12}$$	0.95	1.1	1.012	0.9794	0.9929	1.0268	1.0445	1.0185
	$${T}_{19}$$(4-18)	0.9	1.1	1.0717	1.0453	0.9395	0.9078	0.9741	0.912
	$${T}_{20}$$(4-18)	0.9	1.1	0.9023	0.9191	0.9	0.9202	0.9586	0.9
	$${T}_{31}$$(21-20)	0.9	1.1	0.982	0.9196	0.9715	0.9123	0.9961	0.9868
	$${T}_{35}$$(24-25)	0.9	1.1	0.9544	0.9109	0.9879	0.9127	0.9584	0.916
	$${T}_{36}$$(24-25)	0.9	1.1	1.0906	1.0315	0.9337	0.9582	1.0379	0.9036
	$${T}_{37}$$(24-26)	0.9	1.1	0.9631	1.0208	1.027	0.9231	0.9907	1.0673
	$${T}_{41}$$(7-29)	0.9	1.1	0.9298	1.0813	1.0079	0.9719	0.9598	0.9758
	$${T}_{46}$$(34-32)	0.9	1.1	0.9736	1.0707	1.0366	1.0236	1.0838	0.9
	$${T}_{54}$$(11-41)	0.9	1.1	1.0958	1.0553	0.9224	0.9847	0.9499	0.9219
	$${T}_{58}$$(15-45)	0.9	1.1	1.0312	0.973	0.9639	1.0435	0.9484	0.9209
	$${T}_{59}$$(14-46)	0.9	1.1	0.9829	0.9895	0.9568	1.0347	0.9593	0.9269
	$${T}_{65}$$(10-51)	0.9	1.1	1.0082	1.0072	0.9873	1.034	0.9744	0.9228
	$${T}_{66}$$(13-49)	0.9	1.1	0.9887	0.997	0.9318	1.001	0.9309	0.9121
	$${T}_{71}$$(11-43)	0.9	1.1	0.9393	0.964	1.0019	0.9869	0.9348	0.9
Tap setting	$${T}_{73}$$(40-56)	0.9	1.1	0.9371	1.0571	1.0105	0.9243	0.9338	1.0034
	$${Q}_{C18}$$	0	5	3.34	4.83	4.28	0.048	0.0377	0.05
	$${Q}_{C25}$$	0	5	5	3.58	4.88	0.0489	0.0477	0.05
(MVAr)	$${Q}_{C53}$$	0	5	5	4.84	4.22	0.0415	0.0461	0.2181
FACTs rating									
$${{\tau }_{TCSC1}}\%$$		0	50	29.564	27.688	28.675	43.998	30.231	34.466
$${{\varphi }_{TCPS1}}$$ (deg)		− 5	5	− 2.564	2.342	− 2.554	3.897	3.453	− 3.123
$${{Q}_{SVC1}}$$ (MVAR)		− 10	10	9.774	9.897	9.349	9.3427	9.3421	9.5643
FACTs location									
TCSC1				27–28	24–25	34–35	27–28	37–39	34–35
TCPS1				24–26	24–25	28–29	24–26	25–30	24–25
SVC1				7	21	27	7	44	24
Total generation				633.435	765.9496	890.3551	1022.756	1147.7906	1282.5754
Total cost ($/h)				2059.0553	2698.463	3367.9403	4084.463	5006.0658	5903.8709
Emission				0.3642	0.4294	0.6356	0.8404	1.1006	1.479
$${P}_{loss}$$(MW)				8	15.46	14.78	22.07	22.05	31.77
VD (p.u)				1.2433	3.5587	1.0734	1.661	1.7884	2.1058
Frequency deviation with load									
	% max load			OS		SS (s)			
	100			0.009391		15.94			
	90			0.009686		9.83			
	80			0.009606		8.08			
	70			0.009831		8.51			
	60			0.009707		6.86			
Frequency deviation	50			0.009601		5.96

**Table 18:** Solution of the OPF for variable load scenarios with the frequency security restriction for the IEEE 57 bus test system incorporating RES, ESS & FACTS (Case 13).

	Control								
	Parameters	Min.	Max.	50%	60%	70%	80%	90%	100%
	PTG1	100	575.88	102.44	272.67	357.47	424.62	445.97	522.88
	PTG2	30	100	99.43	61.02	84.72	95.98	99.18	99.11
	PTG3	30	140	34.09	30	35.03	30	30	78.09
	PTG6	30	100	93.92	98.21	99.5	97.09	97.65	92.95
	PTG8	30	550	40.26	33.82	35.79	30	43.45	46.23
	PTG9	30	100	81.12	89.97	84.73	87.86	95.7	98.9
	PTG12	100	410	108.01	100	100	164.75	220.93	224.84
	PGW16	0	75	74.86	67.7	69.66	70.7	66.94	67.23
	PPVG45	0	50	41.44	49.12	48.45	42.28	49.75	49.55
	PAEFC19	− 10	10	− 10	− 10	0	0	10	10
Power (MW)	PUC20	− 5	5	− 5	− 5	0	0	5	5
	$${V_{1}}$$	0.95	1.1	1.0099	1.0453	1.0637	1.051	1.051	1.0955
	$${V_{2}}$$	0.95	1.1	1.0166	1.039	1.0697	1.0699	1.0554	1.093
	$${V_{3}}$$	0.95	1.1	0.9924	1.064	1.019	1.0346	1.0165	1.0647
	$${V_{6}}$$	0.95	1.1	1.0035	1.038	1.0156	0.9766	0.9877	1.0207
	$${V_{8}}$$	0.95	1.1	0.9929	1.0287	1.0103	0.9752	0.9791	1.0107
	$${V_{9}}$$	0.95	1.1	0.9983	1.0358	1.0186	0.9775	0.9933	1.0186
Voltage (p.u.)	$${V_{12}}$$	0.95	1.1	0.9925	1.012	1.0106	1.0007	1.0056	1.0137
	$${T_{19}}$$(4–18)	0.9	1.1	0.9806	0.971	1.0983	1.0079	0.9267	0.996
	$${T_{20}}$$(4–18)	0.9	1.1	0.9523	1.0714	0.9845	1.0212	1.0207	0.9417
	$${T_{31}}$$(21–20)	0.9	1.1	0.9942	0.9984	0.9915	1.0727	0.9643	0.9
	$${T_{35}}$$(24–25)	0.9	1.1	0.9554	1.0169	0.9372	1.0547	0.9572	0.9258
	$${T_{36}}$$(24–25)	0.9	1.1	0.9	1.0323	0.913	1.0596	1.0499	0.9124
	$${T_{37}}$$(24–26)	0.9	1.1	1.0855	0.9979	0.9183	1.0018	1.0076	0.9679
	$${T_{41}}$$(7–29)	0.9	1.1	1.0876	1.014	0.9441	0.9417	1.0306	1.002
	$${T_{46}}$$(34–32)	0.9	1.1	0.9477	0.9993	0.9204	0.9739	0.9222	0.9
	$${T_{54}}$$(11–41)	0.9	1.1	0.911	0.9996	1.0938	1.0095	0.9072	1.0006
	$${T_{58}}$$(15–45)	0.9	1.1	0.9608	1.025	1.0694	1.0556	1.0529	1.0888
	$${T_{59}}$$(14–46)	0.9	1.1	0.9555	1.0285	1.0231	1.0303	0.9721	1.0038
	$${T_{65}}$$(10–51)	0.9	1.1	0.9735	1.0154	1.0268	0.96	0.9641	0.9852
	$${T_{66}}$$(13–49)	0.9	1.1	1.0359	1.0146	1.0275	1.0067	1.0723	1.0995
	$${T_{71}}$$(11–43)	0.9	1.1	0.9464	1.0292	0.9744	0.9088	0.9199	0.9
	$${T_{73}}$$(40–56)	0.9	1.1	0.9218	0.9742	0.9854	1.0296	0.9179	0.9976
	$${T_{76}}$$(39–57)	0.9	1.1	0.9696	1.0314	1.0327	1.073	0.9041	0.9218
Tap setting (p.u)	$${T_{80}}$$(9–55)	0.9	1.1	1.0791	1.065	0.9824	0.9594	1.0173	1.0232
	$${Q_{C18}}$$	0	20	2.81	2.82	8.98	1.66	4.16	0.75
	$${Q_{C25}}$$	0	20	2.74	6.39	5.54	9.79	7.19	5.35
(MVAr)	$${Q_{C53}}$$	0	20	9.99	7.58	4.22	9.26	11.87	6.92
FACTs rating									
$${{\tau }_{TCSC1}}\%$$		0	50	22.56	28.98	30.76	37.56	28.56	33.56
$${{\varphi }_{TCPS1}}$$ (deg)		− 5	5	− 3.234	1.02	3.9	− 4.65	2.01	− 0.99
$${{Q}_{SVC1}}$$ (MVAR)		− 10	10	8.874	9.78	7.45	5.98	4.78	8.56
FACTs location									
TCSC1				24–25	27–28	27–28	34–35	37–39	24–25
TCPS1				28–29	25–30	24–25	24–26	24–26	24–25
SVC1				21	31	55	47	7	25
Thermal cost ($/h)				1851.400	2210.188	2696.562	3393.256	4006.725	4774.296
Wind cost ($/h)				102.2489	86.989	91.0209	93.212	85.4508	86.0255
PV cost ($/h)				123.029	146.0573	144.049	125.5578	147.9605	147.3697
AEFC cost ($/h)				− 34.24	− 30	0	0	40	40
UC cost ($/h)				− 15	− 15	0	0	20	20
Total generation cost ($/h)				2027.431	2398.235	2931.632	3612.026	4300.136	5067.691
Emission (t/h)				0.2615	0.5747	0.8774	1.2227	1.3877	1.8314
Ploss (MW)				7.68	15.02	23.28	31.65	33.35	43.99
VD (p.u.)				1.4283	1.3777	1.4811	1.2856	1.4352	1.5129

**Fig. 10:**
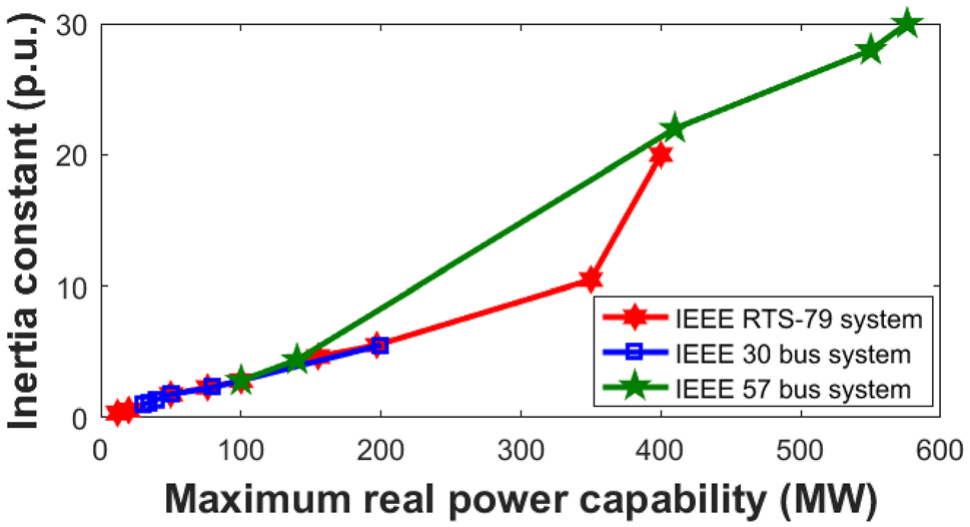
IEEE RTS-79, IEEE-30 bus and IEEE-57 bus system generator data.

**Fig. 11:**
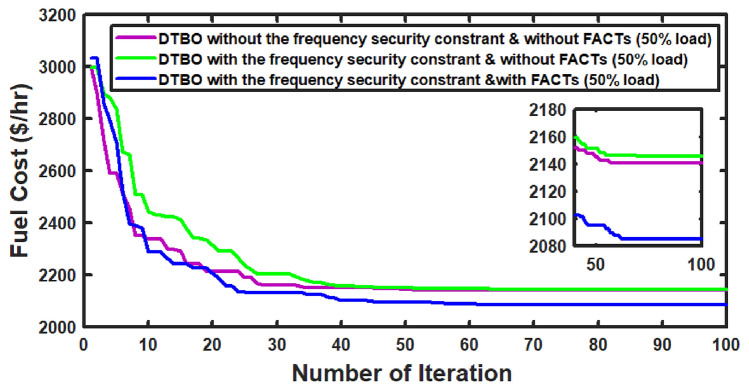
The convergence of the DTBO technique for IEEE-57 bus networks with 50% load.

**Fig. 12:**
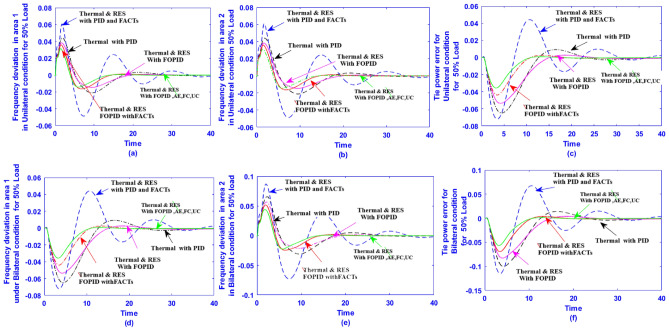
Frequency deviation of area-1 for 50% load in unilateral condition with frequency security constraint. (**b**) Frequency deviation of area-2 for 50% load in unilateral condition with frequency security constraint. (**c**) Power error for 50% load in unilateral condition with frequency security constraint. (**d**) Frequency deviation of area-1 for 50% load in Bilateral condition with frequency security constraint. (**e**) Frequency deviation of area-2 for 50% load in Bilateral condition with frequency security constraint. (**f**) Power error for 50% load in Bilateral condition with frequency security constraint.

**Fig. 13:**
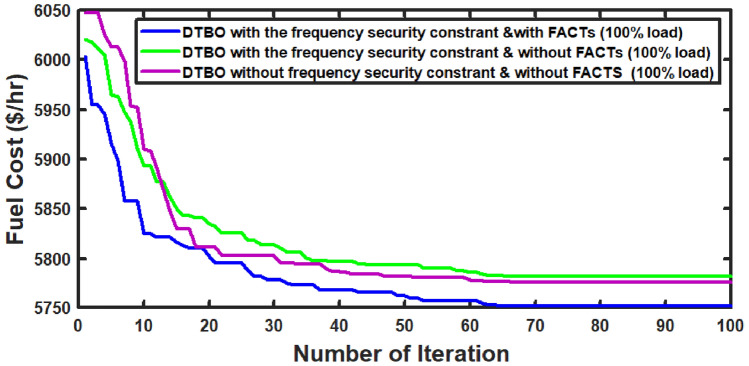
The convergence of the DTBO technique for IEEE-57 bus network with a 100% load.

At the $3^{rd}$ DTBO phase, $Z_{p}^{st3}$ is a modified ${{p}^{th}}$ possible solution; $z_{p,q}^{st3}$ is its ${{q}^{th}}$ variable; the associated objective function’s value is $F_{p}^{st3}$; *r* is an arbitrary number between 0 and 1; *R* is 0.05, *s* is the current iteration, and *S* is the maximum iteration. After completing one DTBO iteration, steps one through three upgrade the DTBO population. The following iteration then starts with a currently updated population, and this process repeats [through (73) to (81)] until the conclusion of the final iteration. After final iteration, the best possible selection can be determined as the solution to the problem. The DTBO flowchart is displayed in Fig. 9 (Tables 5, 6, 7, 8).

**Table 19:** Solution of the OPF for variable load scenarios with the frequency security constraint for the IEEE 57 bus test system incorporating RES,ESS & FACTS (Case 15).

	Control								
	Parameters	Min.	Max.	50%	60%	70%	80%	90%	100%
	PTG1	100	575.88	178.26	195.97	230.8	270.03	288.43	362.73
	PTG2	30	100	55.95	89.3	88.44	96.91	99.09	98.64
	PTG3	30	140	30	51.81	65.45	106.93	109.31	116.04
	PTG6	30	100	90.21	89.22	95.13	96.52	97.87	96.24
	PTG8	30	550	30	39.4	40.11	56.39	83.01	71.63
	PTG9	30	100	70.97	91.06	95.74	96.26	95.94	99.17
	PTG12	100	410	100	130.21	175.55	186.54	244.82	302.77
	PGW16	0	75	73.54	68.81	68.5	74.61	69.62	74.56
	PPVG45	0	50	48.7	44.38	46.9	48.14	49.77	44.27
	PAEFC19	− 10	10	− 10	− 10	0	0	10	10
Power (MW)	PUC20	− 5	5	− 5	− 5	0	0	5	5
	V1	0.95	1.1	1.0576	1.0557	1.0248	1.0476	1.0615	1.0484
	V2	0.95	1.1	1.0697	1.0456	1.0241	1.0583	1.0814	1.0566
	V3	0.95	1.1	1.0549	1.0218	1.018	1.0786	1.0521	1.0332
	V6	0.95	1.1	1.0466	1.0004	1.0117	1.0447	1.0143	0.9941
	V8	0.95	1.1	1.0438	0.9757	0.9908	1.0287	0.9959	0.9734
	V9	0.95	1.1	1.0203	1.0111	0.989	1.0249	1.0029	0.9756
Voltage (p.u.)	V12	0.95	1.1	1.0374	1.0176	0.9995	1.0142	1.0064	1.011
	$${T_{19}}$$(4-18)	0.9	1.1	0.9088	0.9318	0.9085	0.9274	1.0715	1.0832
	$${T_{20}}$$(4-18)	0.9	1.1	0.9179	0.9423	0.9564	0.9915	1.0925	1.0756
	$${T_{31}}$$(21-20)	0.9	1.1	0.9007	0.9796	0.9225	0.9221	1.0263	1.0984
	$${T_{35}}$$(24-25)	0.9	1.1	0.9463	1.052	0.9499	0.9343	1.0158	1.0184
	$${T_{36}}$$(24-25)	0.9	1.1	1.0897	1.0355	0.999	1.0911	0.9338	1.0712
	$${T_{37}}$$(24-26)	0.9	1.1	1.0107	0.9558	0.9979	0.9712	0.9152	0.9438
	$${T_{41}}$$(7-29)	0.9	1.1	1.0497	0.9137	1.0276	1.0148	0.94	0.9626
	$${T_{46}}$$(34-32)	0.9	1.1	0.9303	1.0025	0.9377	0.9655	0.9334	1.0358
	$${T_{54}}$$(11-41)	0.9	1.1	1.0122	0.9387	1.0818	0.9322	0.9072	0.9205
	$${T_{58}}$$(15-45)	0.9	1.1	1.0158	0.9638	1.0102	1.077	1.0184	1.0952
	$${T_{59}}$$(14-46)	0.9	1.1	1.0723	1.0131	1.004	1.0192	1.0057	0.9917
	$${T_{65}}$$(10-51)	0.9	1.1	1.0043	1.0261	0.952	1.0056	0.9455	1.056
	$${T_{66}}$$(13-49)	0.9	1.1	0.9456	1.0528	1.0193	0.9923	1.0692	1.0116
	$${T_{71}}$$(11-43)	0.9	1.1	1.0805	0.959	0.9327	0.9077	0.9	0.9627
	$${T_{73}}$$(40-56)	0.9	1.1	1.0786	0.9939	1.0504	0.9	0.9398	0.992
	$${T_{76}}$$(39-57)	0.9	1.1	1.0152	1.0013	1.0208	0.9092	0.9548	0.9354
Tap setting	T80(9-55)	0.9	1.1	1.0505	0.936	0.9867	1.0248	0.9774	0.9649
	QC18	0	20	2.08	1.06	2.72	1.57	4.76	1.83
	QC25	0	20	8.8	5.47	7.48	7.36	6.66	9.1
(MVAr)	QC53	0	20	8.97	8.52	3.24	2.53	6.67	7.31
FACTs rating									
$${{\tau }_{TCSC1}}\%$$		0	50%	42.89	34.67	33.76	12.67	23.87	29.897
$${{\varphi }_{TCPS1}}$$ (deg)		− 5	5	3.89	2.76	− 2.98	− 3.78	2.09	2.99
$${{Q}_{SVC1}}$$ (MVAR)		− 10	10	8.78	5.76	4.77	1.89	5.89	6.78
FACTs location									
TCSC1				34–35	54–55	41–43	48–49	38–44	34–35
TCPS1				32–33	37–38	36–40	41–42	22–38	30–31
SVC1				10	7	43	20	11	19
Thermal cost ($/h)				1715.8673	2356.6788	2867.9454	3531.743	4303.1816	5033.7069
Wind cost ($/h)				99.3329	89.2551	88.6295	101.7059	90.9501	101.5995
PV cost ($/h)				144.7941	131.8515	139.4104	143.1211	148.0071	131.5169
AEFC cost ($/h)				− 30	− 30	0	0	40	40
UC cost ($/h)				− 15	− 15	0	0	20	20
Total generation cost ($/h)				1914.9943	2532.7853	3095.9853	3776.57	4602.1388	5326.8234
Emission (t/h)				0.3413	0.3947	0.5085	0.6414	0.7692	1.131
Ploss (MW)				9.73	12.69	14.56	20.68	21.64	30.25
VD (p.u.)				1.7972	1.542	1.4825	1.6643	1.5313	1.8103

**Table 20:** Thirty trials of statistical analysis comparing several methods for the IEEE 57 bus system in different scenarios.

Case	Load	Statistical parameters	DTBO	BBO	GWO
Case 9		Best (min)	**2085.0473**	**2086.3254**	**2087.5318**
		Mean (average)	2088.1532	2091.0784	2094.1164
	50%	Median	2089.6385	2092.3647	2095.6183
		Worst (max)	2091.7128	2097.1073	2102.0965
		Standard deviation	2.5704	4.1748	6.4973
		Best (min)	**3986.2751**	**3988.4275**	**3990.8351**
		Mean (average)	3989.3218	3992.6852	3996.4062
	80%	Median	3990.9154	3994.7118	3998.0537
		Worst (max)	3993.2581	3998.6227	4004.3758
		Standard deviation	2.0531	3.9867	5.8967
Case 10		Best (min)	**0.2571**	**0.3074**	**0.3975**
		Mean (average)	0.2692	0.3161	0.4116
	50%	Median	0.2851	0.3497	0.4611
		Worst (max)	0.2988	0.3632	0.4787
		Standard deviation	0.0281	0.0493	0.0678
		Best (min)	**0.5412**	**0.6275**	**0.8253**
		Mean (average)	0.6114	0.9103	1.1854
	80%	Median	0.8285	1.0755	1.3986
		Worst (max)	0.9497	1.3937	1.7593
		Standard deviation	0.1528	0.3748	0.5584
Case 11		Best (min)	**2423.2553**	**2425.8116**	**2427.6604**
		Mean (average)	2426.6411	2429.6385	2434.0633
	50%	Median	2427.8539	2431.2638	2435.1857
		Worst (max)	2430.7543	2436.5211	2441.3264
		Standard deviation	2.7361	4.3108	7.0942
		Best (min)	**5846.4658**	**5847.8336**	**5849.5728**
		Mean (average)	5850.2641	5852.7746	5856.7463
	80%	Median	5852.0368	5854.0653	5857.9674
		Worst (max)	5855.8452	5859.4781	5863.4485
		Standard deviation	3.4768	5.6241	7.8106
Case 12		Best (min)	**0.0511**	**0.0607**	**0.0726**
		Mean (average)	0.0624	0.0813	0.1115
	50%	Median	0.0681	0.0957	0.1244
		Worst (max)	0.0796	0.1164	0.1679
		Standard deviation	0.0184	0.0295	0.0487
		Best (min)	**0.0421**	**0.0581**	**0.0694**
		Mean (average)	0.0554	0.0722	0.0913
	80%	Median	0.0567	0.0799	0.1163
		Worst (max)	0.0676	0.0975	0.1386
		Standard deviation	0.0143	0.0218	0.0379
Case 13		Best (min)	**2027.4317**	**2029.1635**	**2031.0957**
		Mean (average)	2029.4175	2030.4275	2036.3174
	50%	Median	2030.7753	2034.6683	2038.8752
		Worst (max)	2032.4071	2036.2205	2042.7349
		Standard deviation	2.1652	3.9571	5.8863
		Best (min)	**3612.0265**	**3613.4274**	3**615.8253**
		Mean (average)	3615.1076	3616.4118	3620.5206
	80%	Median	3615.7452	3617.9404	3621.8952
		Worst (max)	3618.4271	3621.6453	3626.8775
		Standard deviation	2.463	3.8746	5.1304
Case 14		Best (min)	**0.2483**	**0.2681**	**0.2976**
		Mean (average)	0.3012	0.3316	0.3708
	50%	Median	0.3131	0.3554	0.4985
		Worst (max)	0.3509	0.3881	0.4758
		Standard deviation	0.0268	0.0479	0.0699
		Best (min)	** 0.4545**	**0.4793**	**0.4986**
		Mean (average)	0.6372	0.6718	0.7284
	80%	Median	0.5784	0.7203	0.9286
		Worst (max)	0.7196	0.8307	1.1642
		Standard deviation	0.1281	0.2753	0.3654
Case 15		Best (min)	**2256.2943**	**2259.6481**	**2261.7054**
		Mean (average)	2259.7413	2264.7428	2267.3183
	50%	Median	2260.7495	2265.3192	2269.7381
		Worst (max)	2263.7088	2269.8337	2276.5378
		Standard deviation	2.3861	4.6853	6.8509
		Best (min)	**4417.97**	**4419.4273**	**4421.4275**
		Mean (average)	4420.7044	4423.9705	4427.6453
	80%	Median	4421.8543	4424.5873	4428.7503
		Worst (max)	4424.6901	4428.1538	4434.6115
		Standard deviation	2.1735	3.7382	5.8624
Case 16		Best (min)	**0.0306**	**0.0428**	**0.0485**
		Mean (average)	0.0591	0.0817	0.0964
	50%	Median	0.0617	0.1028	0.1099
		Worst (max)	0.0843	0.1193	0.1386
		Standard deviation	0.0257	0.0397	0.0548
		Best (min)	**0.0293**	**0.0384**	**0.0473**
		Mean (average)	0.0376	0.0571	0.0785
	80%	Median	0.0482	0.0678	0.1089
		Worst (max)	0.0549	0.0892	0.1357
		Standard deviation	0.0136	0.0247	0.0371

**Table 21:** Thirty trials of statistical analysis comparing several methods for the IEEE 118 bus system in different scenarios.

Case	Load	Statistical parameters	DTBO	BBO	GWO
Case 17		Best (min)	75,234.177	75,311.103	75,389.564
		Mean (average)	75,239.897	75,322.675	75,403.129
	60%	Median	75,240.453	75,323.006	75,404.998
		Worst (max)	75,245.908	75,333.449	75,419.056
		Standard deviation	5.342	10.453	14.342
		Best (min)	127,856.519	127,932.775	127,987.054
		Mean (average)	127,863.564	127,944.786	128,003.809
	100%	Median	127,864.878	127,945.321	128,005.005
		Worst (max)	127,872.675	127,957.009	128,023.238
		Standard deviation	7.564	12.342	17.509

## Results of simulations and comparisons for different situations

This section represents the outcome from the OPF simulations, which is used five test systems to describe the entire study. The five distinct test systems with different scenarios are displayed in Table 1. The IEEE-57 bus system, which includes 80 transmission lines, 7 generators, 17 tap changers and 3 compensating devices, has a total demand of 1250.8 MW for active load and 336.4 MVAR for reactive load, as indicated in Table 2. Test Module 5, IEEE 118 bus as test system with wind at bus 81, solar at bus 64 and ESS at bus 117 (shown in Table 23) . The generating cost, emission and frequency fluctuation are noticed when the load is varied from 50% to 100%. Determining the frequency deviation under different loads while reducing the cost of generation and emission is the primary goal. The cost and emission factors of generators are shown in Table 7. Table 3 shows the generating cost, emission, and frequency fluctuation at various loads (50% to 100%) without frequency security requirements. For case 1, the thermal plant PTG3 and PTG8’s output power becomes to zero at 50% load. Only five of the seven power plants—PTG1, PTG2, PTG6, PTG9, and PTG12—are therefore running at this load. The inertia constant at various generation powers is shown in Fig. 10. As a result, all generators’ combined inertia will drop. The frequency fluctuation is greatest under this load because the inertia constant decreases. Moreover, with 60% of the load, the same issues arise; just five generators—PTG1, PTG2, PTG6, PTG9 and PTG12—are working; PTG3 and PTG8 are not working at all. As a result, the frequency deviation increases along with the increase in load. Nevertheless, it has been observed that all generators are operating at higher loads, specifically 70%, 80%, 90%, and 100% in this case. Thus, as the load increases, frequency deviation decreases, but the cost of generating increases (Figs. 11, 12, 13 and Tables 9, 10, 11).

**Table 22:** OPF solution for the IEEE 57 bus test system using RES, ESS, and FACTS for variable load situations with the frequency security constraint (Case 14).

	Control								
	Parameters	Min.	Max.	50%	60%	70%	80%	90%	100%
	PTG1	100	575.88	100	100	100	110.54	136.38	164.76
	PTG2	30	100	57.68	95.7	80.96	92.1	93.97	99.86
	PTG3	30	140	91.21	111.44	137.13	121.25	133.96	138.54
	PTG6	30	100	50.24	67	97.15	77.84	93.85	91.49
	PTG8	30	550	66.36	98.65	131.19	177.01	213.37	221.67
	PTG9	30	100	88.93	96.08	99.82	93.7	91.74	86.69
	PTG12	100	410	127.49	125.16	151.04	237.65	247.66	325.44
	PGW16	0	75	54	70.32	58.17	68.45	73.11	73.45
	PPVG45	0	50	43.79	44.46	48.88	48.1	45.73	49.68
	PAEFC19	− 10	10	− 10	− 10	0	0	10	10
Power (MW)	PUC20	− 5	5	− 5	− 5	0	0	5	5
	V1	0.95	1.1	1.0281	1.0857	1.0395	0.9752	1.0271	1.0534
	V2	0.95	1.1	1.0323	1.0242	1.0235	1.0049	1.0277	1.0681
	V3	0.95	1.1	1.0236	0.9922	1.0242	0.9936	1.0022	1.058
	V6	0.95	1.1	0.9979	1.0141	1.0295	1.0286	1.0233	1.0181
	V8	0.95	1.1	0.98	1.028	1.0477	1.0784	1.0091	1.0074
	V9	0.95	1.1	0.9787	1.0414	1.076	1.0739	1.0239	1.0259
Voltage (p.u.)	V12	0.95	1.1	1.0394	0.976	1.0294	1.0174	0.9999	0.9895
	$${T_{19}}$$(4-18)	0.9	1.1	0.9215	1.0298	1.0876	1.0851	1.0924	1.0938
	$${T_{20}}$$(4-18)	0.9	1.1	1.0162	1.0083	0.9796	0.9578	0.9734	0.9883
	$${T_{31}}$$(21-20)	0.9	1.1	1.0326	0.9009	0.9707	1.0602	0.9995	1.0488
	$${T_{35}}$$(24-25)	0.9	1.1	0.9608	0.9888	0.9525	1.0085	0.9037	0.9363
	$${T_{36}}$$(24-25)	0.9	1.1	0.9584	0.9837	1.0553	1.0848	1.0225	0.9313
	$${T_{37}}$$(24-26)	0.9	1.1	0.9217	0.9565	0.988	0.9731	1.0434	1.0918
	$${T_{41}}$$(7-29)	0.9	1.1	1.0009	1.0481	1.033	1.0344	1.0686	1.045
	$${T_{46}}$$(34-32)	0.9	1.1	1.0902	0.9432	0.9228	0.9259	0.9524	0.9
	$${T_{54}}$$(11-41)	0.9	1.1	1.0454	1.0062	1.0121	0.9549	0.9878	0.9245
	$${T_{58}}$$(15-45)	0.9	1.1	0.9117	1.0189	1.0212	1.0583	1.0272	1.0585
	$${T_{59}}$$(14-46)	0.9	1.1	1.0422	1.0353	0.9981	0.9853	0.9973	0.9629
	$${T_{65}}$$(10-51)	0.9	1.1	1.064	1.0873	1.0693	1.0004	0.9852	1.0061
	$${T_{66}}$$(13-49)	0.9	1.1	0.9079	1.0016	1.0354	1.0557	0.9877	1.062
	$${T_{71}}$$(11-43)	0.9	1.1	1.0256	0.9086	1.0831	1.0292	0.9328	1.0777
	$${T_{73}}$$(40-56)	0.9	1.1	0.9246	0.9525	1.0391	1.0142	0.9289	1.0416
	$${T_{76}}$$(39-57)	0.9	1.1	1.0753	0.9547	1.0102	0.9472	0.9296	1.0775
Tap setting (p.u)	T80 (9-55)	0.9	1.1	1.0619	0.9154	1.0955	1.0583	1.0787	1.0783
	QC18	0	20	12.67	4.97	17.89	7.11	2.5	2.67
	QC25	0	20	12.32	8.04	9.76	6.89	6.11	4.24
(MVAr)	QC53	0	20	18.19	14.14	5.95	5.77	7.5	11.03
FACTs rating									
$${{\tau }_{TCSC1}}\%$$		0	50%	33.76	12.89	23.89	41.09	11.76	34.56
$${{\varphi }_{TCPS1}}$$ (deg)		-5	5	4.67	3.78	4.98	2.67	-0.34	-2.78
$${{Q}_{SVC1}}$$ (MVAR)		-10	10	9.56	7.88	5.67	3.78	5.89	7.98
FACTs location									
TCSC1				21–22	25–30	28–29	28–29	34–35	54–55
TCPS1				37–39	47–48	28–29	41–43	38–44	48–49
SVC1				27	30	32	35	44	43
Thermal cost ($/h)				2260.0588	3007.531	3926.9606	5259.2521	6511.8201	7502.7041
Wind cost ($/h)				62.1275	92.4058	68.995	88.5292	98.4101	99.1451
PV cost ($/h)				130.0803	132.0941	145.3475	143.0176	135.8873	147.741
AEFC cost ($/h)				− 30	− 30	0	0	40	40
UC cost ($/h)				− 15	− 15	0	0	20	20
Total generation cost ($/h)				2407.2667	3187.0308	4141.303	5490.7988	6806.1175	7809.5902
Emission (t/h)				**0.2483**	**0.2807**	**0.3423**	**0.4545**	**0.5592**	**0.7384**
Ploss(MW)				11.8	21.33	12.28	15.02	13.55	15.79
VD(p.u.)				1.6408	1.387	1.3848	1.1679	1.6712	1.763

The key objective is to lower frequency variation’s impact on all loads while simultaneously optimize generation costs and emissions. System 1 does not take frequency security restrictions into account. One crucial factor that is now taken into consideration is the frequency security restriction. The generating cost, emission and frequency deviation under varying loads (50% to 100%) while taking frequency security restriction into account are displayed in Table 4. Because of the frequency security constraint, all generators will be able to run at any load within the range of their smallest and greatest power. Due to the growing sum of inertia constants, operating all generators simultaneously will decrease frequency fluctuation, but it will also increase generating costs (Tables 12, 13, 14, 15, 16, 17).

When the objective is to decrease the cost of generation, the frequency deviation is at its highest (OS = 0.01004 & SS = 6.3 s) at 50% load for scenario 1, and the total generation cost is decreased (*i*.*e*., 2140.3597 ($/h)). When frequency security restriction are considered, instance 5’s total cost of generation with the same load is 2145.9374 ($/h), which is more than case 1’s. The frequency fluctuation (OS = 0.0098 & SS = 5.01 s) is less, though. The frequency fluctuation (OS=0.00909 & SS=4.98) is reduced in instance 9 when FACTS are introduced, and the cost of generation is 2085.047 ($/h), which is less than in cases 1 and 5. The frequency fluctuation, emission and generating cost for case 9 (which includes FACTS with frequency security restriction) are shown in Table 5. In case 13, when incorporate RES, ESS & FOPID with frequency security constraint the cost of generation is decreased to 2027.431 ($/h) which is lower than the case 1,case 5 & case 9. Table 18 shows that the generation cost for case 13.

As shown in Table 6, when the goal is to reduce the emission at 50% load for case 2, the emission is at its lowest *i*.*e* 0.3574 (t/h) but the frequency variation is at its highest (OS = 0.01007 and SS = 6.3 s). Taking into consideration frequency-based security limitations, case 6 shows a lower emission at the same load of 0.2575 (t/h) than case 2, as well as a lower frequency deviation (OS = 0.009045 and SS = 5.80), as shown in Table 9. When FACTs (TCSC, TCPS, and SVC) are introduced in case 10, the emission is reduced to 0.2571 (t/h) which is less than in cases 2 & 6 and the frequency deviation is lowered (OS = 0.009036 & SS = 5.77). Table 10 shows the frequency fluctuation, emission and generating cost for case 10 (which includes FACTS and frequency-based security constraint). In case 14, when incorporate RES,ESS and FOPID with frequency based security constraint, the emission is reduced to 0.2483 (t/h) which is less than in cases 2, case 6 and case 10. The emission at all load for case 14 is depicted in Table 22.

It has been discovered that emissions will increase even if the cost of generation decreases when the goal is to reduce producing costs at 50% load. Also, it has been observed that when minimizing emissions is the aim, emissions are reduced at the same load, but the cost of generation goes up. To simultaneously reduce the cost of generation and emissions, multi objective are taken into consideration. However, in case 3, as indicated in Table 15, if frequency security limitations are neglected, the overall generation cost is 2468.7421 ($/h), emission is 0.5599, and frequency deviation is (OS = 0.01094 & SS = 6.86 s). When frequency-based security needs are taken into account, case 7’s total generating cost is 2066.7378 ($/h), emission is 0.366 (t/h), and frequency deviation (OS = 0.009681 & SS = 6.02 s) is less than case 3’s, as Table 16 illustrates. However, in case 11, when FACTs (TCSC, TCPS, and SVC) are included, the frequency deviation (OS = 0.009601 and SS = 5.96) shown in Table 17 is also lowered, and the generating cost and emission are both 2059.05 ($/h) and 0.3642 (t/h), respectively, which are significantly lower than in cases 3 and 7. In case 15, when incorporate RES,ESS & FOPID with frequency security constraint, the cost of generation and emission are 1914.994 ($/h) & 0.3413 (t/h) which are lower than case 3, case 7 & case 11. The cost of generation & emission for case 15 is displayed in Table 19. Thus, it may be concluded that the OPF problem’s performance is better when RES, ESS and FACTs devices (TCSC, TCPS, & SVC) with frequency constraint are incorporated.

At 60% load, When the goal is to minimize cost of generation in case 1, the frequency deviation is at its largest (OS = 0.009896 & SS = 7.6 s), while the total generation cost is at its least i.e 2764.9601 ($/h). When contemplating frequency security restriction, in case 5, overall cost of generation at the same load is 2771.232 ($/h), which is larger than case 1, but the frequency fluctuation (OS = 0.009529 & SS = 5.45 s) is smaller. When FACTS are added in case 9, the frequency fluctuation (OS = 0.009527 & SS = 5.39) is decreased and cost of generation is 2661.9802 ($/h), which is lesser than case 1 and 5. The frequency fluctuaion, emission and generating cost for case 9 (which includes TCSC, TCPS and SVC with frequency security restriction) are shown in Table 5. In case 13, when incorporate RES, ESS & FOPID with frequency security constraint the generation cost is decreased to 2398.235 ($/h) which is lesser than the case 1,5 & 9. Table 18 shows that the generation cost for case 13 (Tables 20, 21).

As shown in Table 6, when the goal is to reduce the emission at 60% load for case 2, the emission is at its lowest *i*.*e* 0.4787 (t/h) but the frequency fluctuation is at its highest (OS = 0.009594 and SS = 7.6 s). Taking into consideration frequency security restriction, case 6 shows a lower emission at the same load of 0.3158 (t/h) than case 2, as well as a lower frequency fluctuation (OS = 0.009490 and SS = 7.02), as shown in Table 9. When FACTS devices are introduced in case 10, the emission is reduced to 0.3111 (t/h) which is less than in cases 2 & 6 and the frequency fluctuation is lowered (OS = 0.009381 & SS = 6.97 sec). Table 10 shows the frequency fluctuation, emission and generating cost for case 10 (which includes FACTS and frequency security restriction). In case 14, when incorporate RES, ESS & FOPID with frequency security restriction, the emission is reduced to 0.2807 (t/h) which is less than in cases 2, case 6 & case 10. The emission at all load for case 14 is depicted in Table 22.

It has been observed that when minimizing emissions is the aim, emissions are reduced at the same load, but the cost of generation goes up. To simultaneously decrease the cost and emissions, multi objective are taken into consideration. However, in case 3, as mentioned in Table 15, if frequency security limitations are neglected, the overall generation cost is 3129.6248 ($/h), emission is 0.8607 and frequency fluctuation is (OS = 0.009885 & SS = 7.74 s). As shown in Table 16, in case 7, when frequency security requirements are contemplating, the total cost is 2725 ($/h), emission is 0.4337 (t/h) and frequency fluctuation (OS = 0.009722 & SS = 7.15 s) are lesser than in case 3. However, in case 11, when FACTs (TCSC, TCPS, and SVC) are included, the frequency fluctuation (OS = 0.009707 and SS = 6.86) shown in Table 17 is also lowered and the generating cost and emission are 2698.463 ($/h) and 0.4294 (t/h), respectively, which is lesser than in cases 3 and 7. In case 15, when incorporate RES, ESS and FOPID with frequency security limitation, the cost and emission are 2532.7853 ($/h) & 0.3947 (t/h) which are lower than case 3, case 7 and case 11. The cost of generation and emission for case 15 is displayed in Table 19. Thus, it may be concluded that the OPF problem’s performance of the OPF problem is better when RES, ESS and FACTs devices with frequency constraint are incorporated.

At 70% load, When the goal is to minimize cost of generation, in case 1, the frequency fluctuation is at its greatest (OS = 0.009837 & SS = 8.7 s), while the total generation cost is at its least *i*.*e* 3350.5598 ($/h). When contemplating frequency security restriction, in case 5, overall cost of generation at the same load is 3372.946 ($/h), which is higher than case 1, but the frequency fluctuation (OS = 0.009651 & SS = 5.21 s) is lower. When FACTS devices are added in case 9, the frequency fluctuation (OS = 0.009649 & SS = 5.18 s) is decreased and cost of generation is 3290.7677 ($/h), which is lesser than in cases 1 and 5. The frequency fluctuation, emission and generating cost for case 9 (which includes FACTS with frequency security restriction) are shown in Table 5. In case 13, when incorporate RES, ESS and FOPID with frequency security constraint the cost of generation is decreased to 2931.632 ($/h) which is lower than the case 1, case 5 & case 9. Table 18 shows that the generation cost for case 13.

As shown in Table 6, when the goal is to reduce the emission at 70% load for case 2, the emission is at its lowest *i*.*e* 0.512 (t/h) but the frequency fluctuation is at its highest (OS = 0.009867 and SS = 8.7 s). Taking into consideration frequency-based security limitations, case 6 shows a lower emission at the same load of 0.4102 (t/h) than case 2, as well as a lower frequency fluctuation (OS = 0.009838 and SS = 8.62), as shown in Table 9. When FACTs (TCSC, TCPS, and SVC) are introduced in case 10, the emission is reduced to 0.4094 (t/h) which is less than in cases 2 and 6 and the frequency deviation is lowered (OS = 0.009837 & SS = 8.58). Table 10 shows the frequency fluctuation, emission and generating cost for case 10 (which includes TCSC, TCPS, SVC, and frequency security limitation). In case 14, when incorporate RES, ESS and FOPID with frequency security limitation, the emission is reduced to 0.3423 (t/h) which is less than in cases 2, case 6 and case 10. The emission at all load for case 14 is depicted in Table 22.

It has been observed that emissions will increase even if generating costs fall when the goal is to reduce producing costs at 70% load. Also it has been observed that when minimizing emissions is the aim, emissions are reduced at the same load, but the cost of generation goes up. To simultaneously reduce the cost of generation and emissions, multi objective are taken into consideration. However, in case 3, as indicated in Table 15, if frequency security limitations are neglected, the overall generation cost is 3488.7915 ($/h), emission is 0.9216, and frequency deviation is (OS = 0.009874 & SS = 8.61 s). As shown in Table 16, in case 7, when frequency security requirements are considered, the total cost is 3371.9775 ($/h), emission is 0.6448 (t/h) and frequency fluctuation (OS = 0.009832 & SS = 8.53 s) are lower than in case 3. However, in case 11, when FACTs (TCSC, TCPS, and SVC) are included, the frequency fluctuation (OS = 0.009831 and SS = 8.51) shown in Table 17 is also lowered, and the generating cost and emission are both 3367.9403 ($/h) and 0.36356 (t/h), respectively, which are significantly lower than in cases 3 and 7. In case 15, when incorporate RES, ESS & FOPID with frequency security constraint, the generation cost and emission are 3095.9853 ($/h) & 0.5085 (t/h) which are lower than case 3, case 7 & case 11. The cost of generation & emission for case 15 is displayed in Table 19. Thus, it may be concluded that the OPF problem’s performance of the OPF problem is better when RES, ESS and FACTs devices (TCSC, TCPS, & SVC) with frequency constraint are incorporated. The same thing occurs for load percentages of 80%, 90% and 100%.

The frequency fluctuation for only thermal at different loads (50% to 100%) without frequency security constraints is shown in Fig. 20, and the frequency fluctuation for only thermal at different loads (50 to 100%) with frequency security limitation is shown in Fig. 21. Figures 15 and 16 show the voltage deviation at different loads (50 to 100%) with frequency security limitation and Figs. 17 and 18 shows that the voltage deviation at different loads (50 to 100%) with frequency security limitation and FACTs (TCSC, TCPS, and SVC) devices. The convergence graph for all loads using the DTBO algorithm is displayed in Figs. 11 and 13.

**Fig. 14:**
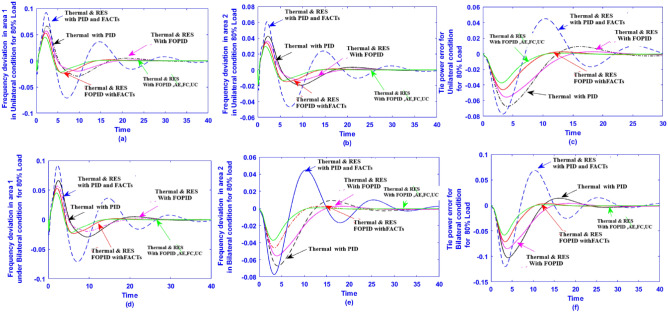
Frequency fluctuation of area-1 for 80% load in unilateral condition with frequency security limitation. (**b**) Frequency fluctuation of area-2 for 80% load in unilateral condition with frequency security limitation. (**c**) Power error for 80% load in unilateral condition with frequency security limitation. (**d**) Frequency fluctuation of area-1 for 80% load in Bilateral condition with frequency security limitation. (**e**) Frequency fluctuation of area-2 for 80% load in Bilateral condition with frequency security limitation. (**f**) Power error for 80% load in Bilateral condition with frequency security limitation.

**Fig. 15:**
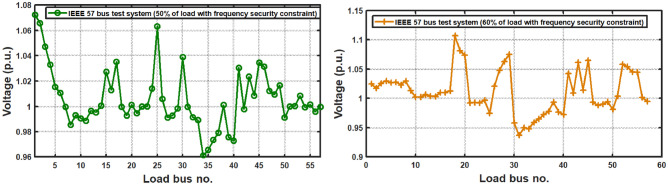
Voltage at different buses for 50 and 60% load with frequency security restriction.

**Fig. 16:**
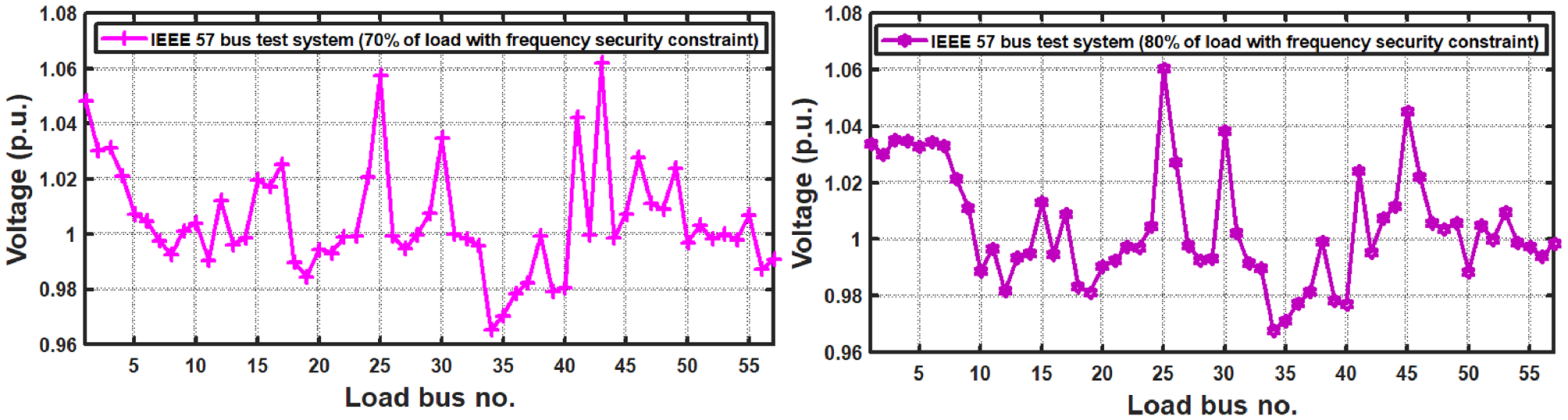
Voltage at different buses for 70 and 80% load with frequency security restriction.

**Fig. 17:**
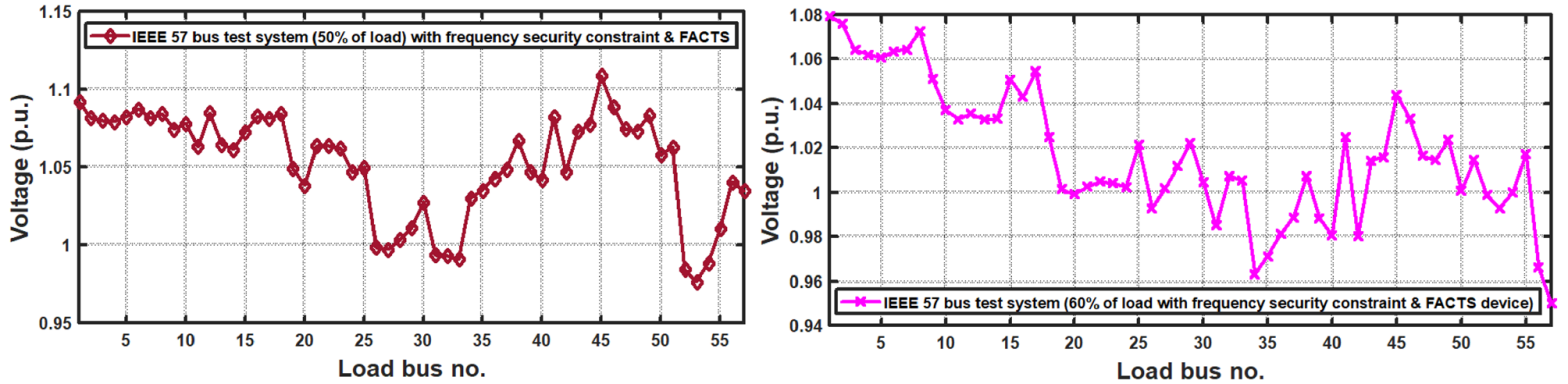
Voltage at different buses for 50 and 60% load with frequency security restriction and Facts (TCSC,TCPS & SVC).

**Fig. 18:**
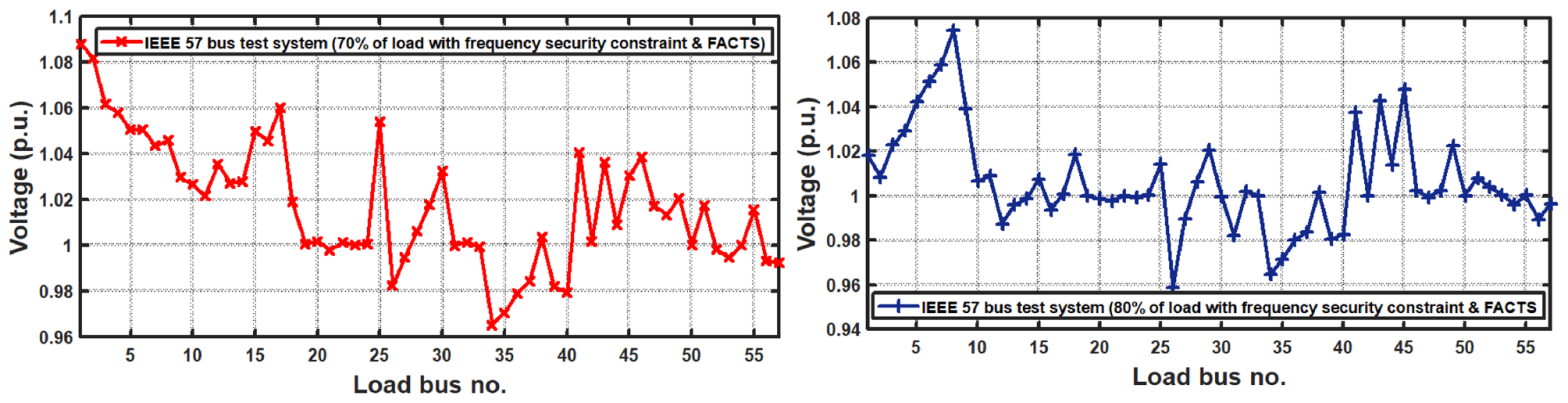
Voltage at different buses for 70 and 80% load with frequency security restriction and FACTS (TCSC,TCPS & SVC).

**Fig. 19:**
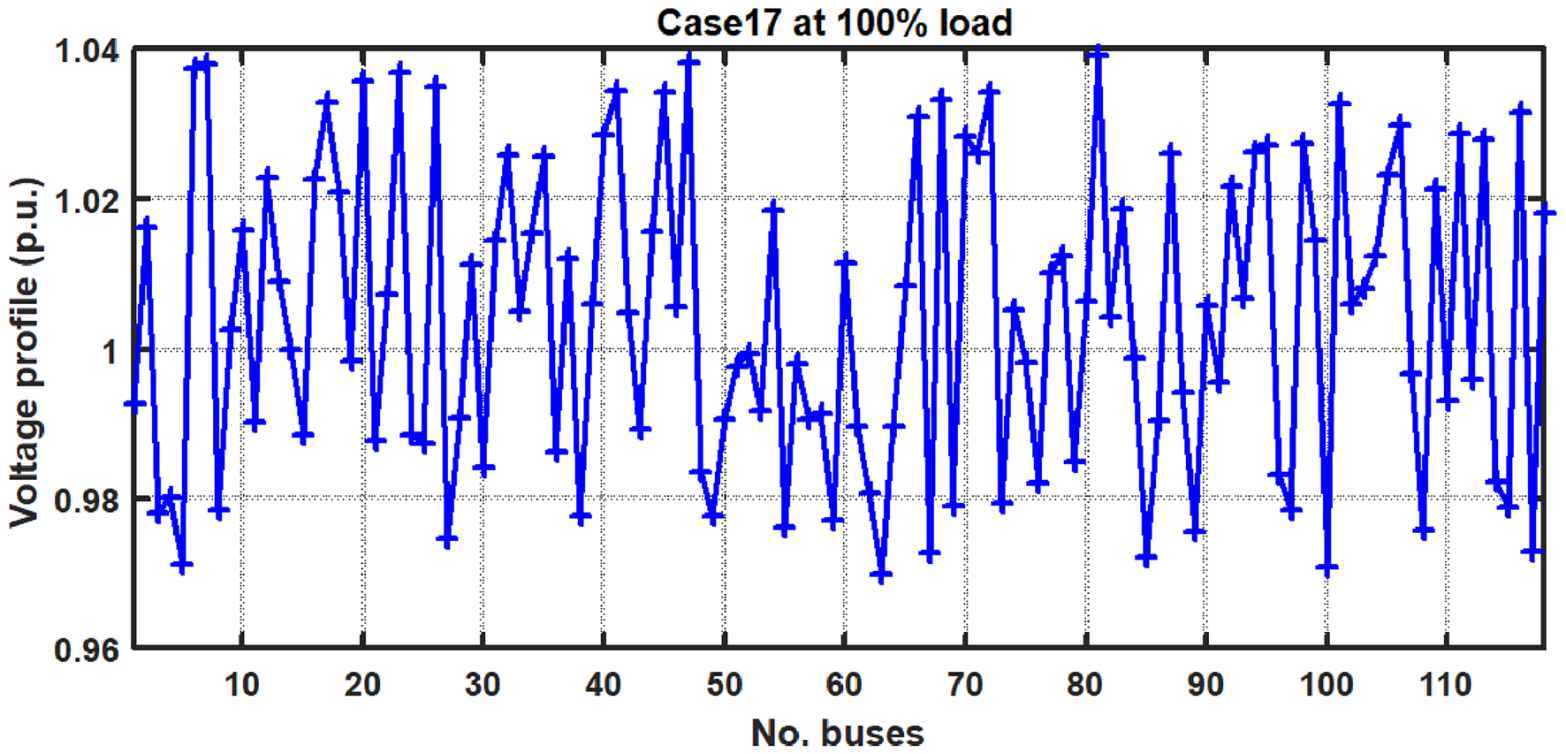
Voltage at different buses for 60% load with frequency security restriction and RES & ESS.

**Fig. 20:**
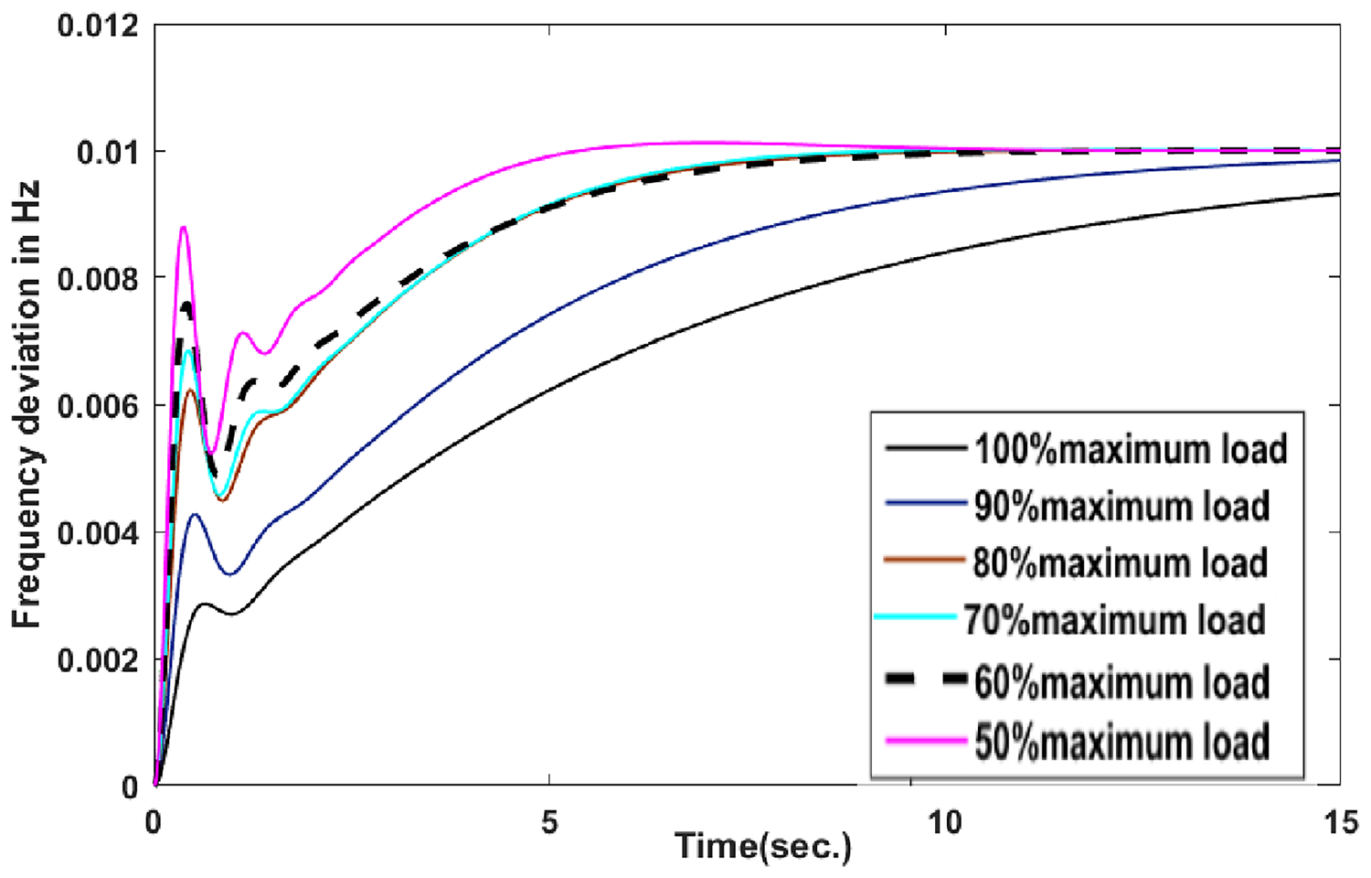
Variation in frequency at different loads without frequency security constraint.

**Fig. 21:**
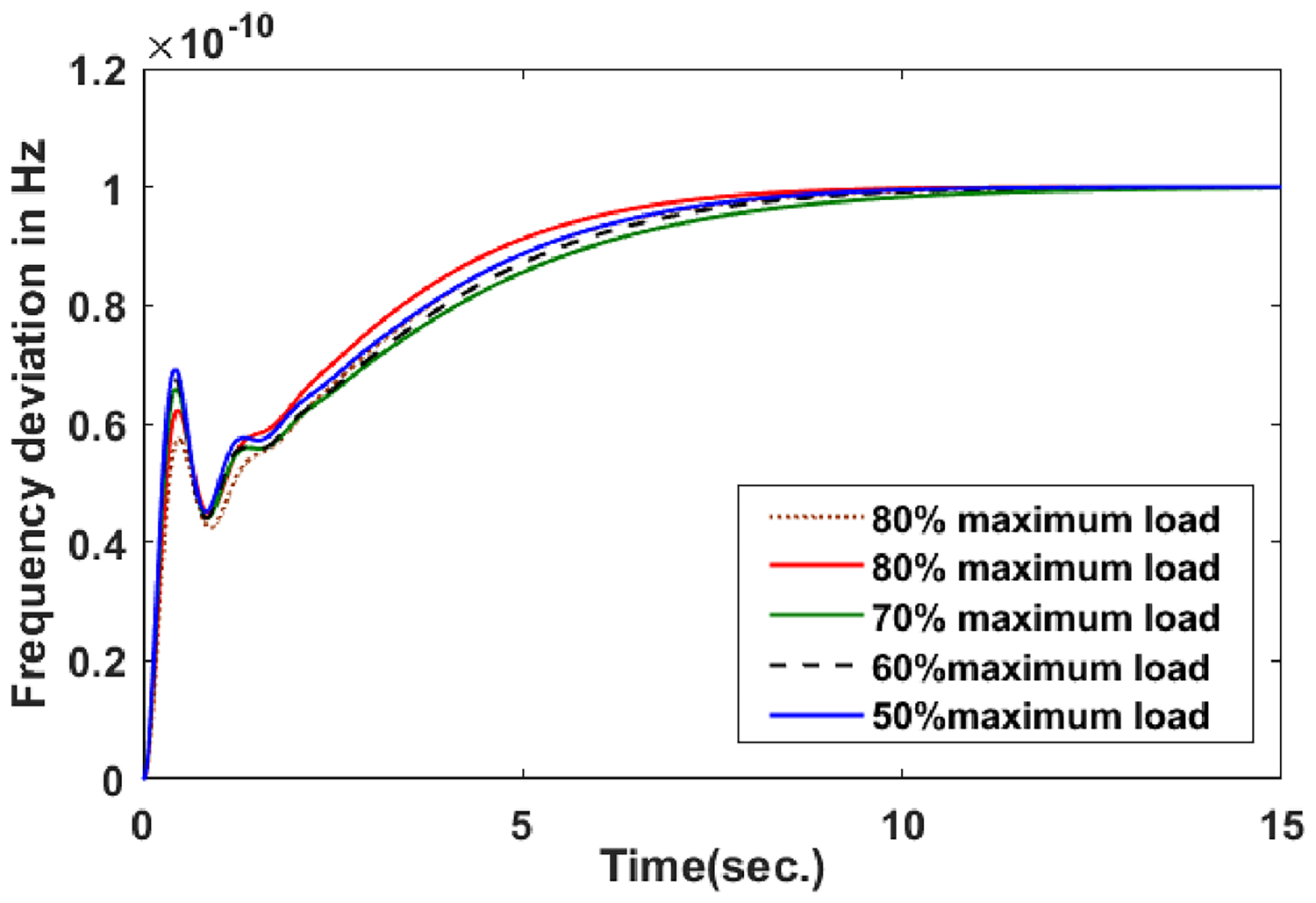
Variation in frequency at different loads with frequency security constraint.

**Fig. 22:**
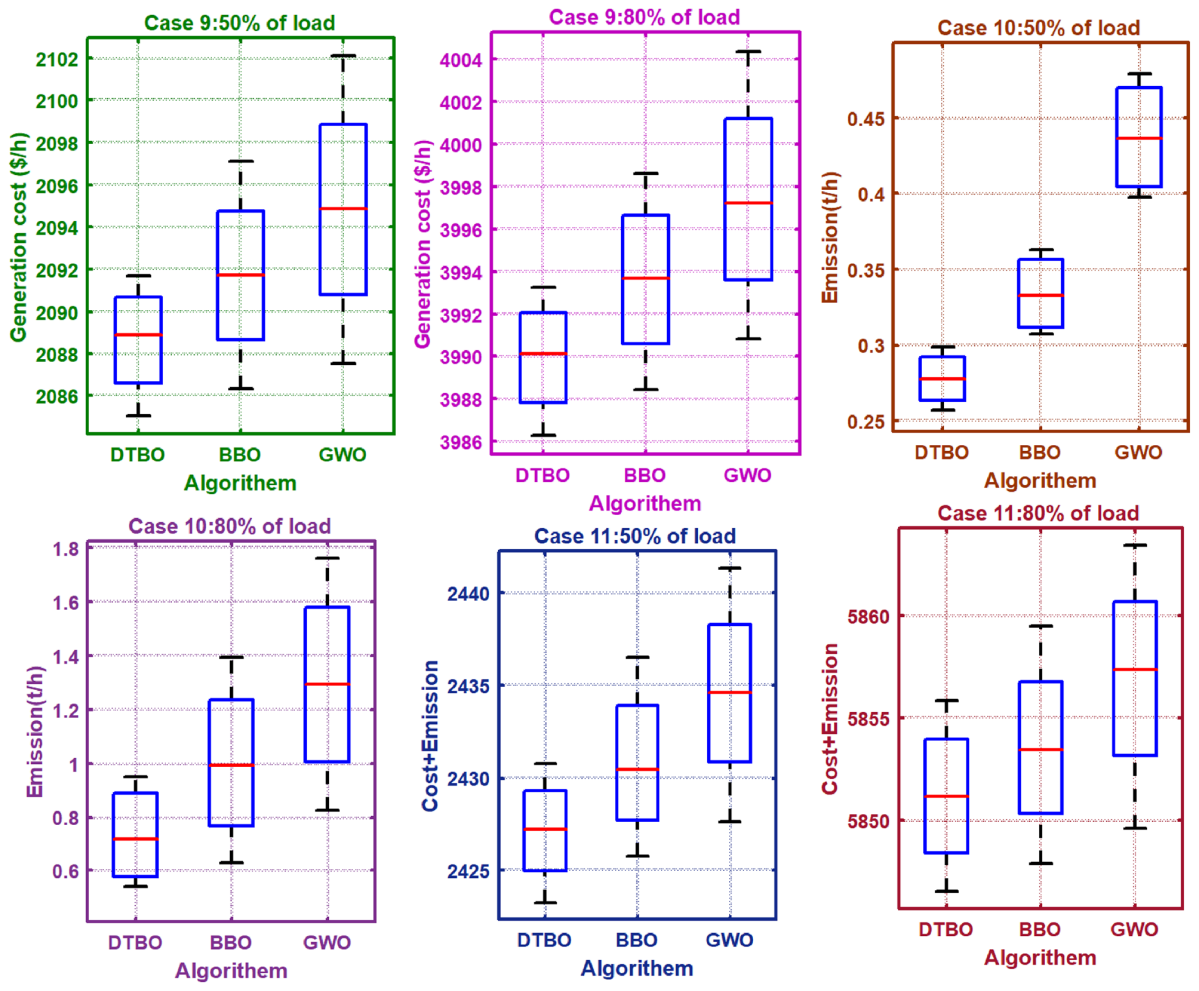
Box plot for case 9, 10 and 11 at 50% & 80% load of various techniques.

**Fig. 23:**
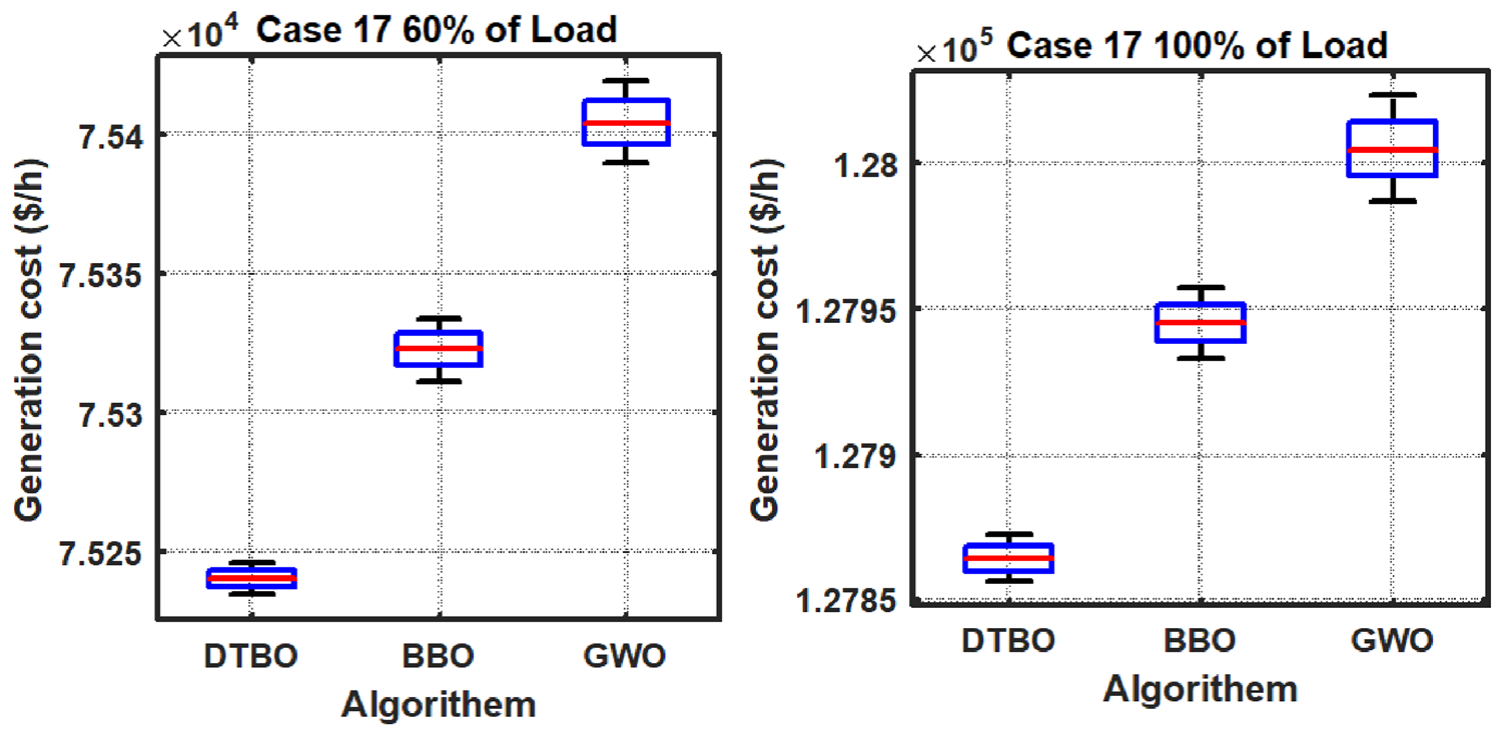
Box plot for case 17 at 60% & 100% load of different algorithm.

**Fig. 24:**
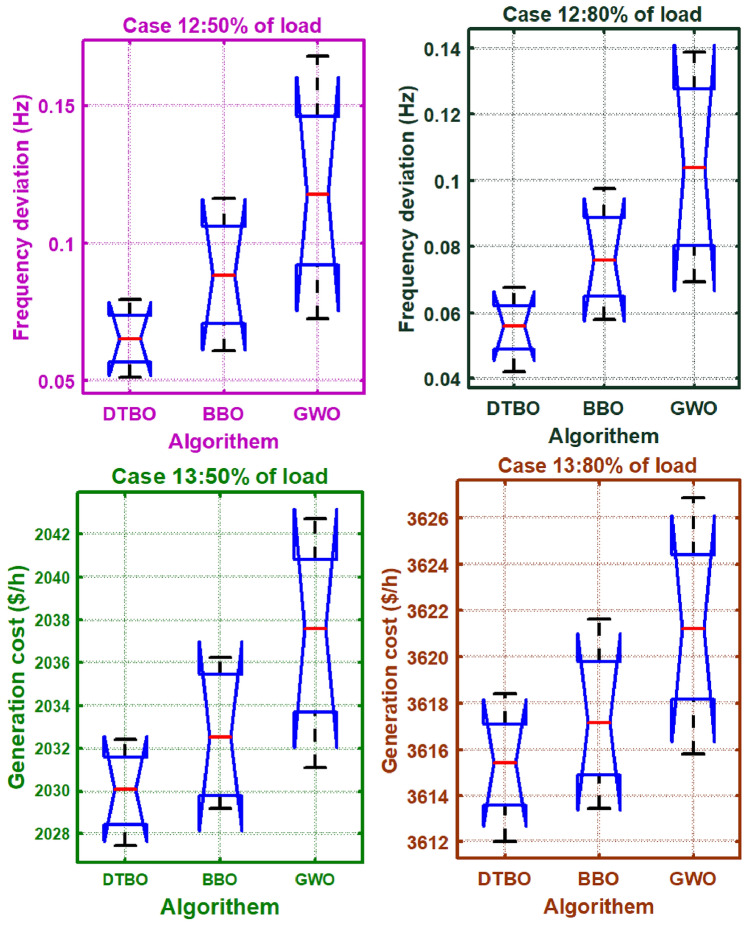
ANOVA figure for Cases 12 and 13 at 50% and 80% loads using various techniques.

**Fig. 25:**
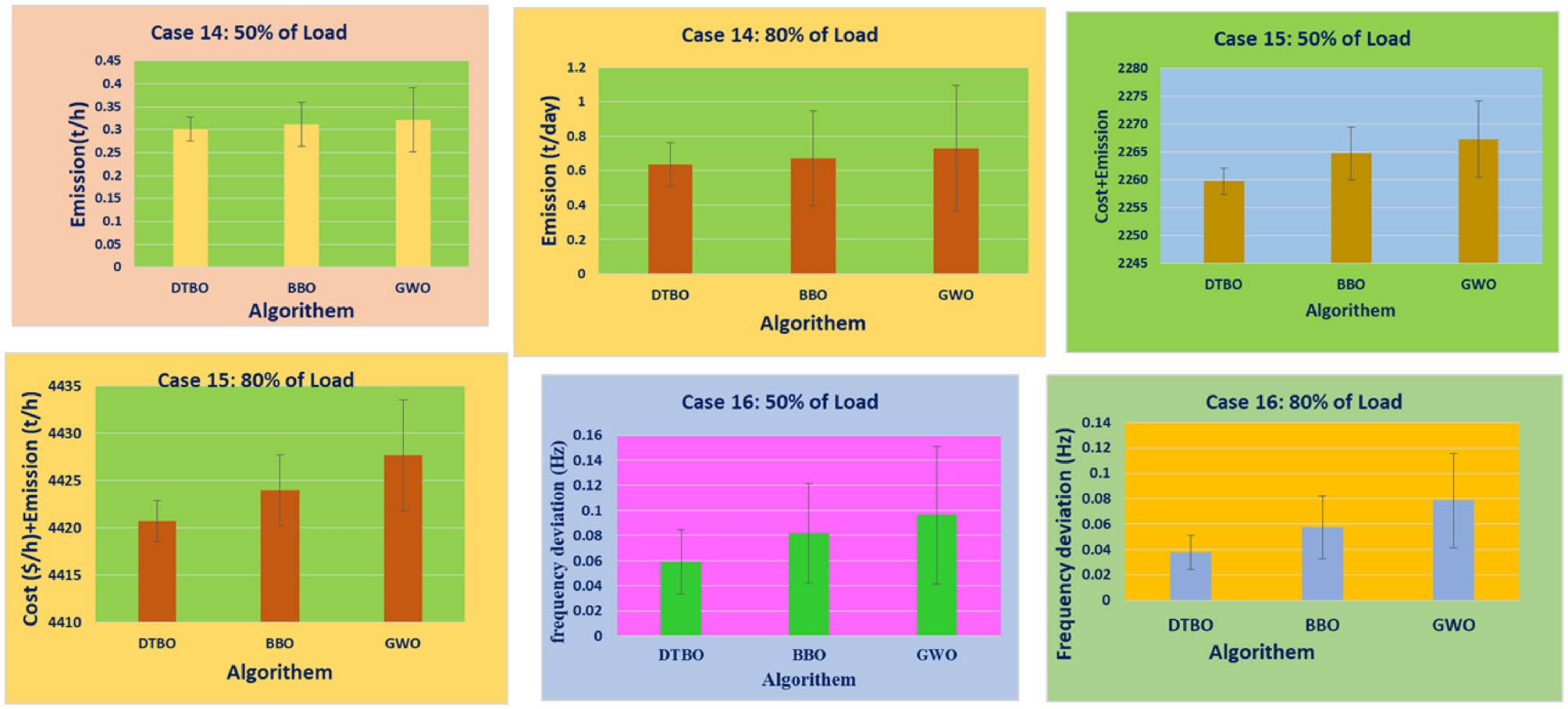
Error bar for Case 14, 15 and 16 at 50% & 80% load of various techniques.

### Two-area multi sources, thermal RES-ESS with deregulated environment

Unilateral Transaction: It is used on the two-area hybrid system in deregulated contexts to gauge the algorithm’s efficacy. All GENCOs participate equally in unilateral transactions, and the participation factor for each GENCO is determined by: ${{apf}_{11}}=0.5;$ $ap{{f}_{12}}=1-0.5=0.5;$ $ap{{f}_{21}}=0.5$; $ap{{f}_{22}}=0.5.$

Let’s investigate how area-1’s load varies by 0.1 p.u (M.w). Load disturbance occurs in $DISCO_1$ and $DISCO_2$ in the unilateral condition. The GENCOs have no demand from the $DISCO_3$ & $DISCO_4$ in this situation. The GENCOs’ coefficient of participation factor determines how much load is required by DISCOs. Here, load demand must match the agreement between DISCOs and GENCOs in order to maintain the steady state frequency. $\Delta P_{L1}$ and $\Delta P_{L2}$ are the load demands for the proposed system of $DISCO_1$ and $DISCO_2$, respectively, where the value of $\Delta P_{L1}$ is 0.1p.u (M.w). As a result, the suggested test system’s DISCO participation matrix (DPM) is provided by:

82 $$\begin{aligned} \text {DPM}=\left[ \begin{array}{llll} \text {0}\text {.5} & \text {0}\text {.5} & \text {0} & \text {0} \\ \text {0}\text {.5} & \text {0}\text {.5} & \text {0} & \text {0} \\ \text {0} & \text {0} & \text {0} & \text {0} \\ \text {0} & \text {0} & \text {0} & \text {0} \\ \end{array} \right] \end{aligned}$$

In the steady state condition, each DISCO’s demand must have been met by GENCO generation, which is given by:

83 $$\begin{aligned} \left[ \begin{array}{l} \Delta {{P}_{g1ss}} \\ \Delta {{P}_{g2ss}} \\ \Delta {{P}_{g3ss}} \\ \Delta {{P}_{g4ss}} \\ \end{array} \right] =\left[ \begin{array}{llll} 0.5 & 0.5 & 0 & 0 \\ 0.5 & 0.5 & 0 & 0 \\ 0 & 0 & 0 & 0 \\ 0 & 0 & 0 & 0 \\ \end{array} \right] \left[ \begin{array}{l} 0.1 \\ 0.1 \\ 0.0 \\ 0.0 \\ \end{array} \right] \end{aligned}$$

The suggested DTBO algorithms are utilised to optimise the values of the PID & FOPID controller. The PID controller is first utilized in the units primarily for thermal purposes and the suggested DTBO method is used to optimize its values. Afterword, DTBO tuned PID with thermal & FACTS, DTBO tuned FOPID with thermal,RES & FACTS and DTBO tuned FOPID with thermal, RES, ESS & FACTS are applied. The results show that, in comparison to other controllers, oscillation is rapidly dying out for DTBO tuned FOPID with thermal, RES, ESS, and FACTS (Tables 8, 24, Table 27).

Table 13 show the results of a comparison simulation for 50% load in unilateral condition. Furthermore, it is evident from Table 13 that the DTBO tuned FOPID with thermal, RES, ESS & FACTS produces better undershoot, overshoot, settling time, and corresponding fitness values than the others. Figure 12a–c demonstrate the dynamic response of the suggested test system utilising the DTBO algorithms for 50% load in unilateral condition. It displays the tie power error and frequency deviation in areas 1 and 2 that were acquired using the unilateral operation mode. It has been found that dynamic response produced by DTBO tuned FOPID with thermal, RES, ESS & FACTS is better than that produced by others. Using the DTBO tuned FOPID with thermal, RES, ESS & FACTS at 50% load, it is noted that the percentages of improvement in OS, US and ST for $\Delta f_1$ are 16.62%, 11.54% & 5.4% compared to DTBO tuned FOPID with thermal,RES & FACTS and 51.09%, 74.78% & 64.36% compared to DTBO tuned PID with thermal,RES & FACTS. And for $\Delta f_2$ are 19.86%, 12.84% and 10.74% compared to DTBO tuned FOPID with thermal, RES & FACTS and 27.04%, 1.43% and 31.19% compared DTBO tuned PID with thermal,RES & FACTS (Figs. 14, 15, 16, 17 and 18 and Table 23).

The numerical outcomes for 80% load of various approaches for unilateral condition are shown in Table 14. For DTBO tuned FOPID with thermal, RES, ESS & FACTS at 80% load, the overshoot, undershoot and settling times of $\Delta {{f}_{1}}$ have been reduced by 13.45%, 3.38% & 23.25%, compared to DTBO tuned FOPID with thermal,RES & FACTS and 51%, 70.7%, 66.21% compared to DTBO tuned PID with thermal,RES & FACTS. And for $\Delta {{f}_{2}}$ are improved by 14.41%, 23.5% and 6%, compared to DTBO tuned FOPID with thermal,RES & FACTS and 52.21%, 78.36%, 71.54% compared to DTBO tuned PID with thermal,RES & FACTS. Ultimately, objective function are improved by 35.46%, 83.4% using of DTBO tuned FOPID with thermal, RES, ESS & FACTS compared to DTBO tuned FOPID with thermal,RES & FACTS and DTBO tuned PID with thermal,RES & FACTS respectively. It has also been concluded from the output that suggested DTBO tuned FOPID with thermal, RES, ESS & FACTS can find a better solution compared to the other methods. Figure 12a–c demonstrate the dynamic response of the suggested test system utilising the DTBO algorithms for 50% load in unilateral condition. And Fig. 14a–c demonstrate the dynamic response of the suggested test system utilising the DTBO algorithms for 80% load in unilateral condition. From the dynamic responses of proposed DTBO tuned FOPID with thermal, RES, ESS and FACTS gives better solution compared to other techniques. It confirms superiority of proposed algorithm over BBO and GWO techniques (Figs. 19, 20 and 21).

Bilateral transactions: The aforementioned method is employed in the same test system under the case of a bilateral transaction to validate the strategy. In the instance of a bilateral transaction, DISCOs entered into contracts in both their own and other sectors. As a result, the value of the DPM matrix’s cpfs has changed.To test the tunability of various controllers under various disturbances, two alternative DPM matrices, ($DPM_1$ and $DPM_2$), have been taken into consideration in this case. The $DPM_1$ is written as follows:

84 $$\begin{aligned} \text {DPM}=\left[ \begin{array}{llll} \text {0}\text {.5} & \text {0}\text {.5} & \text {0} & \text {0} \\ \text {0}\text {.5} & \text {0}\text {.5} & \text {0} & \text {0} \\ \text {0} & \text {0} & \text {0} & \text {0} \\ \text {0} & \text {0} & \text {0} & \text {0} \\ \end{array} \right] \end{aligned}$$

$${{P}_{g1ss}}=cp{{f}_{11}} \times \Delta {{P}_{L1}}+cp{{f}_{12}} \times \Delta {{P}_{L2+}}+cp{{f}_{13}} \times \Delta {{P}_{L3}} +cp{{f}_{14}}\times \Delta {{P}_{L4}} =0.5\times 0.1+0.25\times 0.1+0.0 \times 0.1+0.3\times 0.1 =0.105 p.u(M.w).$$

Similarly,

85 $$\begin{aligned} \left. \begin{array}{ll} & \Delta {{\text {P}}_{\text {g2ss}}}=\text {0}\text {.045Pu}\ \text {Mw;} \\ & \!\!\Delta \!\!{{\text {P}}_{\text {g3ss}}}=\text {0}\text {.035Pu}\ \text {Mw;} \\ & \!\!\Delta \!\!{{\text {P}}_{\text {g4ss}}}=\text {0}\text {.11Pu}\ \text {Mw;} \\ \end{array} \right\} \end{aligned}$$

In the second instance, according to the provided DPM ($DPM_2$) matrix, DISCOs have various contracts with the GENCOs in their region as well as with the other area:

86 $$\begin{aligned} {{{DPM}_{2}}}= \left[ \begin{array}{llll} 0.3 & 0.15 & 0.4 & 0.8 \\ 0.2 & 0.25 & 0.3 & 0.0 \\ 0.4 & 0.35 & 0.2 & 0.2 \\ 0.1 & 0.25 & 0.1 & 0.0 \\ \end{array} \right] \end{aligned}$$

The steady-state value of the powers produced by each GENCO is calculated using the *DPM*2 matrix provided by (86).

87 $$\begin{aligned} \left. \begin{array}{l} \Delta \text {Pg1ss=0}\text {.165 pu(MW)} \\ \Delta \text {Pg2ss=0}\text {.075pu(MW)} \\ \Delta \text {Pg3ss=0}\text {.115pu(MW)} \\ \Delta \text {Pg4ss=0}\text {.045pu(MW)} \\ \end{array} \right\} \end{aligned}$$

**Table 23:** An overview of IEEE 118-bus System under study.

Items	Quantity	Details
Buses	118	^43^
Branches	186	^43^
Thermal generators	54	Buses: 69 (swing), 1,4,6, 8,10,12,15,18,19,24,25,26.27,31,32,34,36,40, 42,46,49,54,55,56,59,61,62,65,66,69,70,72,73, 74,76,77,80,85,87,89,90,91,92,99,100,103,104, 105,107,110,111,112,113 and 116
Wind generators (WG)	1	Bus:81
Solar PV unit (SPV)	1	Bus:64
ESS	1	Bus:117
Tap changing transformer	9	Branches: (8–5), (26–35), (30–17), (38–37), (63–59), (64–61), (65–66), (68–69) and (81–80)
Control variables	134	Generator bus real powers (57) + voltages (54) + transformer tap settings (9) + shunt capacitor (14)
Load demand		4242.0MW, 1439.0 MVAr
Range of load bus voltage	64	[0.95–1.05]p.u.
Compensation devices	14	Buses: 5, 34, 37, 44, 45, 46, 48, 74, 79, 82, 83, 105, 107 and 110

**Table 24:** Simulation findings and ideal control variable settings for Case 17 with variable loads (60% to 100%) (IEEE 118 bus system) at a certain time instance.

Control parameters	Min	Max	60$$\%$$ of load	70$$\%$$ of load	80$$\%$$ of load	90$$\%$$ of load	of load	Control parameters	Min	Max	60$$\%$$ of load	70$$\%$$ of load	80$$\%$$ of load	90$$\%$$ of load	100$$\%$$ of load
Generator power								Generator voltage							
$${{P}_{G1}}$$(MW)	0	100	13.56	15.477	27.296	36.882	33.521	V1(p.u.)	0.94	1.06	1.0284	1.041	0.9568	0.9482	1.0384
$${{P}_{G4}}$$(MW)	0	100	18.198	21.707	18.512	29.151	22.63	V4(p.u.)	0.94	1.06	1.0143	1.0469	0.948	0.9587	1.0483
$${{P}_{G6}}$$(MW)	0	100	53.748	61.768	61.456	80.685	67.86	V6(p.u.)	0.94	1.06	1.0317	1.0496	0.9793	1.0316	1.0046
$${{P}_{G8}}$$(MW)	0	100	35.526	41.244	50.816	70.785	45.79	V8(p.u.)	0.94	1.06	0.9486	1.0083	1.0317	1.0526	0.9584
$${{P}_{G10}}$$(MW)	0	550	29.61	34.937	44.984	240.804	210.91	V10(p.u.)	0.94	1.06	1.03957	1.0513	0.9474	0.9486	0.9751
$${{P}_{G12}}$$(MW)	0	185	35.844	41.293	61.448	75.834	77.74	V12(p.u.)	0.94	1.06	1.0042	1.0293	1.0416	1.0024	1.0149
$${{P}_{G15}}$$(MW)	0	100	6.672	9.009	18.76	51.066	87.58	V15(p.u.)	0.94	1.06	1.0039	0.948	1.0302	1.0247	0.9486
$${{P}_{G18}}$$(MW)	0	100	47.328	55.139	19.128	30.825	66.69	V18(p.u.)	0.94	1.06	0.9685	1.0497	1.0502	0.9496	1.0124
$${{P}_{G19}}$$(MW)	0	100	9.108	11.704	36.224	60.786	82.76	V19(p.u.)	0.94	1.06	1.0386	1.0238	1.0068	0.9385	0.9483
$${{P}_{G24}}$$(MW)	0	100	12.816	15.407	18.76	10.107	88.86	V24(p.u.)	0.94	1.06	1.0479	0.9786	1.007	0.9894	1.0279
$${{P}_{G25}}$$(MW)	0	320	160.068	186.109	161.872	178.263	144.48	V25(p.u.)	0.94	1.06	1.0069	1.0278	1.034	1.0438	0.9794
$${{P}_{G26}}$$(MW)	0	414	100.446	116.669	151.376	179.883	176.92	V26(p.u.)	0.94	1.06	0.9784	0.9874	0.979	1.0413	0.9573
$${{P}_{G27}}$$(MW)	0	100	7.23	9.737	27.4	80.784	66.81	V27(p.u.)	0.94	1.06	1.036	1.0463	1.0176	1.0258	1.0395
$${{P}_{G31}}$$(MW)	0	107	43.722	49.161	18.76	31.068	89.69	V31(p.u.)	0.94	1.06	1.0286	0.9397	1.0268	0.9794	1.0486
$${{P}_{G32}}$$(MW)	0	100	27.45	31.115	18.296	50.076	67.54	V32(p.u.)	0.94	1.06	0.9897	1.0176	1.0179	0.9374	0.9386
$${{P}_{G34}}$$(MW)	0	100	50.442	60.732	62.592	60.606	21.82	V34(p.u.)	0.94	1.06	1.0196	0.9385	0.9586	1.0493	0.9684
$${{P}_{G36}}$$(MW)	0	100	46.206	53.907	53.792	80.784	67.29	V36(p.u.)	0.94	1.06	1.0397	1.0401	0.9486	0.9675	1.0568
$${{P}_{G40}}$$(MW)	0	100	24.732	29.561	53.904	20.079	66.68	V40(p.u.)	0.94	1.06	1.0038	0.9697	0.9658	0.9876	1.0098
$${{P}_{G42}}$$(MW)	0	100	46.248	55.132	36.576	27.072	41.14	V42(p.u.)	0.94	1.06	0.9867	0.948	1.048	0.9879	1.0486
$${{P}_{G46}}$$(MW)	0	119	55.848	64.792	63.136	45.198	22.64	V46(p.u.)	0.94	1.06	1.0413	0.9796	1.0276	0.984	1.0537
$${{P}_{G49}}$$(MW)	0	304	61.158	77.56	116.208	89.883	47.75	V49(p.u.)	0.94	1.06	1.049	0.9586	1.0401	1.0059	0.947
$${{P}_{G54}}$$(MW)	0	148	30.996	34.993	62.896	50.787	44.68	V54(p.u.)	0.94	1.06	1.0338	0.9574	0.9785	0.9768	1.0387
$${{P}_{G55}}$$(MW)	0	100	21.228	25.718	18.728	10.701	34.32	V55(p.u.)	0.94	1.06	0.9387	1.0491	0.9384	1.0386	0.9758
$${{P}_{G56}}$$(MW)	0	100	5.67	7.469	17.072	60.786	71.67	V56(p.u.)	0.94	1.06	1.0386	1.0485	1.0396	0.938	0.949
$${{P}_{G59}}$$(MW)	0	255	33.054	38.192	8.984	50.787	34.79	V59(p.u.)	0.94	1.06	1.0124	0.955	0.958	1.0019	0.948
$${{P}_{G61}}$$(MW)	0	270	99.03	117.355	163.616	240.786	218.76	V61(p.u.)	0.94	1.06	1.0169	1.0209	1.0438	0.9768	1.0339
$${{P}_{G62}}$$(MW)	0	100	53.802	62.209	70.08	10.107	88.69	V62(p.u.)	0.94	1.06	0.9686	1.0069	1.0138	0.9684	0.9573
$${{P}_{G65}}$$(MW)	0	491	98.742	114.394	149.808	150.786	155.41	V65(p.u.)	0.94	1.06	1.0491	1.0048	0.9683	1.0478	0.9685
$${{P}_{G66}}$$(MW)	0	492	221.052	257.418	281.848	276.885	355.46	V66(p.u.)	0.94	1.06	1.0106	1.0587	1.0569	0.9386	1.0314
$${{P}_{G69}}$$(MW)	0	300	72.486	86.394	213.792	200.853	202.52	V69(p.u.)	0.94	1.06	0.9386	0.9376	0.9387	1.0038	1.0384
$${{P}_{G70}}$$(MW)	0	100	41.358	45.171	24.096	80.784	45.41	V70(p.u.)	0.94	1.06	0.9517	0.9548	1.0486	0.9786	1.0286
$${{P}_{G72}}$$(MW)	0	100	6.594	7.861	18.224	27.396	88.47	V72(p.u.)	0.94	1.06	1.0396	0.9874	1.0304	1.0368	0.9572
$${{P}_{G73}}$$(MW)	0	100	9.642	13.279	27.32	50.787	75.86	V73(p.u.)	0.94	1.06	0.9874	1.019	0.9386	1.0194	0.9379
$${{P}_{G74}}$$(MW)	0	100	59.952	62.979	45.376	20.583	45.47	V74(p.u.)	0.94	1.06	1.0258	1.0349	1.0337	1.0174	1.0178
$${{P}_{G76}}$$(MW)	0	100	49.866	59.948	37.456	35.883	48.45	V76(p.u.)	0.94	1.06	1.0279	0.9617	1.0496	0.9684	1.0297
$${{P}_{G77}}$$(MW)	0	100	50.364	59.171	79.896	80.163	29.24	V77(p.u.)	0.94	1.06	0.9875	0.9684	0.9874	1.0247	1.0059
$${{P}_{G80}}$$(MW)	0	577	53.454	61.502	63.136	50.427	31.84	V80(p.u.)	0.94	1.06	1.0326	1.0331	0.9638	0.9858	0.9364
$${{P}_{G85}}$$(MW)	0	100	32.748	39.725	70.016	69.606	67.27	V85(p.u.)	0.94	1.06	1.0304	0.9647	0.9375	0.976	1.053
$${{P}_{G87}}$$(MW)	0	104	37.308	42.686	18.76	41.391	33.74	V87(p.u.)	0.94	1.06	1.0374	1.0069	0.9784	1.0265	0.9561
$${{P}_{G89}}$$(MW)	0	707	123.204	147.609	231.76	190.782	204.54	V89(p.u.)	0.94	1.06	0.9467	1.0196	1.0242	0.9683	1.0241
$${{P}_{G90}}$$(MW)	0	100	21.96	16.436	54.032	40.788	77.91	V90(p.u.)	0.94	1.06	1.0239	0.9473	1.021	1.0438	1.0257
$${{P}_{G91}}$$(MW)	0	100	35.442	45.801	31.856	51.066	86.72	V91(p.u.)	0.94	1.06	1.0086	1.0379	1.004	0.9368	1.0204
$${{P}_{G92}}$$(MW)	0	100	11.568	12.523	16.728	35.883	45.41	V92(p.u.)	0.94	1.06	1.0196	0.9587	0.9701	0.9573	1.0207
$${{P}_{G99}}$$(MW)	0	100	56.016	62.972	54.256	31.383	22.79	V99(p.u.)	0.94	1.06	1.0384	1.0473	1.0396	0.9572	1.0486
$${{P}_{G100}}$$(MW)	0	352	54.618	66.948	62.68	20.484	19.26	V100(p.u.)	0.94	1.06	0.9759	0.9571	1.0268	1.0295	1.0068
$${{P}_{G103}}$$(MW)	0	140	56.364	63.161	61.456	51.246	33.26	V103(p.u.)	0.94	1.06	0.9461	0.9857	0.9764	1.0031	0.9864
$${{P}_{G104}}$$(MW)	0	100	9.366	10.171	18.728	30.483	88.65	V104(p.u.)	0.94	1.06	1.0243	0.9374	0.9417	1.0391	1.0069
$${{P}_{G105}}$$(MW)	0	100	22.194	25.893	45.024	21.105	56.84	V105(p.u.)	0.94	1.06	0.9684	1.0204	1.0079	1.0097	0.938
$${{P}_{G107}}$$(MW)	0	100	13.524	14.805	9.4976	26.883	31.85	V107(p.u.)	0.94	1.06	0.9674	1.0205	0.9703	1.0495	1.0258
$${{P}_{G110}}$$(MW)	0	100	43.518	44.786	45.016	39.888	19.79	V110(p.u.)	0.94	1.06	1.0206	1.0168	0.9483	1.0495	0.983
$${{P}_{G111}}$$(MW)	0	136	24.648	28.049	27.024	25.884	11.68	V111(p.u.)	0.94	1.06	0.9816	1.0207	1.0294	1.0079	1.038
$${{P}_{G112}}$$(MW)	0	100	38.01	43.386	53.824	42.795	9.74	V112(p.u.)	0.94	1.06	1.0037	0.9368	1.0386	1.0296	0.9753
$${{P}_{G113}}$$(MW)	0	100	53.688	54.838	71.776	62.883	80.17	V113(p.u.)	0.94	1.06	1.0153	1.0287	1.0131	1.0027	0.9635
$${{P}_{G116}}$$(MW)	0	100	44.64	51.38	36.496	10.107	78.58	V116(p.u.)	0.94	1.06	1.0137	0.9746	1.0094	1.0286	1.0157
PW81(MW)	0	100	47.388	62.426	62.28	69.804	79.83	Capacitor bank							
PPV64 (MW)	0	100	52.59	67.585	70.12	60.786	87.74	QC5 (MVAr)	− 40	0	− 28.78	− 7.83	− 14.46	− 37.13	− 3.86
ESS117 (MW)	0	50	15	17.5	20	22.5	24.6	QC34 (MVAr)	0	14	9.74	3.86	10.79	2.31	13.174
Transformer tap ratio								QC37(MVAr)	− 15	0	− 7.47	− 0.028	− 8.24	− 1.057	− 11.593
$${{T}_{8}}$$(p.u.)	0.9	1.1	1.048	1.069	0.9203	1.0658	1.0347	QC44(MVAr)	0	10	7.74	8.31	5.747	5.738	5.38
$${{T}_{32}}$$(p.u.)	0.9	1.1	0.9654	0.9196	0.9472	1.0591	0.9206	QC45(MVAr)	0	10	2.86	9.14	8.857	5.24	9.865
$${{T}_{36}}$$(p.u.)	0.9	1.1	0.9476	1.0013	0.9269	0.986	1.0207	QC46(MVAr)	0	10	0.47	5.205	4.863	8.68	0.79
$${{T}_{51}}$$(p.u.)	0.9	1.1	1.0204	1.0087	0.9274	1.0196	1.0284	QC48(MVAr)	0	10	6.38	0.36	3.79	2.76	4.196
$${{T}_{93}}$$(p.u.)	0.9	1.1	0.9473	0.9384	1.0298	1.0763	1.0205	QC74(MVAr)	0	12	2.04	2.058	4.206	2.586	0.317
$${{T}_{95}}$$(p.u.)	0.9	1.1	1.0206	0.9238	0.9571	1.0285	1.0473	QC79(MVAr)	0	20	2.79	16.57	7.68	19.47	11.24
$${{T}_{102}}$$(p.u.)	0.9	1.1	1.0204	1.0196	0.918	0.9074	0.9186	QC82(MVAr)	0	20	17.52	13.86	19.06	17.19	15.58
$${{T}_{107}}$$(p.u.)	0.9	1.1	0.9758	1.0251	1.0291	0.9374	0.9857	QC83(MVAr)	0	10	6.09	7.04	8.68	8.35	9.37
$${{T}_{127}}$$(p.u.)	0.9	1.1	0.9205	0.9754	1.0782	1.0114	1.0796	QC105(MVAr)	0	20	16.61	9.25	8.86	0.51	13.42
								QC107(MVAr)	0	6	2.41	5.87	1.32	5.864	4.58
								QC110(MVAr)	0	6	1.304	3.283	2.021	2.68	2.69
Objective function															
Fitness parameters						60% of load		70% of load		80% of load		90% of load		100% of load	
	Thermal:					74,784.452		90,209.453		103,910.409		117,120.231		127,320.209	
	Wind:					202.46		215.94		201.13		199.74		206.47	
	Solar:					214.64		223.437		218.37		193.46		218.16	
	ESS:					32.625		79.28		102.386		88.516		111.68	
cost ($/day)	Total generation					75,234.177		90,728.11		104,432.295		117,601.947		127,856.519	
Emission (t/hr):						2.164		1.361		1.768		2.107		1.698	
Ploss (MW)						41.844		49.784		52.564		57.069		93.15	
VD(p.u.)						0.6223		0.71252		0.7327		0.7988		0.81137	
L-index						0.3448		0.322		0.3587		0.372		0.398

**Table 25:** Comparing the suggested DTBO statistically with BWM_HS, CVnew, SGSADE, HGSO, LSHADE-cnEpSin, and LSHADE-SPACMA on CEC 2017 using 30D $F1-F16$.

CEC 2017 (D = 30)								
						LSHADE	LSHADE	
Function		BWM_HS	CVnew	SGSADE	HGSO	-cnEpSin	-SPACMA	DTBO
Unimodal								
	Mean	3.783E+03	1.301E+10	3.496E−08	5.489E+03	0.000E+00	2.897E−08	3.479E−08
F1	SD	4.912E+03	0.000E+00	3.865E−08	1.214E+03	0.000E+00	2.052E−08	2.041E−08
	Sign	+	+	−	−	−	−	
	Mean	1.304E−07	1.496E+02	1.417E+02	5.863E+02	2.201E−08	3.418E−08	2.024E−07
F3	SD	4.486E−08	9.503E+01	1.275E+02	2.796E+02	2.318E−08	2.049E−08	2.216E−07
	Sign	+	+	+	+	+	+	
Multi-modal								
	Mean	6.793E+01	1.486E+01	1.531E+01	4.684E+02	4.474E+01	3.326E−08	2.792E−08
F4	SD	3.069E+01	2.784E+01	2.714E+01	3.037E+02	2.876E+00	2.516E−08	1.374E−08
	Sign	+	+	+	+	+	−	
	Mean	5.037E+01	1.416E+02	8.795E+01	6.328E+02	1.547E+01	3.693E+00	3.072E+01
F5	SD	1.913E+01	2.685E+01	1.806E+01	9.857E+00	2.518E+00	2.728E+00	1.023E+01
	Sign	+	+	+	+	−	−	
	Mean	1.327E−05	2.216E+01	2.326E−08	5.895E+02	1.106E−08	1.423E−08	8.254E+00
F6	SD	2.217E−05	8.264E+00	1.497E−08	7.594E+00	1.538E−08	1.417E−08	1.019E−07
	Sign	−	+	−	+	−	−	
	Mean	5.879E+01	2.427E+02	1.415E+02	8.538E+02	4.864E+01	3.496E+01	5.857E+00
F7	SD	9.584E+00	2.273E+01	1.586E+01	6.372E+01	2.328E+00	8.316E−01	5.462E−01
	Sign	+	+	+	+	+	+	
	Mean	4.874E+01	1.318E+02	8.415E+01	8.317E+02	1.296E+01	3.683E+00	3.374E+00
F8	SD	1.318E+01	2.704E+01	1.476E+01	2.486E+01	2.785E+00	1.693E+00	2.486E+00
	Sign	+	+	+	+	+	=	
	Mean	1.201E+01	2.184E+03	5.869E−08	1.817E+03	0.317E+00	0.418E+00	0.000E+00
F9	SD	8.0031E+01	8.526E+02	6.024E−08	2.429E+02	0.326E+00	0.714E+00	5.318E−08E+00
	Sign	+	+	+	+	+	+	
	Mean	2.684E+03	4.496E+03	5.216E+03	5.319E+03	1.174E+03	1.796E+03	4.011E+02
F10	SD	4.684E+02	3.034E+02	5.486E+02	3.214E+02	2.518E+02	3.637E+02	8.875E+01
	Sign	+	+	+	+	+	+	
	Mean	9.538E+01	3.628E+01	5.045E+01	1.616E+03	1.01E+01	4.302E+00	3.527E+00
F11	SD	3.243E+01	1.828E+01	3.212E+01	2.784E+01	2.022E+01	3.592E+00	1.715E+00
	Sign	+	+	+	+	+	=	
	Mean	5.033E+05	5.212E+09	1.793E+04	5.055E+04	4.327E+02	4.897E+02	4.873E+00
F12	SD	4.582E+05	5.838E+09	6.864E+03	3.242E+04	1.532E+02	2.813E+02	4.032E+00
	Sign	+	+	+	+	+	+	
	Mean	1.782E+04	7.865E+01	2.863E+02	5.516E+04	2.317E+01	0.878E+01	7.412E−01
F13	SD	2.312E+04	2.767E+01	3.032E+02	2.214E+03	0.898E+01	5.033E+00	4.076E−01
	Sign	+	+	+	+	+	+	
	Mean	4.033E+03	5.046E+01	6.251E+01	2.401E+03	1.878E+01	2.823E+01	3.213E−01
F14	SD	3.362E+03	7.248E+00	8.763E+00	1.678E+00	2.523E+00	2.242E+00	0.732E−01
	Sign	+	+	+	+	+	+	
	Mean	8.024E+03	3.785E+01	4.872E+01	3.834E+03	4.018E+00	4.547E+00	4.248E+01
F15	SD	8.796E+03	8.802E+00	3.024E+01	5.017E+02	2.049E+00	2.872E+00	1.424E+01
	Sign	+	=	+	+	−	−	
	Mean	4.872E+02	7.496E+02	5.074E+02	3.412E+03	2.732E+01	4.363E+01	5.742E+00
F16	SD	1.868E+02	2.035E+02	1.858E+02	3.532E+02	2.864E+01	5.684E+01	3.064E+00
	Sign	+	+	+	+	+	+

**Table 26:** Statistical comparison of the suggested DTBO on CEC 2017 with 30D taking into account BWM_HS, CVnew, SGSADE, HGSO, LSHADE-cnEpSin, and LSHADE-SPACMA $F17-F30$.

CEC-2017 (D = 30)								
						LSHADE	LSHADE	
Function		BWM_HS	CVnew	SGSADE	HGSO	-cnEpSin	-SPACMA	DTBO
Hybrid								
	Mean	3.243E+02	2.035E+02	8.264E+01	2.013E+03	3.312E+01	2.878E+01	1.862E+01
F17	SD	1.857E+02	6.757E+01	2.352E+01	1.847E+01	4.877E+00	7.418E+00	1.079E+01
	Sign	+	+	−	+	−	−	
	Mean	1.523E+05	4.024E+01	1.876E+03	0.867E+04	1.852E+01	3.695E+01	1.802E+03
F18	SD	5.796E+04	6.863E+00	1.856E+03	5.785E+04	6.742E−01	2.013E+00	1.827E-01
	Sign	+	−	=	+	−	−	
	Mean	7.764E+03	1.804E+01	2.317E+01	1.868E+03	4.523E+00	8.268E+00	7.482E−01
F19	SD	9.792E+03	3.085E+00	6.313E+00	2.763E+03	1.869E+00	2.242E+00	6.173E+00
	Sign	+	+	+	+	+	+	
	Mean	1.792E+02	1.686E+02	0.793E+02	1.765E+03	2.516E+01	7.686E+01	3.242E+02
F20	SD	8.799E+01	9.472E+01	4.768E+01	2.867E+02	6.382E+00	4.262E+01	2.031E+01
	Sign	+	+	+	+	=	+	
	Mean	2.606E+02	1.685E+02	2.696E+02	2.855E+03	1.872E+02	1.794E+02	6.122E+00
F21	SD	1.516E+01	2.708E+01	2.313E+01	2.492E+01	2.689E+00	3.392E+00	1.024E+00
	Sign	+	+	+	+	+	+	
	Mean	1.876E+03	1.234E+03	1.765E+02	3.971E+03	2.888E+02	2.592E+02	1.267E+01
F22	SD	1.581E+03	1.754E+03	1.363E+01	8.410E+02	1.493E+01	2.754E+01	8.402E+00
	Sign	+	+	=	+	=	=	
Composite								
	Mean	4.031E+02	3.696E+02	3.792E+02	1.682E+03	2.718E+02	2.262E+02	4.039E+01
F23	SD	4.869E+01	4.758E+00	2.683E+01	5.514E+01	2.876E+01	3.503E+01	1.207E+00
	Sign	+	+	+	+	+	+	
	Mean	5.036E+02	4.538E+02	3.247E+04	2.241E+03	4.262E+02	1.767E+01	2.535E+02
F24	SD	2.368E+01	2.494E+02	2.313E+01	8.595E+01	2.513E+00	1.585E+00	3.698E+01
	Sign	+	+	+	+	+	+	
	Mean	3.794E+02	3.646E+02	4.242E+02	2.868E+02	2.422E+02	1.767E+01	1.798E+01
F25	SD	2.467E+00	7.354E-01	4.749E+00	2.864E+01	7.424E−03	1.688E−02	1.636E−03
	Sign	+	+	+	+	+	+	
	Mean	2.585E+03	3.578E+02	2.796E+03	4.755E+03	9.341E+02	9.481E+02	1.257E+02
F26	SD	6.465E+02	3.243E+01	2.041E+02	1.867E+02	4.754E+01	3.610E+01	3.037E+01
	Sign	+	+	+	+	+	+	
	Mean	5.483E+02	5.364E+02	5.492E+02	3.714E+03	5.207E+02	5.311E+02	4.317E+02
F27	SD	1.422E+01	9.797E+00	1.816E+00	1.212E+02	6.608E+00	1.743E+01	1.735E+00
	Sign	=	=	=	+	=	=	
	Mean	4.525E+02	3.358E+02	3.494E+02	3.248E+03	2.746E+02	2.863E+02	8.745E+01
F28	SD	6.523E+01	3.796E+01	5.242E+01	7.535E+01	3.892E+01	5.805E+01	3.364E+01
	Sign	+	+	+	+	+	+	
	Mean	5.244E+02	8.412E+02	6.392E+02	3.816E+03	4.426E+02	3.754E+02	6.871E+02
F29	SD	1.685E+02	1.316E+02	6.493E+01	1.536E+02	7.248E+00	4.026E+01	1.314E+02
	Sign	+	+	+	+	+	+	
	Mean	1.031E+04	2.412E+03	2.583E+03	9.685E+03	1.535E+03	8.684E+02	8.314E+02
F30	SD	5.683E+03	5.243E+02	9.478E+02	3.602E+03	4.282E+03	9.243E+02	2.801E+02
	Sign	=	−	−	=	−	−

**Table 27:** The Friedman rank test and Wilcoxon signed−rank test results, taking into account the average error value for CEC 2017 (D = 50).

Sign	DTBO	Vs.	BWM_HS	CVnew	SGSADE	HGSO	LSHADE-cnEpSin	LSHADE-SPACMA
+/=/−			27/00/02	22/02/05	26/00/03	28/00/01	17/04/08	18/03/08
Statistical rank		BWM_HS	CVnew	SGSADE	HGSO	LSHADE-cnEpSin	LSHADE-SPACMA	CODTBO
Friedman rank		5.716	4.783	5.247	7.218	2.785	2.397	1.518
Overall rank		6	4	5	7	3	2	1

The results of a comparison for 50% load in bilateral condition are displayed in Table 13. It is evident from Table 13 that the DTBO tuned FOPID with thermal, RES, ESS & FACTS produces better undershoot, overshoot, settling time and corresponding fitness values than the others. Figure 12d–f demonstrate the dynamic response of the suggested test system utilising the DTBO algorithms for 50% load in bilateral condition. It displays the tie power error and frequency deviation in areas 1 and 2 that were acquired using the bilateral operation mode. It has been found that dynamic response produced by DTBO tuned FOPID with thermal, RES, ESS & FACTS is better than that produced by others. Using the DTBO tuned FOPID with thermal, RES, ESS & FACTS at 50% load, it is noted that the percentages of improvement in OS, US and ST for $\Delta f_1$ are 71.5%, 14.12% & 13.13% compared to DTBO tuned FOPID with thermal, RES & FACTS and 94.1%, 25.46% & 41.87% compared to DTBO tuned PID with thermal, RES & FACTS. And for $\Delta f_2$ are 14%, 12.9% and 26.97% compared to DTBO tuned FOPID with thermal, RES & FACTS and 49.84%, 70.62% and 66.19% compared DTBO tuned PID with thermal, RES & FACTS.

The numerical outcomes for 80% load of various approaches for bilateral condition are shown in Table 14. For DTBO tuned FOPID with thermal, RES, ESS & FACTS at 80% load, the overshoot, undershoot and settling times of $\Delta {{f}_{1}}$ have been reduced by 14.87%, 9.4% & 9.87%, compared to DTBO tuned FOPID with thermal, RES & FACTS and 48.99%, 69.86%, 64.5% compared to DTBO tuned PID with thermal, RES & FACTS. And for $\Delta {{f}_{2}}$ are improved by 46.82%, 18.91% and 12.18%, compared to DTBO tuned FOPID with thermal, RES & FACTS and 99.1%, 51.64%, 61.82% compared to DTBO tuned PID with thermal,RES & FACTS. Ultimately, objective function are improved by 35.32%, 85.62% using of DTBO tuned FOPID with thermal, RES, ESS & FACTS compared to DTBO tuned FOPID with thermal, RES & FACTS and DTBO tuned PID with thermal, RES & FACTS respectively. It has also been concluded from the output that suggested DTBO tuned FOPID with thermal, RES, ESS & FACTS can find a better solution compared to the other methods. The coordinated controller reduces the peak overshoot, undershoot, and settling time of the system responses, as can be observed in Fig. 12d–f for 50% load and in Fig. 14d–f for 80% load. It can be concluded that, in comparison to other approaches such as DTBO tuned PID, BBO tuned PID and GWO tuned PID controller, the suggested DTBO tuned FOPID exhibits superior dynamic behavior and offers improved overshoot, undershoot and settling time. This can assist in rapidly restoring the frequency and tie-line power to their stable levels and improving the stability margin appropriately. This amplifies the point that DTBO can obtain solutions of higher quality (Figs. 22, 23).

### CEC benchmark system

The IEEE CEC Benchmark System includes a range of benchmark functions designed to evaluate the performance and behavior of various multi-objective combinatorial optimization tasks (MCTs). These functions are used to assess the MCTs’ ability to explore different solutions, intensify toward optimum solutions, and effectively converge. The IEEE CEC Benchmark System can be configured in 10D, 30D, 50D, and 100D dimensions. However, in this work, explicitly investigate the IEEE CEC 2017 benchmark system using 30D and 50D dimensions. The IEEE CEC 2017 benchmark system includes many functions that are classified as unimodal, multi-modal, hybrid, and composite. These functions are derived from^44^. Unimodal functions are used to assess the optimization process’s capacity to intensify toward a single optimal solution. Multi-modal functions evaluate the algorithm’s ability to investigate several solutions. Hybrid functions combine unimodal and multimodal properties. Composite functions are created by combining two or more unimodal and multimodal functions. For each experiment function in both IEEE CEC benchmark systems, we set a maximum limit of function assessments at ${{10}^{4}}\times D$. We thoroughly evaluate the algorithm’s performance through 30 distinct runs. As mentioned earlier, the benchmark system’s test functions can be categorized into different groups: $F1-F3$, $F4-F16$, $F17-F22$, and $F23-F30$ are unimodal, multimodal, hybrid, and composite functions, respectively. It is important to note that F2 is not included in the IEEE CEC 2017 benchmark system because of its unstable characteristics, as stated in^44^ (Figs. 24, 25 and Table 24)

#### CEC 2017 (30D)

Table 25 presents statistical results that show the optimal mean error values and standard deviations (SD) obtained by the proposed DTBO and other MCTs for both unimodal and multimodal benchmark functions in the context of 30 dimensions (30D). It is important to note that mean error values less than 10e−08 are considered to be 0 for all participating MCTs. For the majority of the test functions, Table 25 clearly shows that our proposed MCT outperforms the majority of the other cutting-edge MCTs used in this work in terms of mean error values. This improved performance in obtaining optimal values for unimodal and multimodal test functions shows that the modifications we have made to our proposed MCT have successfully increased its capacity for intensification and diversification when compared to the other MCTs under consideration. Furthermore, it is evident from examining the SD values in Table 25 that, of all the MCTs considered, the proposed DTBO has the highest level of precision. Table 26 compares the best mean error values and SD generated by different MCTs for hybrid and composite functions. The results in Table 26 show that the proposed DTBO outperforms the other MCTs in the experiment in terms of mean error values and SD, suggesting its potential to yield highly accurate and superior solutions. A Wilcoxon signed-rank test with a significance threshold of 0.05 is used to compare the mean error values of the recommended MCT with the other MCTs for each test function in order to assess the statistical significance^45^. Based on the signed-rank test findings, the competing MCTs are allocated “+”, “=”, and “−” signs according to how well they perform statistically against the proposed DTBO. The “+”, “=”, and “−” indications denote whether the performance of an MCT is inferior to, equal to, or superior than the suggested DTBO. The competing MCTs are assigned “+”, “=”, and “−” signs signs based on their statistical performance versus the proposed DTBO, as determined by the signed-rank test results. The symbols “+”, “=”, and “−” indicate whether an MCT’s performance is better than, equal to, or worse than the recommended DTBO. Table 26, which demonstrates that the proposed MCT obtains the most “+” signs compared to other participating MCTs, confirms the statistical robustness of the proposed DTBO over its competitors. The Friedman rank test^45^ is used to further assess the overall statistical performance of the proposed MCT. Based on the Friedman rank, the recommended DTBO is ranked highest among all the MCTs that are examined (Table 27).

### Statistical assessment

Tables 20 and 21 demonstrate that the statistical values obtained from the DTBO results are superior to the BBO and GWO findings. It leads to the conclusion that performance with frequency constraints is superior to that without frequency limitations in the OPF problem. An ANOVA is a statistical method used to examine the mean of various strategies created for each experiment where a significant difference is found. If the P-value is smaller than 0.05%, there is a substantial difference between the recommended and alternative procedures. In this case, the significance level is 5%. The superiority of DTBO over alternative methods is demonstrated using a one-way ANOVA test. Table 11 for case 12 and Table 12 for case 13 at 50% & 80% load display the ANOVA results. Figure 24 displays the ANOVA figure for cases 12 and 13 at 50% and 80%. The suggested approach is statistically better than BBO and GWO, as indicated by the computed P-value being substantially less than 0.05. Figures 22 and 23 show box plots of cases 9 to 11 for 50% & 80% load and case 17 for 60% & 100% load of various algorithms. It shows that the suggested DTBO algorithm is superior than BBO and GWO. Additionally, Fig. 25 displays the error bar for cases 14 through 16 for loads of 50% and 80% of various algorithms. Additionally, it shows that the proposed technique is statistically better than GWO and BBO.

## Conclusions

As a test system, this study uses the IEEE 57-bus and IEEE 118-bus network with thermal power sources, where integrates RES (wind & PV), ESS (AEFC & ultra capacitor) and FACTS (TCSC,TCPS & SVC) with frequency security constraints & FOPID controller. To determine the optimal solution for this OPF problem DTBO algorithm is proposed here. In the first system of this study when present only thermal, it demonstrates that, at lower loads, the generation cost is decreased in the absence of frequency security constraints, but at higher loads, frequency deviation is increased. The generation cost increases at lower loads but frequency deviation reduces when frequency security requirements are introduced to the study’s second test system. In the third system of this study while introduced FACTs devices (TCSC,TCPS & SVC) with frequency security constraint, simultaneously decreased the cost of generation & emission and also decreased the frequency variation. In the fourth system of this study when incorporate RES, ESS, FACTs devices (TCSC,TCPS & SVC) with frequency security constraint & FOPID controller, further decreased the cost of generation & emission and also decreased the frequency variation. The total cost of generation and emission are decreased by 16.59%, 34.95%, respectively and frequency variation is decreased (OS-12.23% & SS-13.11%) at 50% load. After integrating RES, ESS & FACTS with frequency security constraint using FOPID controller, at 50% load the total fuel cost & emission are reduced by 36.41%, 41% respectively and frequency variation are reduces OS-97.43%, US-61.44%, SS-80.73% for area 1 and OS-96.46%, US-56.91%, SS-79.58% for area 2. And at 80% load the total fuel cost & emission are decreased by 26.11%, 36% and frequency deviation decreases OS-97.43%, US-75.32%, SS-82.24% for area 1 and OS-99.89%, US-72.82%, SS-82.39% for area 2. According to the simulation outcomes, it can be concluded that the DTBO tuned FOPID is better than the BBO and GWO. Therefore, the frequency security restriction must be implemented by incorporating RES, ESS & FACTs devices (TCSC, TCPS, and SVC) in order to decrease generation cost, emission and improve frequency variation at any load. In future this system may be upgraded with other ESS and FACT devices to further reduce emissions and producing costs.

## Data Availability

Not Applicable.(This manuscript does not report data generation or analysis.)

## Appendix A

$${{{B}}_{{1}}},{{{B}}_{{2}}}={0}.{42}{{{5}}_{{}}}{p}.{u} {MW}/{Hz};$$ $${{{R}}_{{1}}},{{{R}}_{{2}}}={2}.{4Hz}/{pu};$$ $${{{T}}_{{G1}}}={0}.{08s};$$ $${{{T}}_{{T1}}}={0}.{3s};$$ $${{{T}}_{{r1}}}={10s};$$ $${{{K}}_{{r1}}}={0}.{3s};$$ $${{{K}}_{{P1}}}= {120Hz}/{puMW};$$ $${{{K}}_{{P2}}}={120Hz}/{pu}~{MW};$$ $${{{T}}_{{P1}}}={{{T}}_{{P2}}}$$ $$={20s};{Ptie12}= {200}{MW};$$ $${a12}=-{1}, {{{X}}_{{G}}}={0}.{6s};$$ $${{{Y}}_{{G}}}={1}.{1s};$$ $${{{K}}_{{T1}}}={{{K}}_{{T2}}}={0}.{6};$$ $${{{T}}_{{RH}}}={41}.{6}{s},{{{T}}_{{R}}}= {5}{s};$$ $${{{T}}_{{GH}}}={0}.{51}{s},{{{T}}_{{W}}}={1}{s},{{{K}}_{{H1}}}={{{K}}_{{H2}}}={0}.{3}, {{{C}}_{{g}}}={1};$$ $${{{b}}_{{g}}}={0}.{049s};$$ $${{{T}}_{{F}}}={0}.{239s};$$ $${{{T}}_{{CR}}}={0}.{01s};$$ $${{{T}}_{{CD}}} ={0}.{2s};$$ $${RLP}=\pm {0}.{02p}.{u}( {Mw})$$ $${{a}}={900};$$ $${{b}}={-18};$$ $${{d}}={50};$$ $${{d}}={50};$$ $${50\% loading condition}$$.

## Appendix B

$$A = \left[ {\begin{array}{*{20}{c}} { - 1/{T_{P1}}}& 0& { - {K_{P1}}/{T_{P1}}}& {{K_{P1}}/{T_{P1}}}& 0& {{K_{P1}}/{T_{P1}}}& 0& 0& 0& 0& 0\\ 0& { - 1/{T_{P2}}}& { - {K_{P2}}{\alpha _{12}}}& 0& 0& 0& 0& {{K_{P2}}/{T_{P2}}}& 0& {{K_{P2}}/{T_{P2}}}& 0\\ {2\pi {T_{12}}}& { - 2\pi {T_{12}}}& 0& 0& 0& 0& 0& 0& 0& 0& 0\\ 0& 0& 0& {-1/{{T}_{r}}}& 0& 0& 0& {{K}_{r}/{{T}_{r}}}& (1/{{T}_{r}}-{{K}_{r1}/{{T}_{r1}}})& 0& 0\\ { - 1/{R_1}{T_{g1}}}& 0& 0& 0& { - 1/{T_{g1}}}& 0& 0& 0& 0& 0& 0\\ 0& 0& 0& 0& 0& { - (a - c)}& {\left( {\frac{{{a^2}}}{{{b^2}}} - \frac{{ac}}{b} + d} \right) }& 0& 0& 0& 0\\ { - \frac{b}{{{R_1}}}}& 0& 0& 0& 0& { - (a - c)}& { - (a/b)}& 0& 0& 0& 0\\ 0& 0& 0& 0& 0& {-1/{{T}_{r}}}& 0& 0& 0& {{K}_{r}/{{T}_{r}}}& (1/{{T}_{r}}-{{K}_{r1}/{{T}_{r1}}})\\ 0& { - 1/{R_3}{T_{G3}}}& 0& 0& 0& 0& 0& 0& { - 1/{T_{g3}}}& 0& 0\\ 0& 0& 0& 0& 0& 0& 0& (-1)& 0& {{K}_{WT1}}-{\frac{{{T}_{TP,WT1}}{{K}_{WT1}}}{{T}_{P2,WT2}}}& {\frac{{{T}_{TP,WT1}}{{K}_{WT1}}}{{T}_{P2,WT2}}}\\ 0& { - 1/{{T_{TP2,WT2}}}}& 0& 0& 0& 0& 0& 0& 0& 0& {1/{{T_{TP2,WT2}}}} \end{array}} \right]$$

$$B = \left[ {\begin{array}{*{20}{c}} 0& 0\\ 0& 0\\ 0& 0\\ 0& 0\\ {\frac{{a_{11}^{'}}}{{{T_{g1}}}}}& 0\\ 0& 0\\ {\frac{{a_{12}^{'}b}}{a}}& 0\\ 0& 0\\ 0& 0\\ 0& {\frac{{{a_{21}}}}{{{T_{g3}}}}}\\ 0& {\frac{{a_{22}^{'}}}{{{T_{TP2,WT2}}}}} \end{array}} \right] ;\Gamma = \left[ {\begin{array}{*{20}{c}} { - \frac{{{K_{PS}}}}{{{T_{PS}}}}}& { - \frac{{{K_{PS}}}}{{{T_{PS}}}}}& 0& 0\\ 0& 0& { - \frac{{{K_{PS}}}}{{{T_{PS}}}}}& { - \frac{{{K_{PS}}}}{{{T_{PS}}}}}\\ 0& 0& 0& 0\\ 0& 0& 0& 0\\ {\frac{{cp{f_{11}}}}{{{T_{g1}}}}}& {\frac{{cp{f_{12}}}}{{{T_{g1}}}}}& {\frac{{cp{f_{13}}}}{{{T_{g1}}}}}& {\frac{{cp{f_{14}}}}{{{T_{g1}}}}}\\ 0& 0& 0& 0\\ {\frac{{cp{f_{21}}b}}{a}}& {\frac{{cp{f_{21}}b}}{a}}& {\frac{{cp{f_{21}}b}}{a}}& {\frac{{cp{f_{21}}b}}{a}}\\ 0& 0& 0& 0\\ {\frac{{cp{f_{31}}}}{{{T_{g3}}}}}& {\frac{{cp{f_{32}}}}{{{T_{g3}}}}}& {\frac{{cp{f_{33}}}}{{{T_{g3}}}}}& {\frac{{cp{f_{34}}}}{{{T_{g3}}}}}\\ 0& 0& 0& 0\\ {\frac{{cp{f_{41}}}}{{{T_{TP2,WT2}}}}}& {\frac{{cp{f_{42}}}}{{{T_{TP2,WT2}}}}}& {\frac{{cp{f_{43}}}}{{{T_{TP2,WT2}}}}}& {\frac{{cp{f_{44}}}}{{{T_{TP2,WT2}}}}} \end{array}} \right] ;{A_d} = \left[ {\begin{array}{*{20}{c}} 0& 0& 0& 0& 0& 0& 0& 0& 0& 0& 0\\ 0& 0& 0& 0& 0& 0& 0& 0& 0& 0& 0\\ 0& 0& 0& 0& 0& 0& 0& 0& 0& 0& 0\\ 0& 0& 0& 0& 0& 0& 0& 0& 0& 0& 0\\ {\frac{{{K_{P1}}{\beta _1}ap{f_{11}}}}{{{T_{g1}}}}}& {\frac{{{K_{P1}}{\beta _1}ap{f_{11}}}}{{{T_{g1}}}}}& 0& 0& 0& 0& 0& 0& 0& 0& {\frac{{{K_I}_1ap{f_{11}}}}{{{T_{g1}}}}}\\ 0& 0& 0& 0& 0& 0& 0& 0& 0& 0& 0\\ {\frac{{{K_{P1}}{\beta _1}ap{f_{12}}a}}{b}}& {\frac{{{K_{P1}}ap{f_{12}}a}}{b}}& 0& 0& 0& 0& 0& 0& 0& 0& {\frac{{{K_{I1}}ap{f_{12}}a}}{b}}\\ 0& 0& 0& 0& 0& 0& 0& 0& 0& 0& 0\\ {\frac{{{K_{P2}}{\beta _2}ap{f_{21}}}}{{{T_{g3}}}}}& {\frac{{{K_{P2}}ap{f_{21}}}}{{{T_{g3}}}}}& 0& 0& 0& 0& 0& 0& 0& 0& {\frac{{{K_{I2}}ap{f_{21}}}}{{{T_{g3}}}}}\\ 0& 0& 0& 0& 0& 0& 0& 0& 0& 0& 0\\ {\frac{{{K_{P2}}{\beta _2}ap{f_{22}}}}{{{T_{TP2,WT2}}}}}& {\frac{{{K_{P2}}{\beta _2}ap{f_{22}}}}{{{T_{TP2,WT2}}}}}& 0& 0& 0& 0& 0& 0& 0& 0& {\frac{{{K_{I2}}ap{f_{22}}}}{{{T_{TP2,WT2}}}}} \end{array}} \right]$$
